# Polarized, V-Shaped, and Conjoined Biscoumarins: From Lack of Dipole Moment Alignment to High Brightness

**DOI:** 10.1021/acs.joc.2c00232

**Published:** 2022-04-12

**Authors:** Łukasz Kielesiński, Irena Deperasińska, Olaf Morawski, Kateryna V. Vygranenko, Erik T. Ouellette, Daniel T. Gryko

**Affiliations:** † Institute of Organic Chemistry of Polish Academy of Sciences, Kasprzaka 44/52, 01-224 Warsaw, Poland; ‡ Institute of Physics of Polish Academy of Sciences, Al. Lotników 32/46, 02-668 Warsaw, Poland; § Department of Chemistry, University of California, Berkeley, 420 Latimer Hall, Berkeley, California 94720, United States; ∥ Chemical Sciences Division, Lawrence Berkeley National Laboratory, Berkeley, California 94720, United States

## Abstract

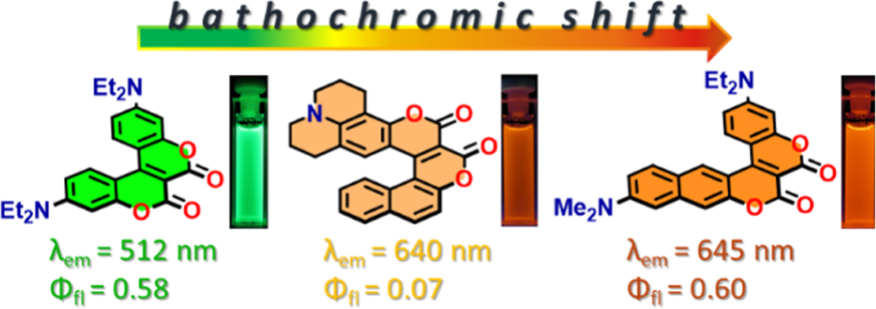

Eleven conjoined coumarins possessing a chromeno[3,4-*c*]chromene-6,7-dione skeleton have been synthesized via the reaction of electron-rich phenols with esters of coumarin-3-carboxylic acids, catalyzed by either Lewis acids or 4-dimethylaminopyridine. Furthermore, Michael-type addition to angular benzo[*f*]coumarins is possible, leading to conjugated helical systems. Arrangement of the electron-donating amino groups at diverse positions on this heterocyclic skeleton makes it possible to obtain π-expanded coumarins with emission either sensitive to, or entirely independent of, solvent polarity with large Stokes shifts. Computational studies have provided a rationale for moderate solvatochromic effects unveiling the lack of collinearity of the dipole moments in the ground and excited states. Depending on the functional groups present, the obtained dyes are highly polarized with dipole moments of ∼14 D in the ground state and ∼20–25 D in the excited state. Strong emission in nonpolar solvents, in spite of the inclusion of a NO_2_ group, is rationalized by the fact that the intramolecular charge transfer introduced into these molecules is strong enough to suppress intersystem crossing yet weak enough to prevent the formation of dark twisted intramolecular charge transfer states. Photochemical transformation of the dye possessing a chromeno[3,4-*c*]pyridine-4,5-dione scaffold led to the formation of a spirocyclic benzo[*g*]coumarin.

## Introduction

**Figure 1:**
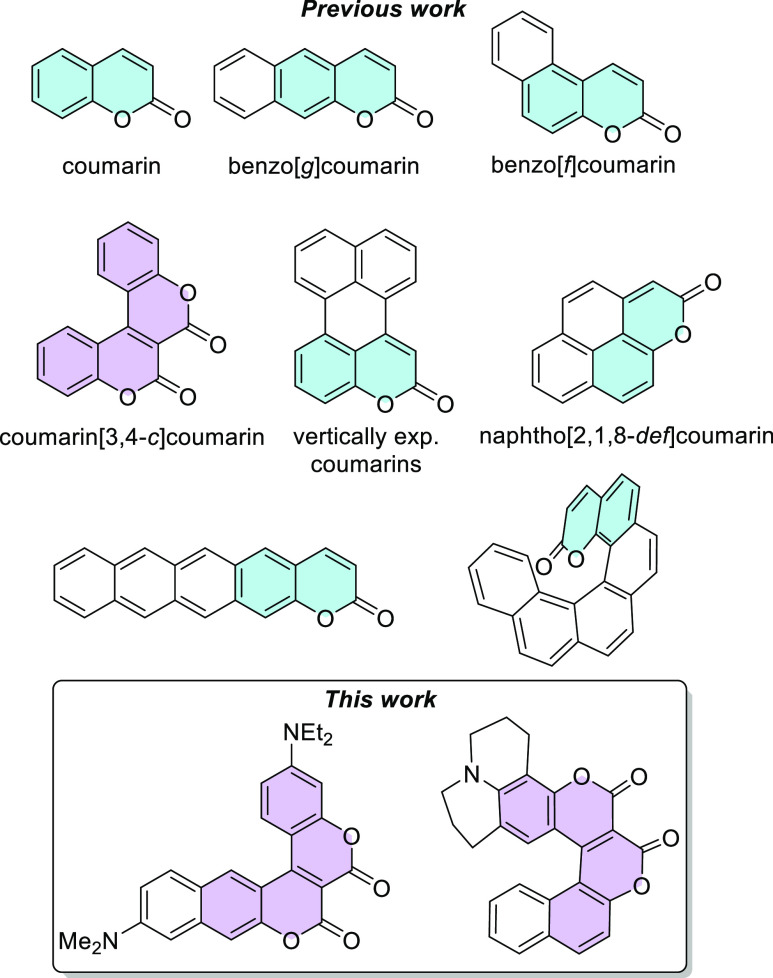
Structures of various π-expanded coumarins.

Although coumarins were first synthesized almost 150 years ago,^1^ they are still of interest to many researchers worldwide. This fact is mainly related to their very wide range of applications. Many groups of coumarins exhibit biological activity, including anti-inflammatory, antifungal, antibacterial, or dermal photosensitizing properties, that make them useful in the medicinal and pharmaceutical industries.^2−4^ In addition to the features associated with their bioactivity, coumarins possess very interesting photophysical properties.^5−7^ These compounds are highly desirable for applications in solar cells,^8^ organic light-emitting diodes,^9^ and laser dyes^10^ due to their relatively simple, easily tunable structures, along with their high fluorescence quantum efficiencies, long decay times, and large Stokes shifts. Modification of the molecular structure of coumarins by extending the π-electron system strongly influences their electronic spectra. Consequently, appropriately substituted π-expanded coumarins exhibiting bathochromic shifts in the absorption and emission spectra are increasingly used in biological imaging, thanks to the deeper tissue penetration depth of this spectral region (Figure 1).^11^ A significant portion of research has been dedicated to fluorescent benzo[*g*]coumarin-based probes applicable for two-photon microscopy.^12^ Another interesting feature of π-expanded coumarins is the opportunity to apply them in the construction of new classes of optoelectronic materials.^13^

In the last few years, empowered by synthetic advances, previously unknown π-expanded coumarins have been explored, including those exhibiting a helical structure (Figure 1).^14^ Among the significant number of reported skeletons, coumarin[3,4-*c*]coumarins (chromeno[3,4-*c*]chromene-6,7-diones), that is, V-shaped conjoined biscoumarins, have attracted the most attention. Although known since 1984,^15−17^ they have only recently experienced a renaissance^18−22^ due to more straightforward synthetic methods becoming available.^23,24^ Inspired by straightforward and programmable preparation of coumarin[3,4-*c*]coumarin, we reasoned that modulation of photophysical properties can be achieved via employing π-expanded coumarins as building blocks in this two-component reaction. The simultaneous goal of this study was to design V-shaped biscoumarins with large dipole moments exceeding 10 debye, a value being in the ground electronic state usually an upper limit for molecules with large solvatochromism or showing dual emission as a result of twisted intramolecular charge transfer.^25−29^ This can be intuitively understood as the polarized moieties of a conjoined molecule contributing to the total dipole of the V-shaped structure (two vectors  and  add up to the resultant ). Such a description is, however, very oversimplified because it completely ignores the electronic coupling within the conjoined system and reduces interactions with Coulombic forces, which are further truncated to the dipole–dipole term only. At short distances, the multipole expansion is not exact even if it takes several orders, so the simple picture with dipoles is not valid. Therefore, a careful experimental study and a detailed theoretical exploration with quantum chemistry calculations are required for understanding the photophysics and optical properties of the V-shaped coumarins.

## Results and Discussion

### Design and Synthesis

**Scheme 1:**
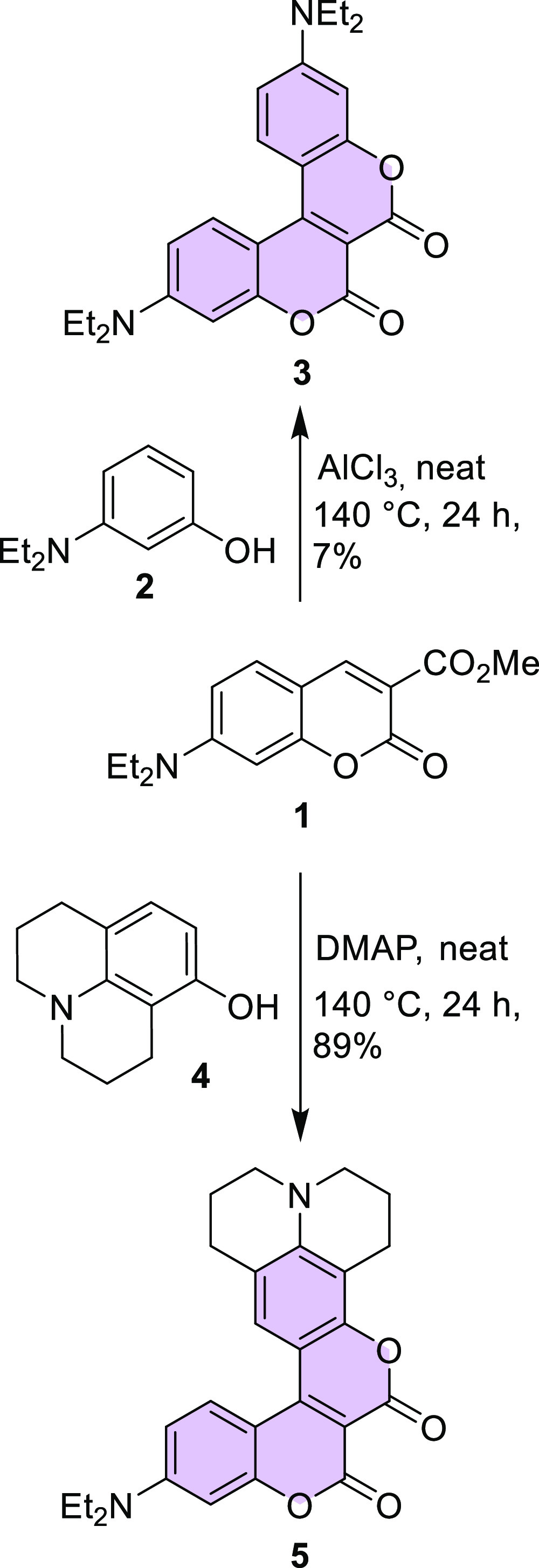
Synthesis of Biscoumarins **3** and **5**

Harnessing the propensity of strongly polarized benzo[*g*]coumarins to have large dipole moments originating from linearly extended conjugation was chosen as one of the key strategies to achieve our goal. We hypothesized that replacing one coumarin unit in a chromeno[3,4-*c*]chromene-6,7-dione scaffold with benzo[*g*]coumarin would lead to conjoined coumarins possessing bathochromically shifted emission and large dipole moments. Simultaneously, starting from benzo[*f*]coumarin may deliver curved coumarins analogous to [5]helicene. Our synthetic strategy capitalized on the fact that coumarins with an ester group in the 3 position form V-shaped condensation products when heated with reactive phenols or amidines in the presence of a catalyst, such as Lewis acids or some types of organic bases.^23,30^ By virtue of the electronic demands of these reactions, the nucleophilic partner has to possess two electron-donating groups. Thus, the structural possibilities leading to various electronic configurations have to be realized by varying the electrophilic substrate, that is, coumarin. The project started from the synthesis of dyes **3** and **5** possessing a *C*_2*v*_-symmetric scaffold with two amino groups present, dye **7** bearing a NO_2_ group and dye **8**. In all cases, the motivation lied in having conjoined biscoumarins with various arrangements of electron-donating and -withdrawing groups for comparison of photophysical properties. Compound **3** was obtained by the reaction of methyl 7-(diethylamino)-2-oxo-chromene-3-carboxylate (**1**) and 3-diethylaminophenol (**2**) in the presence of AlCl_3_ as a catalyst. Despite the optimization studies carried out (type of catalyst, reaction temperature, and time), after 24 h at 140 °C, the conversion was still low and the desired compound was isolated in only 7% yield (Scheme 1). The low yield prompted us to attempt to identify side-products in this reaction. Unfortunately, however, these attempts were futile, mostly because the presence of multiple, unstable side-products, which could not be isolated in a pure state. Mass spectrometry of combined fractions containing side-products have revealed that there are no regioisomeric conjoined coumarins present. On the other hand, biscoumarin **5** was obtained in significantly higher yield simply by changing the catalyst to *N*,*N*-dimethylpyridine-4-amine (DMAP) (Scheme 1). The presence of only one reactive position in the structure of 8-hydroxyjulolidine has plausibly the biggest impact on this yields’ difference. Subsequently, we synthesized biscoumarin **8**, which differs by the position of an amino group in one of the coumarin subunits. We wanted to investigate how the location of the donor substituent influences the photophysical properties when compared to biscoumarins substituted in the 7 position. We were curious if a similar behavior would be observed to that of simple 6-amino and 7-aminocoumarins.^31^ Unfortunately, the reaction of 6-aminocoumarin derivatives with appropriate phenols was low-yielding and many side-products were observed.

**Scheme 2:**
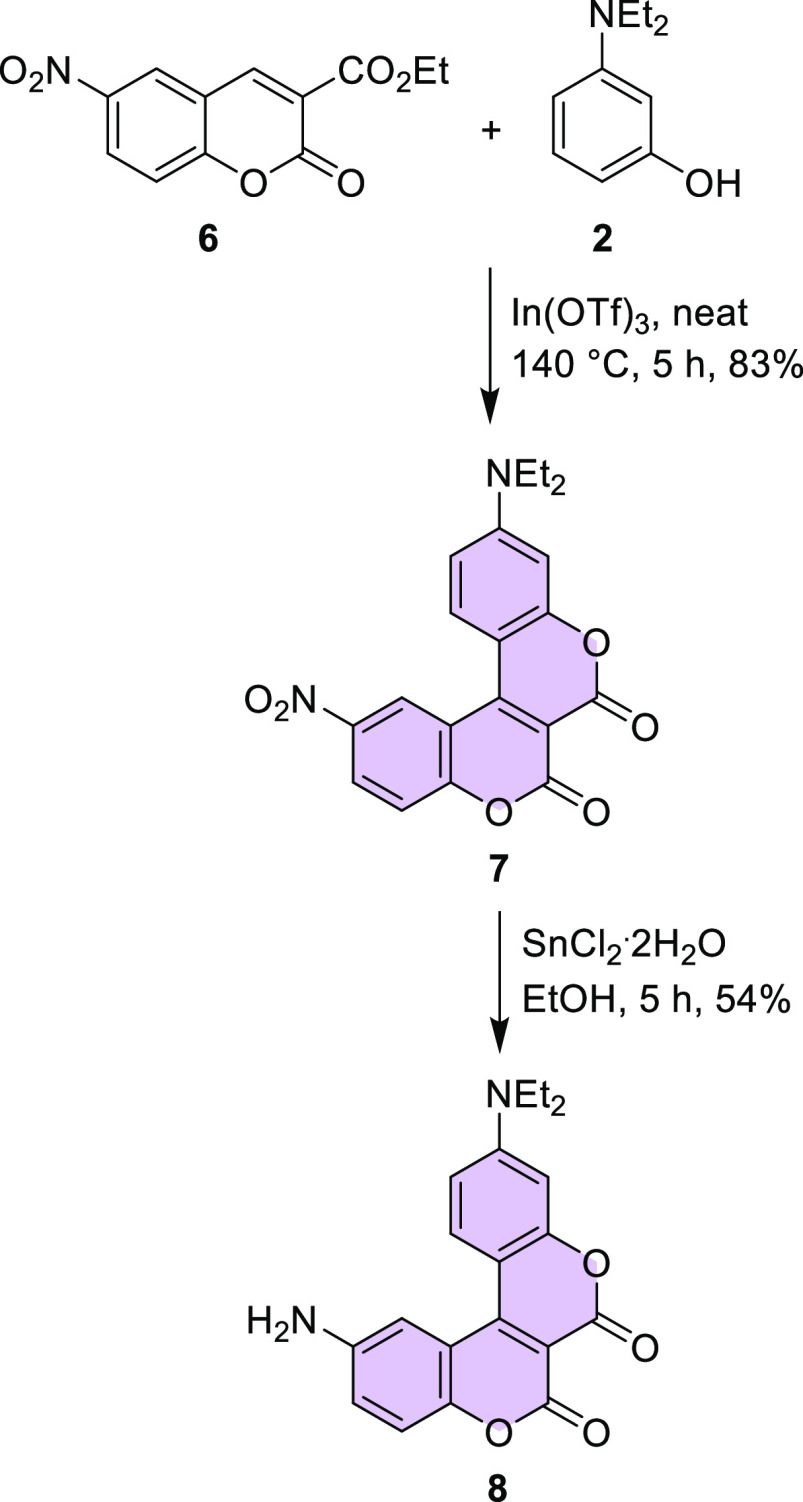
Synthesis of Biscoumarins **7** and **8**

Therefore, we started the synthesis of dyes **7** and **8** by obtaining compound **6**, which was subsequently reacted with 3-diethylaminophenol (**2**) in the presence of indium triflate (In(OTf)_3_) (Scheme 2). The reduction of the nitro group with tin(II) chloride led to the desired coumarin **8** in good overall yield.^32^

**Scheme 3:**
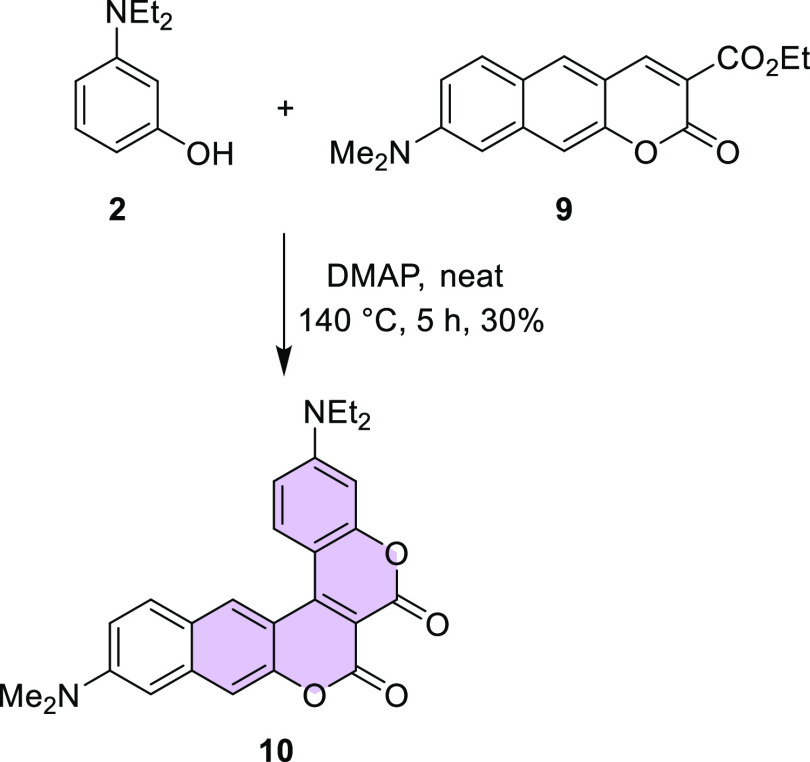
Synthesis of Biscoumarin **10**

We then turned our attention to benzo[*g*]coumarins as starting materials.^12,33^ We started the synthesis by preparation of the appropriately substituted benzo[*g*]coumarin **9** using methodology developed by Ahn and co-workers.^34^ In the crucial step, we carried out the reaction with 3-diethylaminophenol (**2**) in the presence of different catalysts, such as In(OTf)_3_, Al(OTf)_3_, AlCl_3_, FeCl_3_, and others Lewis acids, but the best results were obtained with DMAP (Scheme 3).

**Figure 2:**
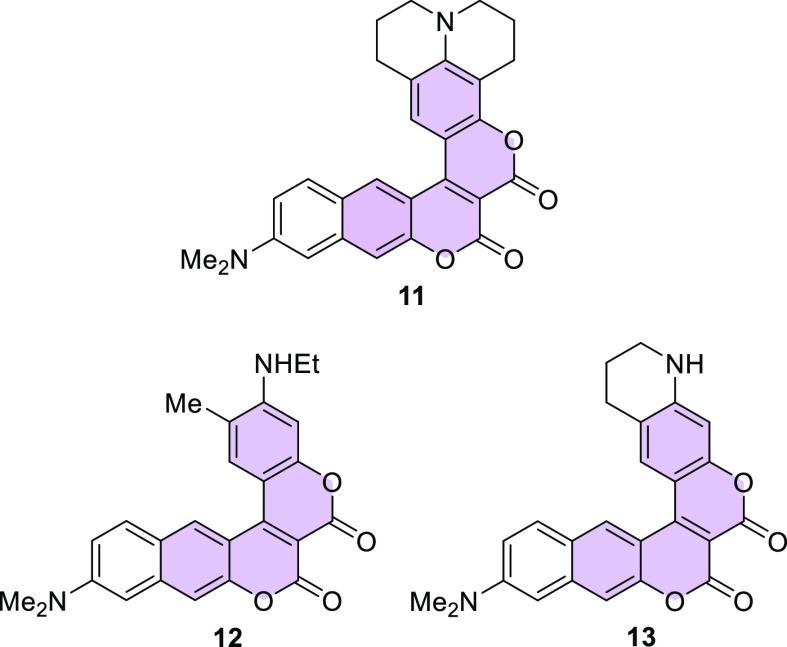
Structures of biscoumarins **11**–**13**.

Next, this approach was applied to a broader group of substrates. Using the same set of conditions and 8-hydroxyjulolidine (**4**), 3-(ethylamino)-*p*-cresol, and 7-hydroxy-1,2,3,4-tetrahydroquinoline, we synthesized compounds **11**, **12**, and **13** in 39, 36, and 41% yields, respectively (Figure 2). The yields in the range of 30–40% were related to the formation of many side-products, which were observed during the reaction. It is noteworthy that conversion of aminophenol was 100% in all these cases.

**Scheme 4:**
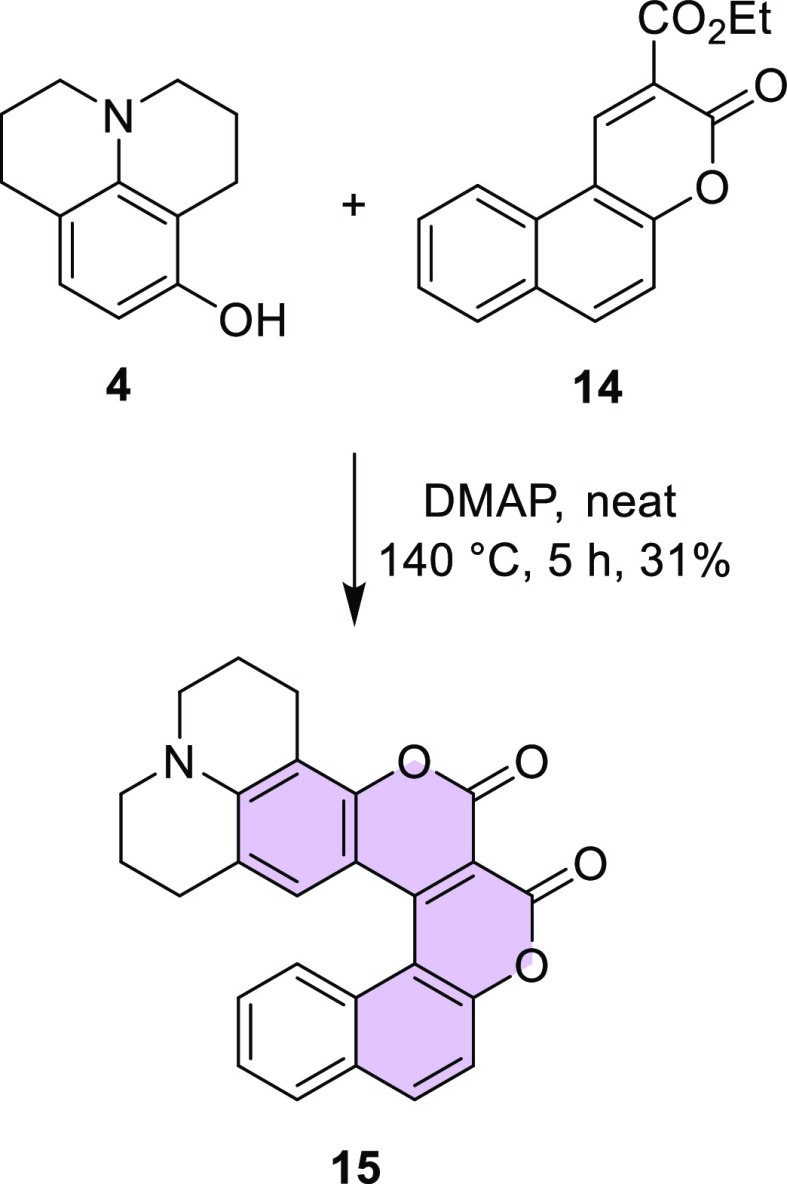
Synthesis of Biscoumarin **15**

These results encouraged us to apply this strategy to benzo[*f*]coumarins as electrophilic partners, which should deliver conjoined coumarins bearing the [5]helicene motif. We expected that it would be a more difficult challenge, mainly due to a greater steric hindrance. However, coumarin **14** in reaction with 8-hydroxyjulolidine (**4**) gave coumarin **15** in acceptable yield (Scheme 4).

**Figure 3:**
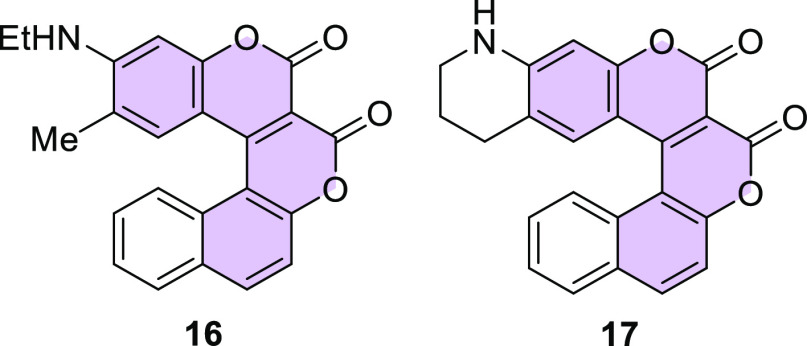
Structures of biscoumarins **16** and **17**.

By analogy, coumarins **16** and **17** were synthesized using appropriate phenols (Figure 3), although the yields were lower (18% in both cases) when compared to molecules based on the benzo[*g*]coumarin core. We also carried out the reaction with 3-diethylaminophenol (**2**), but in this case we observed only trace amounts of product, regardless of the catalyst used, which might be a result of the larger steric hindrance in relation to compound **10**.

**Scheme 5:**
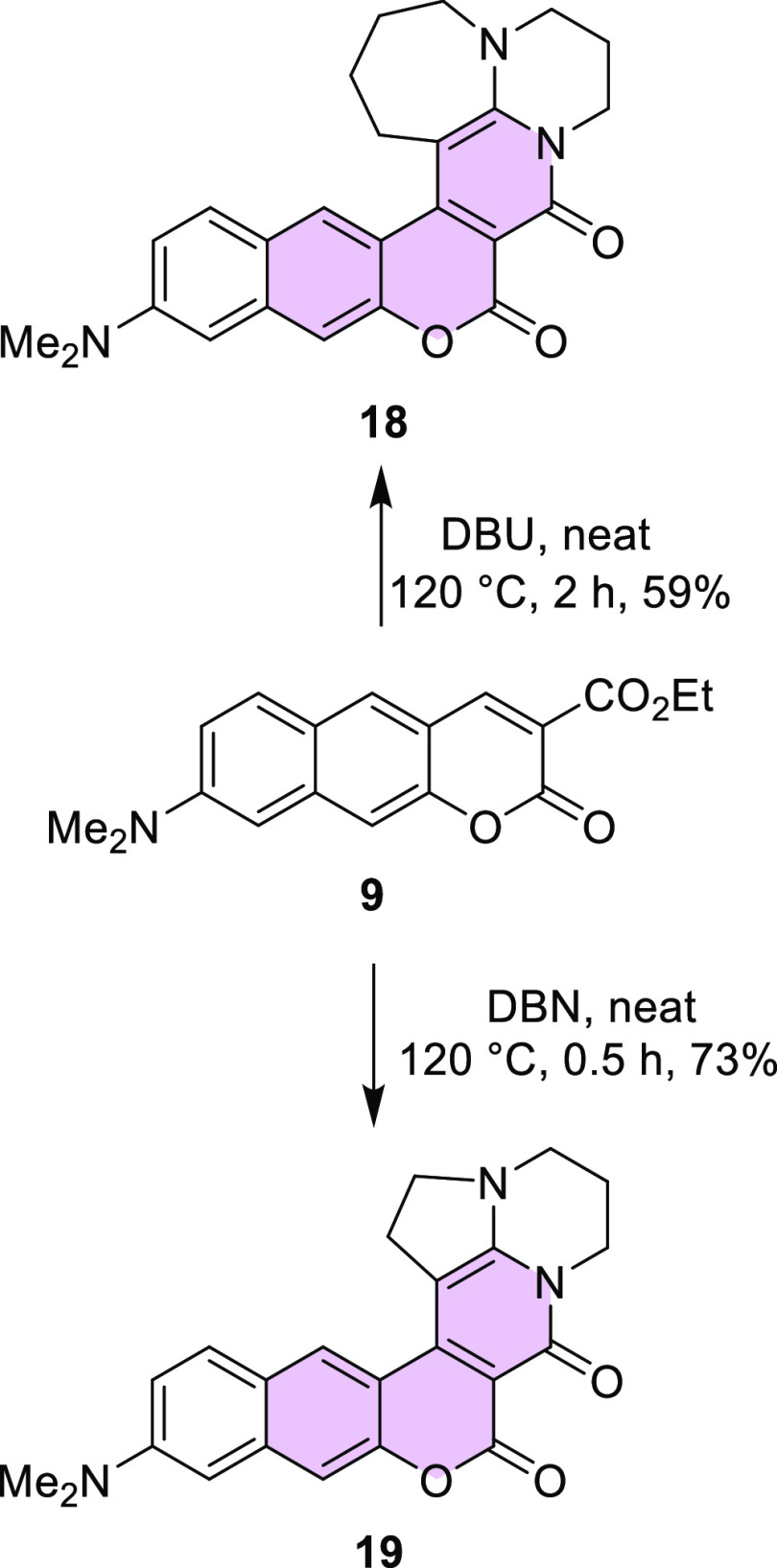
Synthesis of Compounds **18** and **19**

Finally, we attempted the cyclocondensation of benzocoumarins with 1,8-diazabicyclo[5.4.0]undec-8-ene (DBU) and 1,5-diazabicyclo[4.3.0]non-5-ene (DBN) employing the conditions developed a few years ago for the reaction of amidines with substituted coumarins.^24^ π-Expanded coumarins **18** and **19** were successfully prepared, although the reaction with DBU was longer and the yield of the product was slightly lower (59%) than in the case of reaction with DBN (Scheme 5). This is probably related to a smaller steric hindrance observed for the compound with five membered rings at the periphery of the system.

**Scheme 6:**
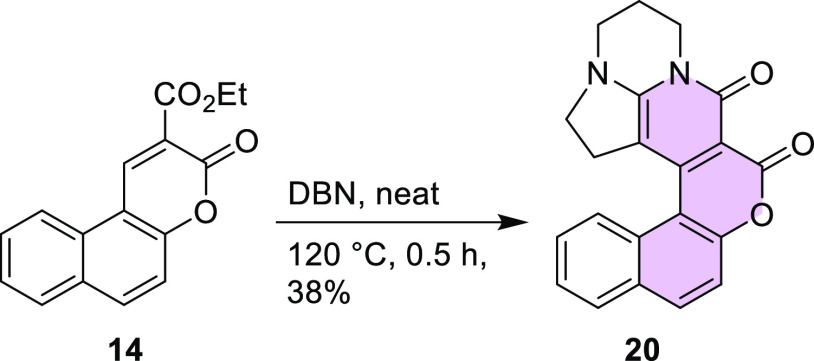
Synthesis of Compound **20**

The same conditions applied for benzo[*f*]coumarin **14** in reaction with DBN led to the product **20** in a slightly lower yield (38%) (Scheme 6), whereas in reaction with DBU only traces of desired product were observed. In both cases, the result was very likely due to the even larger steric hindrance caused by the seven membered ring.

**Figure 4:**
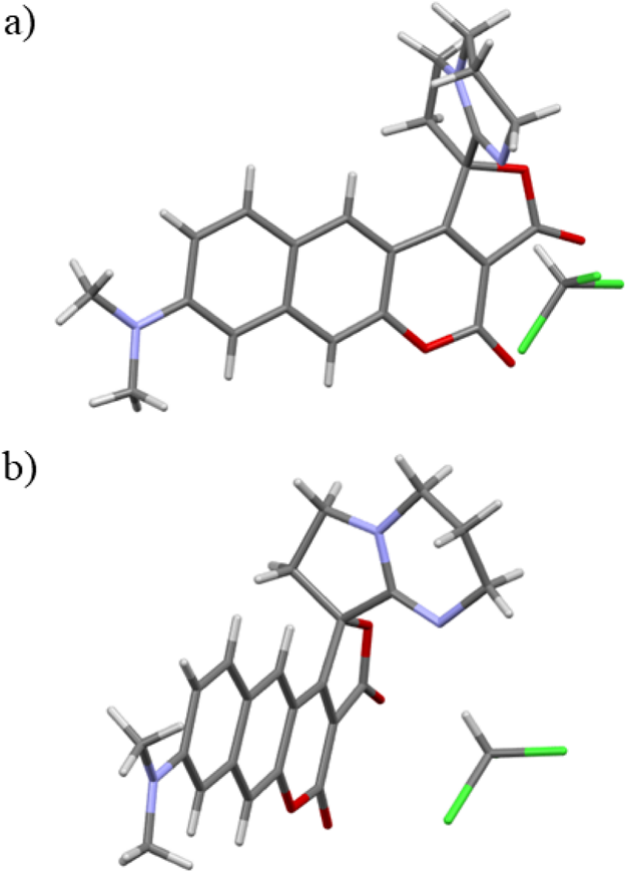
X-ray structure of spiro-coumarin **21**: (a) front view; (b) side view.

**Scheme 7:**
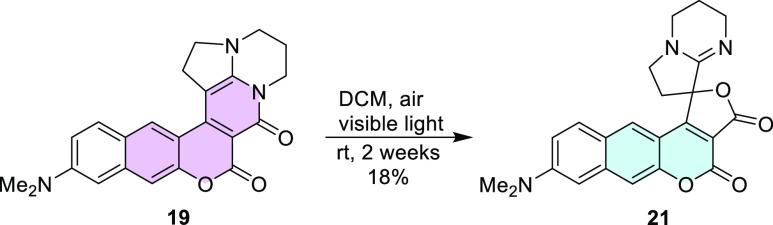
Light-Induced Formation of Spiro-coumarin **21**

Additionally, an interesting stability phenomenon was observed for compound **19**. We noticed that this molecule dissolved in dichloromethane (DCM) exposed to air and sunlight is transformed into many fluorescent products. Among them, from the reaction mixture, compound **21** could be isolated, but the yield was only 18% (Scheme 7). The structure of this compound was fully confirmed by X-ray analysis (Figure 4) as well as by NMR spectroscopy.

### Single-Crystal X-ray Diffraction Studies

Spiro-coumarin **21** was crystallized by slow diffusion of hexane into chloroform. The orange shards of 0.04 mm × 0.04 mm × 0.04 mm size were appropriate for X-ray analysis. The crystallographic structure of compound **21** is presented in Figure 4. The crystal belongs to the *P*2_1_/*n* space group. As shown in Figures 4 and S2, the part of molecule with the coumarin moiety fused with the five-membered lactone ring is planar, while the amidine moiety is positioned in a perpendicular arrangement. The dihedral angle between the coumarin core and the plane of the DBN ring is close to 90° (Figure S2). The molecule exhibits an antiparallel packing in the unit cell (Figure S1). The distance between the planes of the coumarin moiety of the two neighboring molecules is around 3.8 Å.

### Photophysical results

Photophysical properties were measured for compounds **3**, **5**, **7**, **8**, **10**–**13**, and **15–21** in nonpolar toluene (dielectric constant ε = 2.38), moderately polar DCM (ε = 8.93), and polar acetonitrile (ACN, ε = 38.8). Absorption and normalized fluorescence spectra for all molecules are presented in Figure S4 and Table 1. Compounds **3** and **5** possessing two electron-donating groups exhibited absorption and emission spectra in the range of 419–467 and 473–546 nm, respectively, which mostly correspond to the properties of other biscoumarin derivatives.^23^ Coumarin **7** bearing a NO_2_ group, as well as compound **8** possessing NH_2_ group, have bathochromically shifted spectra compared to previous molecules, which is particularly prominent in the case of emission (Figure S4).

**Table 1:** Spectroscopic Properties of Dyes **3**, **5**, **7**, **8**, **10**–**13**, and **15–21** Obtained in Toluene, DCM, and ACN

comp.	solvent	λ_abs_^max^ [nm]	λ_em_^max^ [nm]	ε [M^–^1cm^–1^]	Stokes shift [cm^–1^]	Φ_F_^a^	τ_aver_ [ns]	*k*_r_^b^ [ns^–1^]	*k*_nr_^c^ [ns^–1^]
**3**	toluene	419	473	51 500	2700	0.36	1.45	0.248	0.441
	DCM	453	494	60 900	1800	0.43	1.87	0.230	0.305
		422		56 000					
	ACN	451	512	56 200	2600	0.58	2.59	0.224	0.162
		420		52 700					
**5**	toluene	449	500	42 500	2300	0.37	2.06	0.180	0.306
		421		44 500					
	DCM	467	525	55 200	2400	0.44	3.96	0.111	0.141
		424		47 300					
	ACN	466	546	49 500	3100	0.83	4.36	0.190	0.039
		422		42 100					
**7**	toluene	469	532	24 900	2500	0.71	6.55	0.108	0.044
	DCM	480	560	31 100	3000	0.22	0.82	0.268	0.951
	ACN	471	576	29 000	3900	0.0007	0.06	0.012	16.66
**8**	toluene	441	557	27 100	4700	0.05	1.27	0.039	0.748
	DCM	459	545	35 500	3400	0.015	3.33	0.005	0.296
	ACN	451	647	30 200	6700	0.006	0.92	0.007	1.080
**10**	toluene	445	547	45 900	4200	0.68	1.45	0.469	0.221
	DCM	456	597	53 600	5200	0.76	5.17	0.147	0.046
	ACN	453	645	45 400	6600	0.60	5.16	0.116	0.078
**11**	toluene	454	544		3600	0.41	1.24	0.331	0.476
	DCM	498	586	46 500	3000	0.61	3.78	0.161	0.103
		466		48 900					
	ACN	494	634		4500	0.53	4.74	0.112	0.099
		465							
**12**	toluene	437	546		4600	0.59	3.69	0.160	0.111
	DCM	443	599	38 400	5900	0.65	5.58	0.116	0.063
	ACN	445	648	70 000	7000	0.63	4.82	0.131	0.077
**13**	toluene	443	545		4200	0.53	3.40	0.156	0.138
	DCM	449	597	45 800	5500	0.76	5.51	0.138	0.044
	ACN	451	645		6700	0.45	4.60	0.098	0.120
**15**	toluene	478	576	23 700	3600	0.56	5.59	0.100	0.079
		402		10 200					
		381		9100					
	DCM	495	607	33 400	3700	0.29	4.14	0.070	0.171
		401		12 800					
		381		12 000					
	ACN	491	640	25 100	4700	0.07	1.22	0.057	0.762
		399		9200					
		381		8800					
**16**	toluene	437	540		4400	0.23	2.23	0.103	0.345
	DCM	451	556	25 800	4200	0.27	3.41	0.079	0.214
		404		18 300					
	ACN	453	593	24 300	5200	0.14	2.18	0.064	0.394
		401		15 100					
**17**	toluene	449	549		4100	0.29	3.06	0.095	0.232
		404							
	DCM	462	567	24 600	4000	0.36	3.88	0.093	0.165
		404		14 400					
	ACN	463	597	24 500	4800	0.10	1.45	0.069	0.621
		400		12 500					
**18**	toluene	407	529		5700	0.015	0.35	0.043	2.814
	DCM	410	517	32 100	5000	0.04	0.50	0.080	1.920
	ACN	408	544	26 300	6100	0.03	0.49	0.061	1.980
**19**	toluene								
	DCM	429	488		2800	0.64	2.32	0.276	0.155
		405							
	ACN	429	517		4000	0.67	3.21	0.209	0.103
		404							
**20**	toluene	414	516		4800	0.08	1.86	0.043	0.495
		363							
		318							
	DCM	409	520	11 100	5200	0.09	2.17	0.041	0.419
		359		9800					
		318		22 400					
	ACN	409	537	9600	5800	0.08	2.44	0.033	0.377
		355		8400					
		316		21 000					
**21**	toluene	449	525		3200	0.71	5.10	0.139	0.057
		327							
	DCM	476	574	26 200	3600	0.74	5.50	0.135	0.047
		332		14 300					
	ACN	468	604	23 300	4800	0.78	5.60	0.139	0.039
		329		12 000					

a Fluorescence quantum yield measured using an integrating sphere.

b *k*_r_ = Φ_F_/τ_aver_.

c *k*_nr_ = 1/τ_aver_ – *k*_r_.

**Figure 5:**
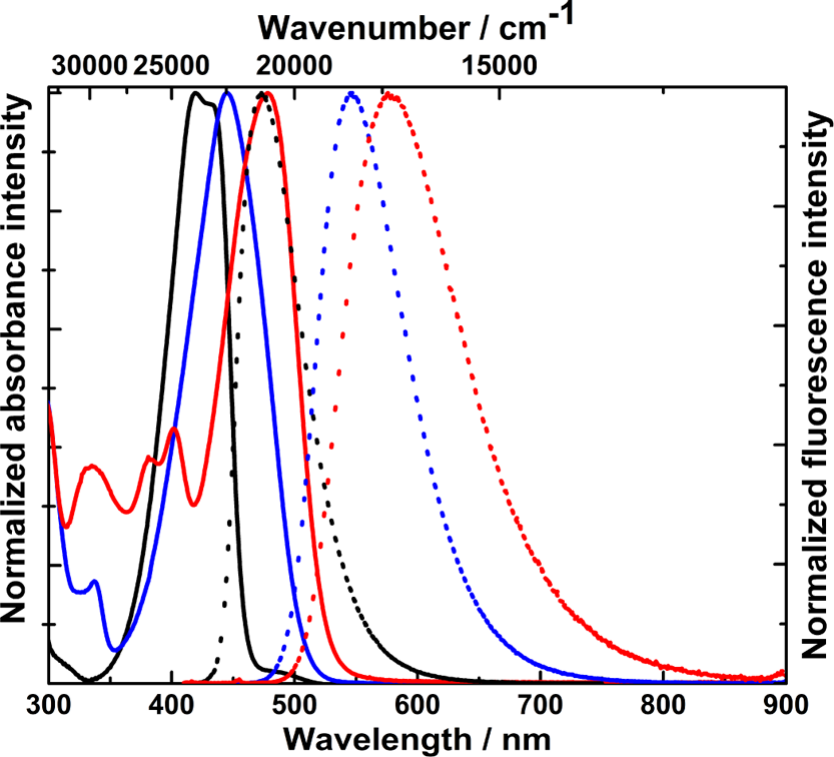
Absorption (solid line) and emission (dot line) spectra of dyes **3** (black, excited at 382 nm), **10** (blue, excited at 400 nm), and **15** (red, excited at 400 nm) measured in toluene.

Fluorescence spectra of the π-expanded conjoined coumarins containing benzo[*g*]- and benzo[*f*]coumarin cores are significantly more red-shifted than the spectra of simple V-shaped coumarins **3** and **5** (Figures 5 and S4) and are characterized by large Stokes shifts in the range of 3000–7000 cm^–1^ (Table 1). Additionally, the fluorescence spectra show solvatochromism when going from nonpolar to polar solvents, thereby suggesting that these compounds have large dipole moments in the excited state. For compounds **18**, **19**, and **20**, the location of absorption bands was very similar to those of other molecules described in the literature.^24,35^ Their emission spectra, however, are significantly shifted toward lower energy, which can be related to the strong electronic coupling present in the large π-expanded structure.

Most of the conjoined coumarins are strongly fluorescent in all solvents;, however, a difference in behavior is observed for some of them. Fluorescence quantum yields (Φ_F_) for compounds **3** and **5** are larger in polar solvents than in nonpolar solvents, where the nonradiative process rate *k*_nr_ increases (Table 1). This points to stabilization of the emissive S_1_ state in a polar environment. Compounds **7** and **8** exhibit low Φ_F_ in all solvents, which suggests an emission from a dark state giving a small value of the radiative rate, *k*_r_, and a large radiationless rate, *k*_nr_ (Table 1). Conjoined coumarins **10–13** possess fluorescence quantum yields in the range 0.41–0.76, while compounds **15**, **16**, and **17** are in the range from 0.07 to 0.56. A decrease of Φ_F_ with the increase of solvent polarity was observed for most of these compounds (Table 1). Compound **18** exhibited very low Φ_F_ (0.015–0.04), which could be related to the presence of a more flexible seven membered ring compared to **19**, which had a large Φ_F_ (0.64–0.67) and more rigid structure. Despite the fact that compound **20** possesses the same five-membered ring structure as molecule **19**, its fluorescence quantum yield is rather low (Table 1). This difference originates from the lack of an electron-donating group in its structure. Most of the conjoined coumarins have a one-exponential fluorescence decay profile with lifetimes ranging from 1 to 6 ns (Table S2). In a few cases, two component exponential decay is observed, which could be related to the presence of additional CT states. The values of the second fluorescence lifetime, τ_2_, were significantly longer indicating that the CT state lives longer than the LE state (Table S2).

Having this large library in hand, an interesting comparison can be made. Addition of a second amino group, that is, transforming the original chromeno[3,4-*c*]chromene-6,7-diones into conjoined coumarins possessing pure *C*_2*v*_ symmetry has a negligible hypsochromic effect on λ_abs_ (463 nm → 453 nm) and λ_em_ (528 nm → 494 nm). At the same time, however, it does influence Φ_F_, which is smaller for symmetric derivatives **3** and **5** although this increases and not decreases in polar solvents.

**Figure 6:**
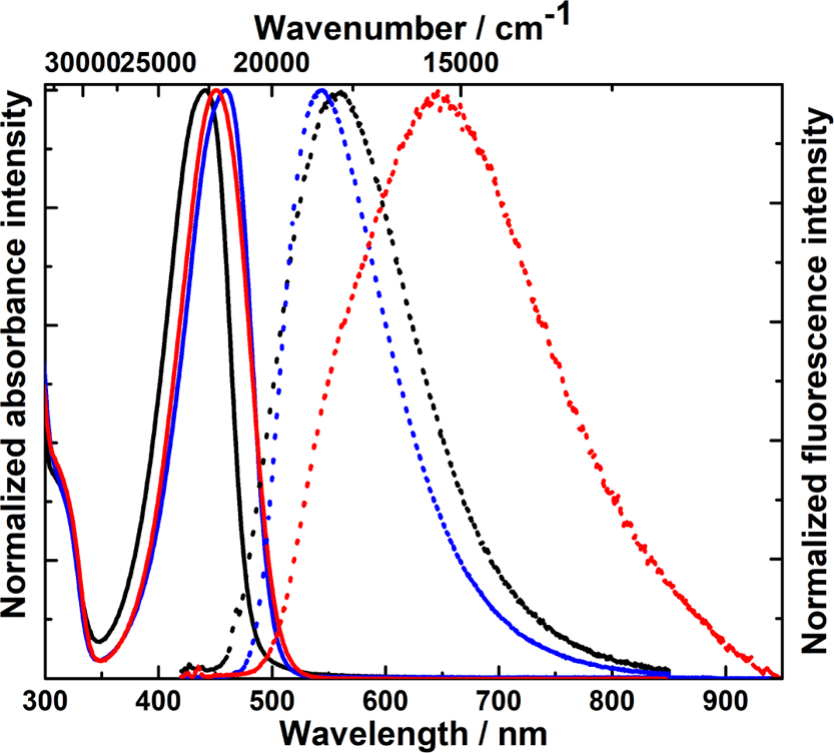
Absorption (solid line) and emission (dotted line) spectra of compound **8** (excited at 410 nm) measured in toluene (black), DCM (blue), and ACN (red).

The second interesting comparison is related to coumarins **8** versus **3** (Figure 6). It is well-known that moving the amino group from position 6 to position 7 has a profound effect on coumarin photophysical properties leading to a bathochromic shift of emission and a strong decrease in fluorescence quantum yield.^6b,6c,31e^ Combining 6-aminocoumarin with 7-aminocoumarin moieties in one dye leads to perplexing questions about “what will prevail”. As it turns out, the presence of an amino group at position 6 has a profound effect on decreasing the fluorescence quantum yield to 0.05 in nonpolar solvents and moves the emission in polar solvents to approx. 650 nm.

Breakthrough work by Ahn and co-workers has revealed that 8-dialkylamino-3-carboxymethyl-benzo[*g*]coumarin has absorption and emission markedly bathochromically shifted versus the electronically analogous 7-dialkylamino-3-carboxymethyl-coumarin, from 453 to 565 nm (in DCM), respectively.^12,34^ The direct comparison of dyes **3** and **10** shown that although λ_abs_ values are only slightly bathochromically shifted, the emission is red-shifted by 70–150 nm, depending on the solvent (Figure 5). At the same time in contrast to many classical coumarins, the fluorescence intensity neither decreases nor increases in polar solvents.

The variations of photophysical properties within the group of conjoined coumarins **10**, **11**, **13**, and **15**, all possessing the same core, but differing in the nature of the amino substituent at the coumarin moiety, are negligible.

The fundamental difference between helical conjoined coumarins **17**–**19** and their analogs derived from benzo[*g*]coumarins is obvious, that is, a minimal hypsochromic shift of both absorption and emission in toluene, but a larger in the case of ACN. The emission intensity is smaller, however, and it decreases sharply while moving to solvents with larger ε, although one can notice that dye **16** has a less sharp decrease in Φ_F_ in a polar solvent, which resembles earlier observations by Ahn^36^ as well as by Gryko and Sobolewski showing that coumarins possessing an ethylamino group at position 7 are fluorescent in polar environments.^37^

**Figure 7:**
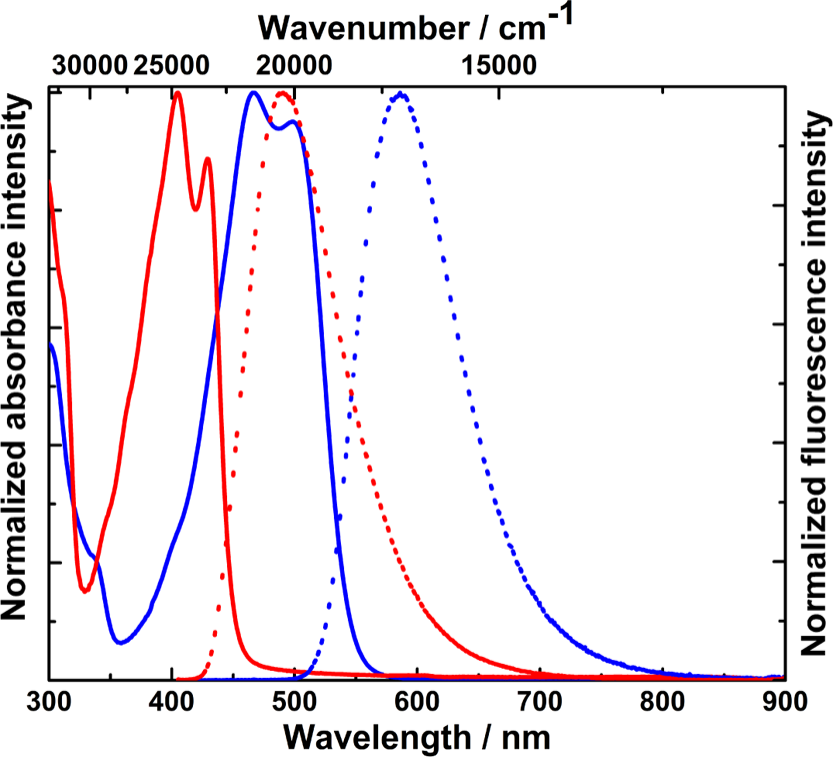
Absorption (solid line) and emission (dotted line) spectra of dyes **11** (blue, excited at 410 nm) and **19** (red, excited at 395 nm) measured in DCM.

Replacing the second coumarin moiety with a lactam shifts λ_abs_ of the resulting benzo[*g*]coumarins **18** and **19** hypsochromically (Figure 7). Similar to earlier observations performed for an analogous series possessing five-membered and seven-membered rings,^24^ emission of **19** is strong, whereas that of **18** is weak in solutions regardless of solvents’ polarity.

10-(Diethylamino)-2-nitro-chromeno[3,4-*c*]chromene-6,7-dione (**7**) possessing a NO_2_ group represents a very special case due to a recent surge of interest in fluorescent nitroaromatics.^38^ It is worth noting that fluorescence of this dye in toluene reaches 0.71 and decreases sharply in polar solvents (Table 1). This is in strong contrast to a previously described analog 3-(diethylamino)-10-nitro-chromeno[3,4-*c*]chromene-6,7-dione, which had undetectable fluorescence.^23^ The only difference between these two structures is the position of the NO_2_ group.

**Figure 8:**
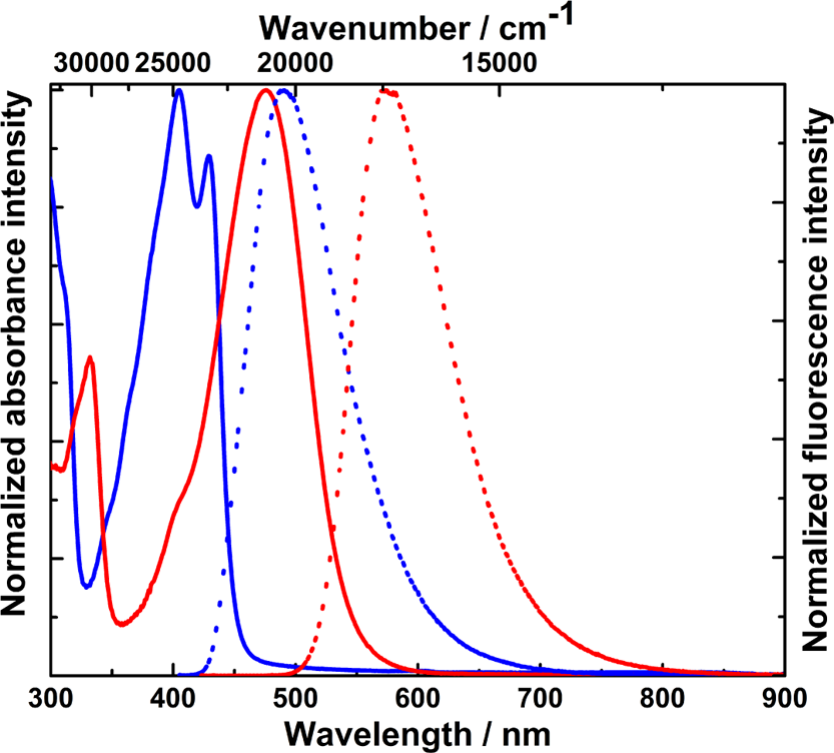
Absorption (solid line) and emission (dotted line) spectra of dyes **19** (blue, excited at 395 nm) and **21** (red, excited at 420 nm) measured in DCM.

Interesting photophysical properties are displayed by compound **21**. The maxima of absorption and emission spectra are in the range of 449–476 and 525–604 nm, respectively (Figure S4 and Table 1). The position of absorption bands is very similar to those of compounds **10**–**13** as well as molecules with a helical structure and a benzo[*f*]coumarin core. Although the emission bands of spiro-coumarin **21** are slightly hypsochromically shifted (Table 1), the opposite situation was observed compared to compounds **19** and **20** with the DBN moiety. In this case, both the absorption and emission spectra of spiro-coumarin **21** were significantly red-shifted (Table 1). Comparing molecule **19**, which is the direct precursor for spiro-coumarin **21**, the differences in the location of the absorption and emission maxima are 47 and 86 nm in DCM, respectively (Figure 8).

Spiro-coumarin **21**, like most of the presented conjoined V-shaped coumarins in this work, is also a strongly luminescent molecule. In all three solvents, Φ_F_ is in the range of 0.71–0.78. We see that in this case, the solvent polarity does not significantly influence these values. The large Φ_F_ correlates with relatively long lifetimes similar to the case of compound **19**. The radiative rate constant *k*_r_ is from 2.4 to 3.5 times larger than the nonradiative rate constant *k*_nr_, which suggests that radiative processes dominate (Table 1).

### Computational results

Calculations were performed using the Gaussian 16 package at the density functional theory (DFT and TDDFT) level with the PCM procedure for description of solvent effects.^39^ The optimization of molecular structures in the ground (S_0_) and electronic excited (S_1_) states was conducted with B3LYP and M06 functionals. The optimization has been confirmed by positive values of all calculated vibrational frequencies.

Calculations of the energy of electronic transitions S_0_ → S_1_ and S_1_ → S_0_ and the corresponding oscillator strengths for the considered coumarins in three solvents were performed to get insights into the photophysical processes. The calculations were preceded by the optimization of coumarin structures in both the S_0_ and S_1_ states. The results of these extensive calculations by the TD B3LYP/6-31G (d,p) method are summarized in Table S3 in the Supporting Information. In the same Table S3, the results of the calculations with the M06 functional can also be found due to the need for caution with the B3LYP functional known for its limitations. The use of the M06 functional leads to slightly higher transition energies, and in three cases (conjoined coumarins **7**, **8**, and **18**) it turned out to be more beneficial, but it did not change the conclusions resulting from the other calculations. The calculated oscillator strength and transition energy for the S_1_ → S_0_ transition were used to estimate the radiative transition rate constants *k*_r_. The calculated transition energies for absorption and fluorescence, as well as the radiative constants (Table S3) were correlated with the relevant experimental data (taken from Table 1), as shown in Figures S5–S7 in the Supporting Information. Most of the calculated transition energies are correlated within tolerable ±0.15 eV accuracy.^40^ Also, most of the values of the estimated radiative transition rate constants correlate well with the experiment. However, the correlation between the calculated experimental values for two coumarins **7** and **18** is not acceptable. Coumarin **7** in a nonpolar environment is characterized by high fluorescence efficiency, while the oscillator strength calculated for it is low. The opposite problem occurs with coumarin **18**. Thus, in both cases it was necessary to search for other (than those originally optimized) stable forms in the excited state.

#### General Outlook

The conjoined coumarins can be considered in terms of two coumarin cores, sharing one common central bond with coumarin **11** as an example (Figure S8). Steric interactions between the two moieties make them nonplanar structures. Characteristic features of these are large values of dipole moments. Results of the calculations presented in Table 2 reveal that all conjoined biscoumarins are highly polarized in the ground state and their dipole moments (≈14 D) are much larger than that of benzo[*g*]coumarin **9** (7 D)^33^ or **CoumMono** (≈10 D)^37^ structurally analogous to coumarins used for laser dyes and other purposes.^41^ In the excited state, the dipole moment increases to values around 20 D or larger (again larger than **CoumMono**(37) and comparable to dye **9**,^33^Table 2) and the optical transition is fully allowed in absorption and in emission, suggesting possible large fluorescence quantum yield, which is indeed observed (Table 1). Interestingly, the observed solvatochromic effects are rather moderate considering the high dipole moment. The reason is the noncollinearity of the dipole moments in the ground and excited states, with an angle of ≈30° between them (Table 2). The reason for this noncollinearity is the specific character of the electronic excitation in the conjoined V-shaped coumarins.

**Table 2:** Calculated (B3LYP) Values of Electronic Transition Energies (*E*_abs_ and *E*_em_), Oscillator Strengths (*f*), and Dipole Moments of Conjoined Coumarins in Toluene: μ_g_ in the Ground State (S_0_) and μ_e_ in the Excited (S_1_) State^a^

	S_0_ state			S_1_ state			S_1_ vs S_0_		
comp.	*E*_abs_ [nm]	*f*	|μ_g_| [D]	*E*_em_ [nm]	*f*	|μ_e_| [D]	α [deg]	|μ_e_| – |μ_g_| [D]	|μ_e_ – μ_g_| [D]
**3**	412.5	0.8229	14.85	451.9	0.5716	19.32	29.9	4.47	3.52
**5**	425.8	0.7251	14.97	478.6	0.4891	19.48	30.5	4.51	2.65
**7**	458.3	0.0650	10.37						
**8**	442.1	0.2241	14.65	597.7	0.1038	19.24	30.0	4.59	3.76
**10**	476.1	0.5061	16.16	542.7	0.3056	24.17	33.5	8.01	3.40
**11**	475.7	0.5629	16.10	539.0	0.3311	23.90	33.3	7.80	3.82
**12**	472.5	0.4919	16.29	543.4	0.2879	24.18	32.7	8.17	3.90
**13**	473.6	0.4783	16.54	545.1	0.2809	24.45	32.1	7.91	3.88
**15**	454.6	0.4179	14.03	583.8	0.2165	19.94	30.9	5.91	3.00
**16**	424.4	0.4085	14.13	547.2	0.1994	19.12	29.6	4.99	3.28
**17**	430.9	0.3985	14.41	554.9	0.1979	19.33	28.5	4.93	3.37
**18**	418.0	0.5089	12.94						
**19**	405.3	0.5723	13.51	459.3	0.3772	19.51	38.1	6.00	3.38
**20**	399.7	0.1303	12.26	511.8	0.0705	15.91	37.7	3.65	3.23

a α is the angle between μ_g_ and μ_e_; |μ_e_| – |μ_g_| is the difference in scalar values of dipole moments; |μ_e_ – μ_g_| is the value of the vector difference between both vectors—this is the factor that determines the size of the solvatochromic effect, as it is known from the theory of the Lippert–Mataga solvent effect.^42^ Lippert–Mataga expressions are recalled in the Supporting Information.

**Figure 9:**
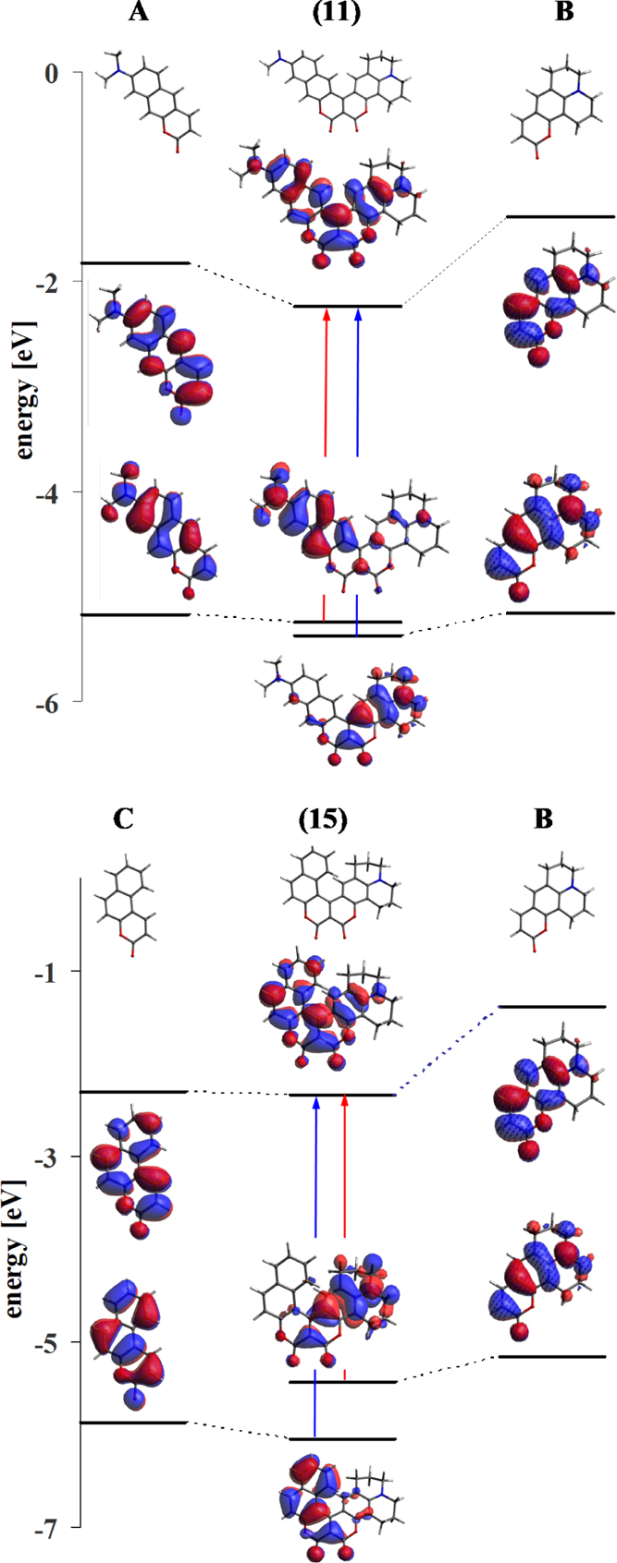
Molecular orbital energy diagrams with frontier orbitals of conjoined coumarins **11** and **15** and the character of the S_0_ → S_1_ transition (marked in red) and S_0_ → S_2_.

Frontier orbitals of conjoined coumarins share common features: their HOMOs are located on one of the components, while their LUMOs are shared. This is shown in the molecular energy diagrams for two coumarins **11** and **15** in Figure 9. Hence, the HOMO → LUMO transition is partially located on the components on which the HOMO is located, and partially it is a CT transition to the second component. Consequently, the electronic transition S_0_ → S_1_ in conjoined coumarins is characterized by transition moment vectors directed from one coumarin moiety to the second coumarin moiety. As a result, in the excited state of the conjoined coumarins, the direction of the dipole moment changes compared to the ground state, as shown in Table 2.

One of the components of both depicted conjoined coumarins is the same coumarin core, labeled B. The couplings between the HOMO components are not large and in both conjoined coumarins, they remain practically localized to one of the components. Due to the difference in ionization potential between these parts, in the A–B combination, the HOMO of coumarin **11** is located on A, while in the B–C combination, the HOMO of dye **15** is located on B. Therefore, the S_0_ → S_1_ transition in coumarin **11** is a transition partially located on A, and it takes place as a partial CT transition from A to B. The S_0_ → S_1_ transition in dye **15**, however, is partially located on B and is partially a CT transition from B to C. Due to the relatively small fission of HOMO and HOMO – 1 (each located on one of the coumarin moieties), the S_0_ → S_1_ and S_0_ → S_2_ transitions have similar energies (which is confirmed by experiment).

The oscillator strengths (Table 2 and Table S3) in almost all conjoined coumarins are large with values typical for fully allowed optical transitions. Oscillator strengths for the conjoined V-shaped coumarins are larger than for their constituent parts (Table S4), revealing strong coupling in the extended π-electron system. This is also manifested by significant lowering of the excited state energy transitions in the conjoined coumarins occurring at energies lower than in a single component molecule. In the large group of coumarins tested in this work, there were also cases that displayed properties different to those described above.

#### Individual Cases

**Figure 10:**
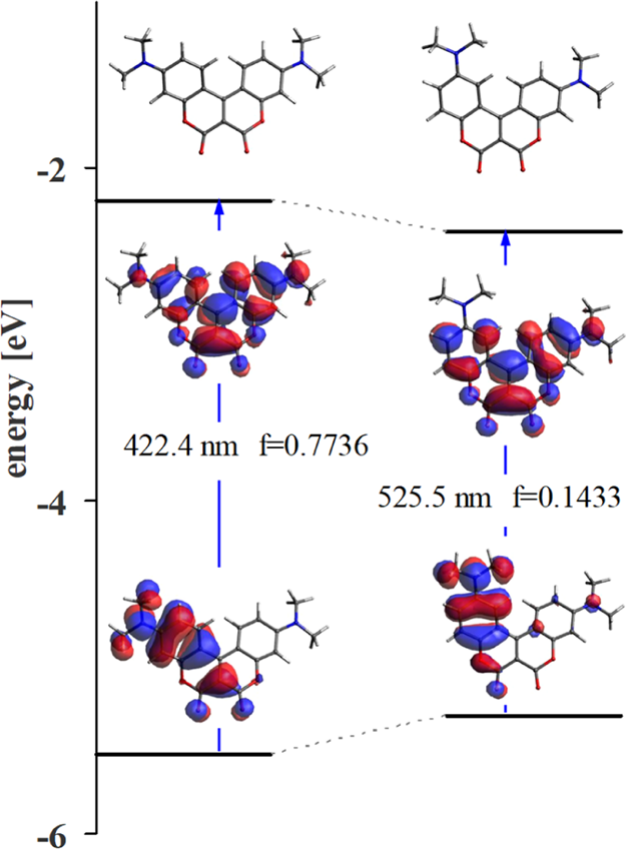
Influence of the position of the amino group in one of the subunits of conjoined coumarins **3** and **8** on the oscillator strength and the transition energy between states S_0_ → S_1_ (for better comparison between both coumarins, amine in the 6-position of dye **8** was computed with two Me groups).

Energy and oscillator strengths of the conjoined coumarins additionally depend on the position and nature of the substituted group. A decrease in the transition energy and oscillator strength of this transition is observed when changing the position of the amino group from the 7- to 6-position (coumarins **3** vs **8**) and is illustrated in Figure 10. The HOMOs in the conjoined biscoumarins **3** and **8** are highly localized and retain the shape of the orbitals from one of the components (7-aminocoumarin or 6-aminocoumarin). In contrast, the LUMOs are delocalized over both components and are the sum of the LUMOs of each component (Figure 10). Thus, the properties of HOMO → LUMO transitions in individual components are transferred to coumarins **3** and **8**, resulting in small oscillator strength and small Φ_F_ in the case of dye **8**.

**Figure 11:**
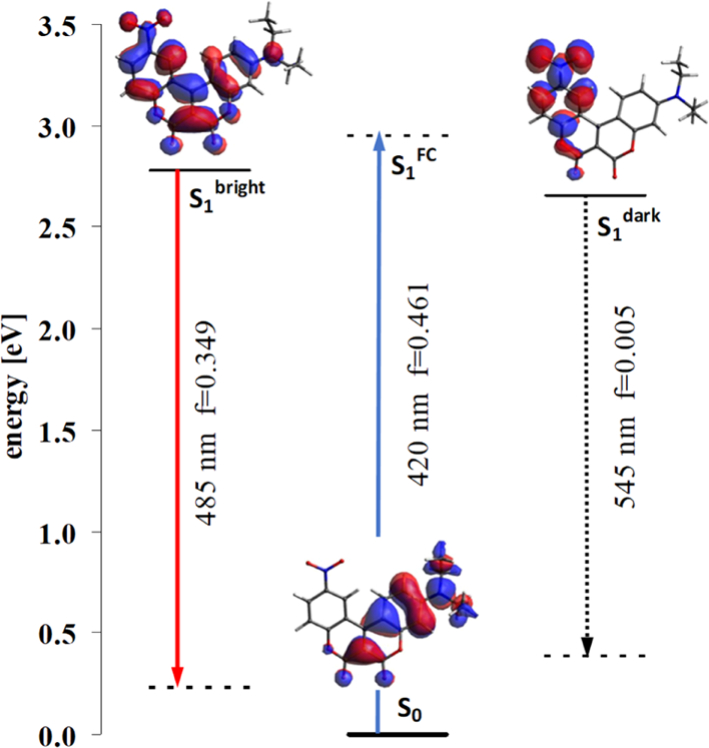
Diagram of electronic states for conjoined biscoumarin **7** (dark and bright forms).

A change in the nature of a functional group, such as the presence of a NO_2_ group (with two low-lying LUMOs), leads to a more complex system of LUMO states in the coumarin core. In energetic proximity to the typical LUMO of the coumarins discussed above (i.e., LUMO delocalized across the whole molecule), a “new” LUMO appears, localized on the coumarin moieties bearing the NO_2_ group (Figure 11). The absorption of conjoined coumarin **7** is analogous to the previously discussed coumarins, but in the excited state the lowest energy state becomes the one described by the “new” LUMO. This is a dark state, meaning the emission from this state is a pure CT transition with negligible oscillator strength.

Nevertheless, testing the potential energy surface in the excited state of coumarin **7** in toluene, another local energy minimum for the excited state was also found (all vibration frequencies were positive). Figure 11 summarizes the energies and oscillator strength of the electronic transitions for coumarin **7**. The energy ordering of states, *E*^FC^ > *E* (bright) > *E* (dark), shows that the energy of the bright state is lower than the excitation energy, that is, it is possible to fill it after excitation. However, the energy of the bright state is higher than the energy of the dark state.

The efficient emission of compound **7** is rare among generally nonfluorescent nitro compounds.^38^ In particular, the counterpart of the compound **7**, V-shaped biscoumarin, in which the 6-nitrocoumarin moiety is replaced by 7-nitrocoumarin scaffold, is also nonfluorescent.^23^ The nitrocoumarin moieties are responsible for the shape and arrangement of the low-lying LUMOs of the entire V-shaped conjoined biscoumarin. When comparing the shape of the frontier orbitals of both nitrocoumarins (as shown in Figure S9), the similar shape of their LUMOs can be seen. These LUMOs contribute to the formation of the S_1_ state of the dark form of compound **7** and the S_1_ state in its nonfluorescent counterpart (involving 7-nitrocoumarin). It can also be seen that the adjacent LUMO + 1 orbital, responsible for the formation of the bright form of compound **7**, is a characteristic feature only of the 6-nitrocoumarin core, absent in the case of 7-nitrocoumarin. Thus, in the differentiation of the properties of compound **7** and the regioisomeric V-shaped conjoined biscoumarin, we have an example of the wider problem of the strong differentiation of properties of coumarins with substituents in the 6 and 7 positions, which already has its own literature.^6,46^

**Figure 12:**
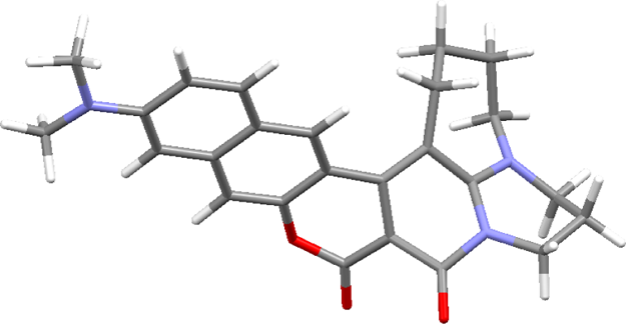
Deformed structure of compound **18** in the excited state [*E*(S_1_ → S_0_) = 0.9640 eV and *f* = 0.0009].

The two V-shaped coumarins **18** and **19** differ by way of rigidification of the bridging nitrogen atom but the results of calculating the electronic structure in the ground state does not show any major differences between them. This is confirmed by the experimental absorption and emission energies. However, while Φ_F_ of dye **19** is large, the fluorescence of **18** is negligible. An analogous observation was made earlier for a number of coumarins with a similar structure.^24^ The reason was the formation of a dark form with deformed geometry in the excited state (Figure 12). As a result of this deformation, the electronic transition becomes a pure intermolecular CT transition with practically zero oscillator strength.

## Conclusions

We have shown that it is possible to extend a previously developed methodology to prepare conjoined V-shaped coumarins from benzo[*g*]coumarins and benzo[*f*]coumarins. All molecules are highly polarized in the ground and excited electronic states. The HOMOs of the conjoined coumarins are localized on a single coumarin subunit (the one that exhibits the larger ionization potential). In contrast, the LUMOs are a sum of the LUMOs of both subunits and are therefore delocalized over the entire molecule. As a result of this, the direction of the dipole moment changes upon excitation. The two lowest excited electronic states S_1_ and S_2_, which are a result of the HOMO → LUMO and HOMO – 1 → LUMO transitions, exhibit a partial CT character as the charge transfer takes place from one of the subunits to the entire molecule. Most of the novel coumarins studied are strongly fluorescent in all solvents; however, placing one amino group at position 6, as in coumarin **8**, changes the photophysics entirely. The significant Coulomb interaction-driven stabilization originating from the larger charge separation in the S_1_ excited state of this coumarin leads to lower energy of the S_1_ → S_0_ transition and a significant drop in oscillator strength. Introducing only moderate charge transfer character into a conjoined biscoumarin possessing an NO_2_ group is a viable strategy to induce strong fluorescence in nonpolar solvents. The results clearly demonstrate that unrestricted dimethyl- or diethylamino groups are better electron donors than their restricted counterpart. A similar effect is observed while comparing 7-substituted versus 6-substituted V-shaped conjoined biscoumarins. These conclusions are valid for the ground state as well as for the excited electronic state. The V-shaped biscoumarins are highly polarized and highly luminescent in contrast with previously studied amide-bridged biscoumarins of (intended) head-to-tail alignment, which, due to their flexible linker, showed a tendency to bend^37^ or even curl themselves.^43^ Apparently, the rigid structure of conjoined coumarin assures concurrently the high dipole moment and efficient fluorescence. Our findings demonstrate that these molecules constitute a unique π-system in which large changes in the dipole moments between ground and excited states, combined with a substantial change of dipole direction, lead to appreciable photophysical properties.

## Experimental Section

### General Information

All reported NMR spectra (^1^H NMR and ^13^C NMR) were recorded using Varian 500 and 600 or Bruker 500 spectrometers. Chemical shifts (δ ppm) were determined with TMS as the internal reference, and *J* values are given in Hz. High-resolution mass spectra (HRMS) were obtained via an electron ionization (EI) source and a EBE double focusing geometry mass analyzer or spectrometer equipped with an electrospray ion source with a q-TOF type mass analyzer. Chromatography was performed on silica gel 60 (230–400 mesh), and thin layer chromatography (TLC) was performed on TLC plates (Merck, silica gel 60 F_254_). Yields of conjoined biscoumarins were always calculated based on the amount of aminophenol used.

### Photophysical Measurements

Room-temperature measurements were performed with dilute solutions in standard cuvettes (10 × 10 mm). Absorption spectra at room temperature (21 °C) were recorded using a PerkinElmer LAMBDA 35 spectrophotometer. Emission spectra were obtained using a FLS 1000 of Edinburgh Instruments spectrofluorometer. Fluorescence kinetics studies were performed using the time-correlated single-photon counting technique.^44^ A mode-locked Coherent Mira-HP picosecond laser pumped by a Verdi 18 laser was used for excitation. The fundamental pulses of the Mira laser (tunable within 760–800 nm) were upconverted to ∼390 nm. The temporal width of the excitation pulses was ∼280 fs and of the instrument response function was about 40 ps. Fluorescence was dispersed with a 0.25 m Jarrell-Ash monochromator and detected with a HMP-100-07 hybrid detector coupled to an SPC-150 PC module (Becker&Hickl GmbH). Fluorescence decays were analyzed with deconvolution software using a nonlinear least squares procedure with the Marquardt algorithm.^45^ A standard χ^2^ test as well as residual and autocorrelation function plots were used to assess the quality of a fit. The estimated accuracy for the determination of decay time was below 10 ps.

### Synthesis

#### Synthesis of Compound **3**

A round bottom flask was charged with methyl 7-(diethylamino)-2-oxo-2*H*-chromene-3-carboxylate (**1**) (2.75 g, 10.0 mmol), 3-diethylaminophenol (**2**) (825 mg, 5.0 mmol), and AlCl_3_ (220 mg, 1.65 mmol). The reaction mixture was stirred (neat) at 140 °C (oil bath) for 24 h. Then, the mixture was cooled to room temperature, dissolved in a small amount of DCM, and purified by column chromatography (silica, DCM/acetone 95:5) to afford a product of analytical purity.

**Compound 3**. Brownish precipitate. Yield: 0.142 g (7%). mp 248–250 °C. ^1^H NMR (CDCl_3_, 500 MHz): δ 8.01 (d, *J* = 9.3 Hz, 2H), 6.63 (dd, *J* = 9.3, 2.6 Hz, 2H), 6.47 (d, *J* = 2.7 Hz, 2H), 3.45 (q, *J* = 7.1 Hz, 8H), 1.24 (t, *J* = 7.1 Hz, 12H). ^13^C{^1^H} NMR (CDCl_3_, 125 MHz): δ 158.0, 157.7, 152.11, 152.07, 130.1, 108.9, 104.5, 97.7, 95.9, 44.9, 12.5. HRMS (ESI) *m*/*z*: calcd for C_24_H_26_N_2_O_4_Na, 429.1790 [M + Na^+^]; found, 429.1781.

#### Synthesis of Compound **5**

A round bottom flask was charged with methyl 7-(diethylamino)-2-oxo-2*H*-chromene-3-carboxylate (**1**) (14.6 g, 53.0 mmol), 8-hydroxyjulolidine (**4**) (5 g, 26.95 mmol), and DMAP (323 mg, 2.65 mmol). The reaction mixture was stirred (neat) at 140 °C (oil bath) for 24 h. Then, the mixture was cooled to room temperature, dissolved in a small amount of DCM, purified by column chromatography (silica, DCM/acetone 95:5), and crystallized from *i*-PrOH-DMF to afford a product of analytical purity.

**Compound 5**. Orange precipitate. Yield: 10.31 g (89%). mp 205 °C (decomp). ^1^H NMR (CDCl_3_, 500 MHz): δ 7.95 (d, *J* = 9.3 Hz, 1H), 7.56 (s, 1H), 6.62 (dd, *J* = 9.5, 2.3 Hz, 1H), 6.45 (d, *J* = 2.3 Hz, 1H), 3.44 (q, *J* = 7.0 Hz, 4H), 3.35 (t, *J* = 5.5 Hz, 2H), 3.31 (t, *J* = 5.5 Hz, 2H), 2.87–2.77 (m, 4H), 2.01 (t, *J* = 5.5 Hz, 2H), 1.95 (t, *J* = 5.5 Hz, 2H), 1.23 (t, *J* = 7.0 Hz, 6H). ^13^C{^1^H} NMR (CDCl_3_, 125 MHz): δ 158.3, 158.2, 157.4, 152.5, 151.9, 151.8, 147.8, 130.3, 126.0, 118.5, 108.7, 106.4, 104.6, 104.1, 97.6, 95.1, 50.1, 49.6, 44.9, 27.7, 21.3, 20.4, 20.3, 12.5. HRMS (ESI) *m*/*z*: calcd for C_26_H_27_N_2_O_4_, 431.1971 [M + H^+^]; found, 431.1966.

#### Synthesis of Compound **7**

A round bottom flask was charged with ethyl 6-nitro-2-oxo-2*H*-chromene-3-carboxylate (**6**) (2.1 g, 8 mmol), 3-diethylaminophenol (**2**) (660 mg, 4 mmol), and In(OTf)_3_ (50 mg, 0.08 mmol). The reaction mixture was stirred (neat) at 140 °C (oil bath) for 5 h. Then, the mixture was cooled to room temperature. The resulting precipitate was crystalized from MeOH to afford a product of analytical purity.

**Compound 7**. Orange precipitate. Yield: 1.27 g (83%). mp 243–244 °C. ^1^H NMR (CDCl_3_, 500 MHz): δ 9.19 (d, *J* = 2.4 Hz, 1H), 8.51 (dd, *J* = 9.1, 2.5 Hz, 1H), 8.05 (d, *J* = 9.5 Hz, 1H), 7.54 (d, *J* = 9.1 Hz, 1H), 6.83 (dd, *J* = 9.4, 2.6 Hz, 1H), 6.59 (d, *J* = 2.6 Hz, 1H), 3.54 (q, *J* = 7.2 Hz, 4H), 1.31 (t, *J* = 7.2 Hz, 6H). ^13^C{^1^H} NMR (CDCl_3_, 125 MHz): δ 158.5, 156.3, 155.1, 153.4, 150.4, 143.7, 129.3, 128.3, 124.8, 119.3, 116.3, 110.8, 103.6, 100.1, 98.0, 45.3, 12.5. HRMS (EI): *m*/*z*: calcd for C_20_H_16_N_2_O_6_, 380.1008 [M^•+^]; found, 380.0999.

#### Synthesis of Compound **8**

Tin dichloride dihydrate (678 mg, 3 mmol) was added to the solution of compound **7** (285 mg, 0.75 mmol) in ethanol (50 ml). The reaction mixture was refluxed (oil bath) for 5 h. Then, the mixture was cooled to room temperature and aqueous NaHCO_3_ was added until the pH became neutral. The aqueous solution was extracted with DCM, and the combined organic layers were dried over Na_2_SO_4_ and concentrated under reduced pressure. The crude product was purified by column chromatography (silica, DCM/acetone 4:1) and crystallized from DCM-Et_2_O affording a product of analytical purity.

**Compound 8**. Red precipitate. Yield: 0.142 g (54%). mp 133–135 °C. ^1^H NMR (CD_3_CN, 500 MHz): δ 8.19 (d, *J* = 9.4 Hz, 1H), 7.50 (d, *J* = 2.5 Hz, 1H), 7.14 (d, *J* = 8.8 Hz, 1H), 7.04 (dd, *J* = 8.8, 2.6 Hz, 1H), 6.77 (dd, *J* = 9.4, 2.7 Hz, 1H), 6.56 (d, *J* = 2.7 Hz, 1H), 4.35 (br s, 2H), 3.51 (q, *J* = 7.1 Hz, 4H), 1.21 (t, *J* = 7.1 Hz, 6H). ^13^C{^1^H} NMR (CDCl_3_, 125 MHz): δ 157.9, 157.4, 157.3, 152.6, 152.1, 147.9, 143.1, 130.1, 122.2, 118.6, 116.3, 112.4, 109.6, 104.2, 100.3, 97.5, 45.1, 12.5. HRMS (EI): *m*/*z*: calcd for C_20_H_18_N_2_O_4_, 350.1267 [M^•+^]; found, 350.1280.

#### General Procedure for the Synthesis of Compounds **10–13**

A round bottom flask was charged with ethyl 8-(dimethylamino)-2-oxo-2*H*-benzo[*g*]chromene-3-carboxylate (**9**) (156 mg, 0.5 mmol), appropriate phenol (3-diethylaminophenol (**2**) (41 mg, 0.25 mmol), 8-hydroxyjulolidine (**4**) (47 mg, 0.25 mmol), 3-ethylamino-*p*-cresol (38 mg, 0.25 mmol) or 7-hydroxy-1,2,3,4-tetrahydroquinoline (37 mg, 0.25 mmol)), and DMAP (0.6 mg, 0.005 mmol). The reaction mixture was stirred (neat) at 140 °C (oil bath) for 5 h. Then the mixture was cooled to room temperature, dissolved in a small amount of DCM, and purified by column chromatography (silica, DCM/MeOH 98:2). Crystallization from MeOH afforded a product of analytical purity.

**Compound 10**. Brown precipitate. Yield: 0.036 g (34%). mp 250 °C (decomp). ^1^H NMR (CD_2_Cl_2_, 500 MHz): δ 8.51 (s, 1H), 8.16 (d, *J* = 9.4 Hz, 1H), 7.80 (d, *J* = 9.2 Hz, 1H), 7.37 (s, 1H), 7.15 (dd, *J* = 9.2, 2.5 Hz, 1H), 6.78–6.73 (m, 2H), 6.53 (d, *J* = 2.7 Hz, 1H), 3.51 (q, *J* = 7.1 Hz, 4H), 3.13 (s, 6H), 1.28 (t, *J* = 7.1 Hz, 6H). ^13^C{^1^H} NMR (CD_2_Cl_2_, 125 MHz): δ 158.5, 157.5, 157.4, 153.05, 153.03, 151.9, 151.3, 138.5, 131.0, 130.9, 130.8, 123.6, 116.4, 112.3, 110.4, 110.0, 104.9, 103.4, 98.9, 97.9, 45.4, 40.4, 12.7. HRMS (EI): *m*/*z*: calcd for C_26_H_24_N_2_O_4_, 428.1736 [M^•+^]; found, 428.1748.

**Compound 11**. Red precipitate. Yield: 0.044 g (39%). mp 230 °C (decomp). ^1^H NMR (CD_2_Cl_2_, 500 MHz): δ 8.54 (s, 1H), 7.83 (d, *J* = 9.2 Hz, 1H), 7.78 (s, 1H), 7.43 (s, 1H), 7.18 (d, *J* = 9.3 Hz, 1H), 6.83 (s, 1H), 3.42–3.36 (m, 4H), 3.15 (s, 6H), 2.92–2.84 (m, 4H), 2.07–1.99 (m, 4H). ^13^C{^1^H} NMR (CD_2_Cl_2_, 125 MHz): δ 155.54, 155.47, 153.87, 153.47, 153.44, 151.4, 140.6, 133.5, 133.3, 128.9, 125.8, 122.4, 118.6, 114.7, 112.6, 110.0, 109.0, 106.9, 52.8, 52.3, 42.6, 30.4, 23.8, 22.8. HRMS (EI): *m*/*z*: calcd for C_28_H_24_N_2_O_4_: 452.1736 [M^•+^]; found, 452.1729.

**Compound 12**. Dark red precipitate. Yield: 0.037 g (36%). mp 296 °C (decomp). ^1^H NMR (CD_2_Cl_2_, 500 MHz): δ 8.58 (s, 1H), 8.02 (s, 1H), 7.85 (d, *J* = 9.2 Hz, 1H), 7.44 (s, 1H), 7.20 (dd, *J* = 9.2, 2.3 Hz, 1H), 6.86 (s, 1H), 6.52 (s, 1H), 4.51 (br s, 1H), 3.36 (q, *J* = 7.2 Hz, 2H), 3.15 (s, 6H), 2.27 (s, 3H), 1.38 (t, *J* = 7.2 Hz, 3H). ^13^C{^1^H} NMR (CD_2_Cl_2_, 125 MHz): δ 157.2, 157.1, 157.0, 152.9, 152.2, 151.4, 150.7, 138.0, 130.7, 130.6, 129.2, 123.2, 119.6, 116.0, 112.1, 110.1, 105.0, 103.4, 99.3, 96.0, 40.2, 38.3, 17.1, 14.1. HRMS (EI): *m*/*z*: calcd for C_25_H_22_N_2_O_4_, 414.1580 [M^•+^]; found, 414.1582.

**Compound 13**. Brown precipitate. Yield: 0.042 g (41%). mp 248 °C (decomp). ^1^H NMR (DMSO-*d*_6_, 500 MHz): δ 8.72 (s, 1H), 8.06 (d, *J* = 9.3 Hz, 1H), 7.94 (s, 1H), 7.68 (br s, 1H), 7.47 (s, 1H), 7.27 (dd, *J* = 9.3, 2.5 Hz, 1H), 6.91 (d, *J* = 2.1 Hz, 1H), 6.38 (s, 1H), 3.38–3.32 (m, 2H, overlap by signal from DMSO), 3.12 (s, 6H), 2.87 (t, *J* = 5.9 Hz, 2H), 1.91–1.83 (m, 2H). ^13^C{^1^H} NMR (DMSO-*d*_6_, 125 MHz): δ 156.4, 156.2, 155.9, 152.0, 151.9, 150.8, 150.7, 137.6, 131.3, 130.9, 129.3, 122.8, 119.2, 115.9, 111.2, 109.3, 103.7, 102.7, 97.5, 96.6, 40.5 (overlap by signal from DMSO), 40.1 (overlap by signal from DMSO), 26.3, 20.5. HRMS (ESI) *m*/*z*: calcd for C_25_H_20_N_2_O_4_Na, 435.1321 [M + Na^+^]; found, 435.1322.

#### General Procedure for the Synthesis of Compounds **15–17**

A round bottom flask was charged with ethyl 3-oxo-3*H*-benzo[*f*]chromene-2-carboxylate (**14**) (402 mg, 1.5 mmol), appropriate phenol (8-hydroxyjulolidine (**4**) (142 mg, 0.75 mmol), 3-ethylamino-*p*-cresol (113 mg, 0.75 mmol) or 7-hydroxy-1,2,3,4-tetrahydroquinoline (112 mg, 0.75 mmol)), and DMAP (2 mg, 0.015 mmol). The reaction mixture was stirred (neat) at 140 °C (oil bath) for 5 h. The mixture was then cooled to room temperature, dissolved in a small amount of DCM, and purified by column chromatography (silica, DCM/acetone 9:1). Crystallization from MeOH afforded a product of analytical purity.

**Compound 15**. Red precipitate. Yield: 0.096 g (31%). mp 159 °C (decomp). ^1^H NMR (CDCl_3_, 600 MHz): δ 8.13 (d, *J* = 8.6 Hz, 1H), 8.04 (d, *J* = 8.9 Hz, 1H), 7.87 (d, *J* = 7.9 Hz, 1H), 7.49 (ddd, *J* = 8.0, 6.9, 1.0 Hz, 1H), 7.44 (d, *J* = 8.9 Hz, 1H), 7.38 (ddd, *J* = 8.3, 6.9, 1.3 Hz, 1H), 7.10 (s, 1H), 3.42–3.32 (m, 4H), 3.02–2.90 (m, 2H), 2.60–2.54 (m, 1H), 2.50–2.44 (m, 1H), 2.11–1.83 (m, 4H). ^13^C{^1^H} NMR (CDCl_3_, 125 MHz): δ 157.6, 157.3, 154.7, 153.1, 152.8, 148.8, 135.1, 131.0, 129.9, 128.5, 127.6, 126.7, 126.1, 125.8, 117.8, 117.3, 110.6, 106.1, 105.1, 99.7, 50.2, 49.9, 27.3, 21.2, 20.31, 20.27. HRMS (EI): *m*/*z*: calcd for C_26_H_19_NO_4_, 409.1314 [M^•+^]; found, 409.1319.

**Compound 16**. Yellow precipitate. Yield: 0.049 g (18%). mp 304 °C (decomp). ^1^H NMR (DMSO-*d*_6_, 500 MHz): δ 8.29 (d, *J* = 9.0 Hz, 1H), 8.04 (d, *J* = 7.9 Hz, 1H), 8.02 (d, *J* = 8.7 Hz, 1H), 7.58–7.52 (m, 2H), 7.47 (ddd, *J* = 8.4, 7.0, 1.3 Hz, 1H), 7.14 (s, 1H), 6.71 (t, *J* = 5.5 Hz, 1H), 6.51 (s, 1H), 3.31 (q, *J* = 7.1 Hz, 2H, overlap by signal from DMSO), 1.89 (s, 3H), 1.22 (t, *J* = 7.1 Hz, 3H). ^13^C{^1^H} NMR (DMSO-*d*_6_, 125 MHz): δ 157.3, 156.7, 156.4, 154.6, 153.8, 153.2, 136.2, 131.1, 130.5, 129.7, 129.1, 126.9, 126.8, 126.4, 119.4, 117.3, 110.3, 104.8, 99.9, 94.9, 37.9, 17.6, 14.4. HRMS (EI): *m*/*z*: calcd for C_23_H_17_NO_4_, 371.1158 [M^•+^]; found, 371.1154.

**Compound 17**. Orange precipitate. Yield: 0.051 g (18%). mp 280 °C (decomp). ^1^H NMR (CDCl_3_, 600 MHz): δ 8.15 (d, *J* = 8.4 Hz, 1H), 8.07 (d, *J* = 8.9 Hz, 1H), 7.90 (d, *J* = 8.0 Hz, 1H), 7.52 (ddd, *J* = 7.9, 7.1, 0.8 Hz, 1H), 7.46 (d, *J* = 8.9 Hz, 1H), 7.43 (ddd, *J* = 8.4, 7.2, 1.1 Hz, 1H), 7.28 (s, 1H), 6.51 (s, 1H), 5.39 (s, 1H), 3.48–3.44 (m, 2H), 2.65–2.59 (m, 1H), 2.53–2.47 (m, 1H), 2.02–1.94 (m, 1H), 1.90–183 (m, 1H). ^13^C{^1^H} NMR (CDCl_3_, 125 MHz): δ 157.5, 157.1, 156.3, 154.9, 153.6, 151.7, 135.5, 131.0, 130.5, 129.8, 128.6, 126.6, 126.3, 126.0, 118.0, 117.3, 110.4, 105.9, 100.8, 98.6, 41.7, 26.5, 21.0. HRMS (EI): *m*/*z*: calcd for C_23_H_15_NO_4_, 369.1001 [M^•+^]; found, 369.1007.

#### General Procedure for the Synthesis of Compounds **18** and **19**

A round bottom flask was charged with ethyl 8-(dimethylamino)-2-oxo-2*H*-benzo[*g*]chromene-3-carboxylate (**9**) (415 mg, 1.5 mmol) and DBU (225 μL, 0.75 mmol) or DBN (93 μL, 0.75 mmol). The reaction mixture was stirred (neat) at 120 °C (oil bath) in an open flask for 2 h in the case of DBU or 30 min in the case of DBN. Then, the mixture was cooled to room temperature. The resulting precipitate was crystalized from EtOH to afford a product of analytical purity.

**Compound 18**. Yellow precipitate. Yield: 0.185 g (59%). mp 250 °C (decomp). ^1^H NMR (CDCl_3_, 500 MHz): δ 8.19 (s, 1H), 7.66 (d, *J* = 9.0 Hz, 1H), 7.35 (s, 1H), 7.06 (d, *J* = 9.0 Hz, 1H), 6.75 (s, 1H), 4.21 (br s, 2H), 3.71 (br s, 2H), 3.40 (t, *J* = 6.3 Hz, 2H), 3.10 (s, 8H), 2.13–2.00 (m, 4H), 1.97–1.89 (m, 2H). ^13^C{^1^H} NMR (CDCl_3_, 125 MHz): δ 158.9, 158.5, 155.0, 151.2, 151.1, 150.0, 136.8, 129.9, 129.7, 122.5, 115.4, 114.5, 110.1, 103.7, 98.0, 97.9, 53.4, 49.3, 40.4, 38.8, 31.6, 25.0, 24.9, 22.8. HRMS (ESI) *m*/*z*: calcd for C_25_H_25_N_3_O_3_Na, 438.1794 [M + Na^+^]; found, 438.1788.

**Compound 19**. Yellow precipitate. Yield: 0.212 g (73%). mp 310 °C (decomp). ^1^H NMR (CD_2_Cl_2_, 500 MHz): δ 7.84 (s, 1H), 7.49 (d, *J* = 9.4 Hz, 1H), 7.21 (s, 1H), 6.97 (dd, *J* = 9.0, 1.9 Hz, 1H), 6.67 (d, *J* = 2.0 Hz, 1H), 3.89–3.80 (m, 4H), 3.37–3.33 (m, 2H), 3.32–3.25 (m, 2H), 3.08 (s, 6H), 2.18–2.09 (m, 2H). ^13^C{^1^H} NMR (CD_2_Cl_2_, 125 MHz): δ 159.6, 155.5, 150.8, 149.9, 141.6, 136.5, 129.7, 127.3, 122.8, 115.2, 113.9, 110.0, 109.4, 103.1, 96.5, 90.1, 51.6, 42.0, 40.1, 37.5, 27.4, 19.5. HRMS (EI): *m*/*z*: calcd for C_23_H_21_N_3_O_3_, 387.1583 [M^•+^]; found, 387.1592.

#### Synthesis of Compound **20**

A round bottom flask was charged with ethyl 3-oxo-3*H*-benzo[*f*]chromene-2-carboxylate (**14**) (402 mg, 1.5 mmol) and DBN (93 μL, 0.75 mmol). The reaction mixture was stirred (neat) at 120 °C (oil bath) in an open flask for 30 min. The mixture was then cooled to room temperature, dissolved in a small amount of DCM, and purified by column chromatography (silica, DCM/MeOH 95:5). Crystallization from MeOH afforded a product of analytical purity.

**Compound 20**. Yellow precipitate. Yield: 0.097 g (38%). mp 196–198 °C. ^1^H NMR (CDCl_3_, 500 MHz): δ 7.89–7.84 (m, 2H), 7.66 (d, *J* = 8.3 Hz, 1H), 7.51 (ddd, *J* = 8.2, 7.0, 1.3 Hz, 1H), 7.45 (ddd, *J* = 8.0, 6.9, 1.1 Hz, 1H), 7.27 (d, *J* = 7.9 Hz, 1H), 4.08 (t, *J* = 5.9 Hz, 2H), 3.75 (br s, 2H), 3.43 (t, *J* = 5.6 Hz, 2H), 2.23 (quintet, *J* = 5.9 Hz, 2H), 1.69 (s, 2H). ^13^C{^1^H} NMR (CDCl_3_, 125 MHz): δ 159.22, 159.18, 156.5, 153.3, 143.8, 132.9, 130.7, 128.6, 128.57, 126.6, 126.1, 124.9, 117.4, 111.5, 98.1, 93.3, 52.1, 42.6, 38.0, 28.7, 20.1. HRMS (ESI) *m*/*z*: calcd for C_21_H_16_N_2_O_3_Na, 367.1059 [M + Na^+^]; found, 367.1059.

#### Formation of Spiro-coumarin **21**

Compound **19** (120 mg, 0.31 mmol) was dissolved in DCM (500 ml) and the reaction mixture was exposed to the air and sunlight for 2 weeks. After this time, the solvent was evaporated and the crude product was purified by column chromatography (silica, DCM/MeOH 95:5) and crystallized from MeOH affording a product of analytical purity.

#### Spiro-coumarin **21**

Red precipitate. Yield: 0.023 g (18%). mp 235 °C (decomp). ^1^H NMR (CDCl_3_, 500 MHz): δ 7.74 (d, *J* = 9.2 Hz, 1H), 7.68 (s, 1H), 7.48 (s, 1H), 7.14 (dd, *J* = 9.2, 2.4 Hz, 1H), 6.79 (d, *J* = 2.5 Hz, 1H), 3.82–3.75 (m, 1H), 3.65 (t, *J* = 8.6 Hz, 1H), 3.54–3.50 (m, 1H), 3.46–3.39 (m, 2H), 3.36–3.28 (m, 1H), 3.17 (s, 6H), 2.88–2.79 (m, 1H), 2.53–2.46 (m, 1H), 1.92–1.86 (m, 2H). ^13^C{^1^H} NMR (CD_2_Cl_2_, 150 MHz): δ 168.1, 165.2, 156.8, 155.0, 152.6, 151.2, 139.2, 130.7, 126.4, 123.1, 116.5, 110.5, 109.4, 109.2, 103.5, 87.9, 48.7 (overlap by signal from MeOH), 43.7, 43.6, 40.0, 33.4, 19.8. HRMS (EI): *m*/*z*: calcd for C_23_H_21_N_3_O_4_, 403.1532 [M^•+^]; found, 403.1518.

## Supporting Information Available

Synthesis details, X-ray crystallographic information; photophysical data; theoretical data; copies of ^1^H and ^13^C{^1^H} NMR spectra; and X-ray crystal data for **21** (PDF)
Cartesian coordinates related to computational studies (PDF)

## Accession Codes

CCDC 2144352 contains the supplementary crystallographic data for this paper. These data can be obtained free of charge via www.ccdc.cam.ac.uk/data_request/cif, or by emailing data_request@ccdc.cam.ac.uk, or by contacting The Cambridge Crystallographic Data Centre, 12 Union Road, Cambridge CB2 1EZ, UK; fax: +44 1223 336033.

## References

[ref1] von Pechmann H. II; Welsh W. Ueber Einige Neue Cumarine. Ber. Dtsch. Chem. Ges. 1884, 17, 1646–1652. 10.1002/cber.18840170222.

[ref2] aSoine T. O. Naturally Occurring Coumarins and Related Physiological Activities. J. Pharm. Sci. 1964, 53, 231–264. 10.1002/jps.2600530302. 14191429

[ref3] Sardari S.; Mori Y.; Horita K.; Micetich R. G.; Nishibe S.; Daneshtalab M. Synthesis and Antifungal Activity of Coumarins and Angular Furanocoumarins. Bioorg. Med. Chem. 1999, 7, 1933–1940. 10.1016/s0968-0896(99)00138-8. 10530942

[ref4] García-Argáez A.; Ramírez Apan T.; Delgado H.; Velázquez G.; Martínez-Vázquez M. Anti-Inflammatory Activity of Coumarins from Decatropis Bicolor on TPA Ear Mice Model. Planta Med. 2000, 66, 279–281. 10.1055/s-2000-14894. 10821059

[ref5] aSchiedel M.-S.; Briehn C. A.; Bäuerle P. Single-Compound Libraries of Organic Materials: Parallel Synthesis and Screening of Fluorescent Dyes. Angew. Chem., Int. Ed. 2001, 40, 4677–4680. 10.1002/1521-3773(20011217)40:24<4677::aid-anie4677>3.0.co;2-u. 12404382

[ref6] aXie L.; Chen Y.; Wu W.; Guo H.; Zhao J.; Yu X. Fluorescent Coumarin Derivatives with Large Stokes Shift, Dual Emission and Solid State Luminescent Properties: An Experimental and Theoretical Study. Dyes Pigm. 2012, 92, 1361–1369. 10.1016/j.dyepig.2011.09.023.

[ref7] aSun X.-Y.; Liu T.; Sun J.; Wang X.-J. Synthesis and Application of Coumarin Fluorescence Probes. RSC Adv. 2020, 10, 10826–10847. 10.1039/c9ra10290f. 35492912 PMC9050418

[ref8] Mishra A.; Fischer M. K. R.; Bäuerle P. Metal-Free Organic Dyes for Dye-Sensitized Solar Cells: From Structure: Property Relationships to Design Rules. Angew. Chem., Int. Ed. 2009, 48, 2474–2499. 10.1002/anie.200804709. 19294671

[ref9] aZhang H.; Yu T.; Zhao Y.; Fan D.; Xia Y.; Zhang P. Synthesis, Crystal Structure, Photo- and Electro-Luminescence of 3-(4-(Anthracen-10-yl)Phenyl)-7-(N,N,’-Diethylamino)Coumarin. Synth. Met. 2010, 160, 1642–1647. 10.1016/j.synthmet.2010.05.034.

[ref10] aRaikar U. S.; Tangod V. B.; Mannopantar S. R.; Mastiholi B. M. Ground and Excited State Dipole Moments of Coumarin 337 Laser Dye. Opt. Commun. 2010, 283, 4289–4292. 10.1016/j.optcom.2010.06.037.

[ref11] aNiu G.; Liu W.; Xiao H.; Zhang H.; Chen J.; Dai Q.; Ge J.; Wu J.; Wang P. Keto-Benzo[h]-Coumarin-Based Near-Infrared Dyes with Large Stokes Shifts for Bioimaging Applications. Chem.—Asian J. 2016, 11, 498–504. 10.1002/asia.201501026. 26558738

[ref12] aKim D.; Singha S.; Wang T.; Seo E.; Lee J. H.; Lee S.-J.; Kim K. H.; Ahn K. H. In Vivo Two-Photon Fluorescent Imaging of Fluoride with a Desilylation-Based Reactive Probe. Chem. Commun. 2012, 48, 10243–10245. 10.1039/c2cc35668f. 22968490

[ref13] Węcławski M. K.; Deperasińska I.; Banasiewicz M.; Young D. C.; Leniak A.; Gryko D. T. Building Molecular Complexity from Quinizarin: Conjoined Coumarins and Coronene Analogs. Chem.—Asian J. 2019, 14, 1763–1779. 10.1002/asia.201800757. 30022613

[ref14] aMukhopadhyay A.; Hossen T.; Ghosh I.; Koner A. L.; Nau W. M.; Sahu K.; Moorthy J. N. Helicity-Dependent Regiodifferentiation in the Excited-State Quenching and Chiroptical Properties of Inward/Outward Helical Coumarins. Chem.—Eur. J. 2017, 23, 14797–14805. 10.1002/chem.201701787. 28792106

[ref15] Högberg T.; Vora M.; Drake S. D.; Mitscher L. A.; Chu D. T. W. Structure-Activity Relationships among DNA-Gyrase Inhibitors. Sytnthesis and Antimicrobial Evaluation of Chromones and Coumarins Related to Oxolinic Acid. Acta Chem. Scand., Ser. B 1984, 38b, 359–366. 10.3891/acta.chem.scand.38b-0359. 6091380

[ref16] Poronik E. M.; Shandura M. P.; Kovtun Y. P. Synthesis of 6H,7H-[1]benzopyrano[3,4-c][1]benzopyran-6,7-diones. Chem. Heterocycl. Compd. 2006, 42, 410–411. 10.1007/s10593-006-0102-6.

[ref17] Seijas J. A.; Crecente-Campo J.; Vázquez-Tato M. P. Microwave Assisted Synthesis of Coumarocoumarins. 16th International Electronic Conference on Synthetic Organic Chemistry, 2012.

[ref18] aXi G.-L.; Liu Z.-Q. Coumarin-Fused Coumarin: Antioxidant Story from N,N-Dimethylamino and Hydroxyl Groups. J. Agric. Food Chem. 2015, 63, 3516–3523. 10.1021/acs.jafc.5b00399. 25826201

[ref19] Chen C.; Zhou L.; Liu F.; Li Z.; Liu W.; Liu W. V-Shaped Bis-Coumarin Based Fluorescent Probe for Detecting Palladium in Natural Waters. J. Hazard. Mater. 2020, 386, 121943 10.1016/j.jhazmat.2019.121943. 31884355

[ref20] Jiang Y.; Li H.; Chen R.; Liu W.; Chen C.; Li Z.; Liu W. Novel Fluorescent Probe Based on Dicoumarin for Rapid On-Site Detection of Hg^2+^ in Loess. Spectrochim. Acta, Part A 2021, 251, 119438 10.1016/j.saa.2021.119438. 33461142

[ref21] aJiang X.; Shangguan M.; Lu Z.; Yi S.; Zeng X.; Zhang Y.; Hou L. A “Turn-on” Fluorescent Probe Based on V-Shaped Bis-Coumarin for Detection of Hydrazine. Tetrahedron 2020, 76, 130921 10.1016/j.tet.2020.130921.

[ref22] aChen C.; Zhou L.; Liu W.; Liu W. Coumarinocoumarin-Based Two-Photon Fluorescent Cysteine Biosensor for Targeting Lysosome. Anal. Chem. 2018, 90, 6138–6143. 10.1021/acs.analchem.8b00434. 29687719

[ref23] Tasior M.; Poronik Y. M.; Vakuliuk O.; Sadowski B.; Karczewski M.; Gryko D. T. V-Shaped Bis-Coumarins: Synthesis and Optical Properties. J. Org. Chem. 2014, 79, 8723–8732. 10.1021/jo501565r. 25133521

[ref24] Poronik Y. M.; Gryko D. T. Pentacyclic Coumarin-Based Blue Emitters – the Case of Bifunctional Nucleophilic Behavior of Amidines. Chem. Commun. 2014, 50, 5688–5690. 10.1039/c4cc01106f. 24652376

[ref25] Lippert E.; Lüder W.; Moll F.; Nägele W.; Boos H.; Prigge H.; Seibold-Blankenstein I. Umwandlung von Elektronenanregungsenergie. Angew. Chem. 1961, 73, 695–706. 10.1002/ange.19610732103.

[ref26] Rotkiewicz K.; Grellmann K. H.; Grabowski Z. R. Rinterpretation of the Anomalous Fluorescence of p-N,N-Dimethylaminobenzonitrile. Chem. Phys. Lett. 1973, 19, 315–318. 10.1016/0009-2614(73)80367-7.

[ref27] Haberhauer G. Planarized and Twisted Intramolecular Charge Transfer: A Concept for Fluorophores Showing Two Independent rotations in Excited State. Chem.—Eur. J. 2017, 23, 9288–9296. 10.1002/chem.201700566. 28370593

[ref28] aLakowicz J. R. Principles of Fluorescence Spectroscopy; Springer: Heidelberg, 2006; p 213.

[ref29] aZachariasse K. A.; Grobys M.; Von der Haar T.; Hebecker A.; Il’ichev Y. V.; Jiang Y.-B.; Morawski O.; Kühnle W. Intramolecular Charge Transfer in the Excited State. Kinetics and Configurational Changes. J. Photochem. Photobiol., A 1996, 102, 59–70. 10.1016/s1010-6030(96)04368-7.

[ref30] Kielesiński Ł.; Morawski O. W.; Barboza C. A.; Gryko D. T. Polarized Helical Coumarins: [1,5] Sigmatropic Rearrangement and Excited-State Intramolecular Proton Transfer. J. Org. Chem. 2021, 86, 6148–6159. 10.1021/acs.joc.0c02978. 33830755 PMC8154611

[ref31] aYoshida S.; Nakamura Y.; Uchida K.; Hazama Y.; Hosoya T. Aryne Relay Chemistry en Route to Aminoarenes: Synthesis of 3-Aminoaryne Precursors via Regioselective Silylamination of 3-(Triflyoxy)arynes. Org. Lett. 2016, 18, 6212–6215. 10.1021/acs.orglett.6b03304. 27934384

[ref32] Kielesiński Ł.; Gryko D. T.; Sobolewski A. L.; Morawski O. W. Effect of Conformational Flexibility on Photophysics of Bis-Comuarins. Phys. Chem. Chem. Phys. 2018, 20, 14491–14503. 10.1039/c8cp01084f. 29766172

[ref33] Kielesiński Ł.; Gryko D. T.; Sobolewski A. L.; Morawski O. The Interplay between Solvation and Stacking of Aromatic Rings Governs Bright and Dark Sites of Benzo[g]coumarins. Chem.—Eur. J. 2019, 25, 15305–15314. 10.1002/chem.201903018. 31523856

[ref34] aKim I.; Kim D.; Sambasivan S.; Ahn K. H. Synthesis of π-Extended Coumarins and Evaluation of Their Precursors as Reactive Fluorescent Probes for Mercury Ions. Asian J. Org. Chem. 2012, 1, 60–64. 10.1002/ajoc.201200034.

[ref35] Ventura B.; Poronik Y. M.; Deperasińska I.; Gryko D. T. How a Small Structural Difference Can Turn Optical Properties of π-Extended Coumarins Upside Down: The Role of Non-Innocent Saturated Rings. Chem.—Eur. J. 2016, 22, 15380–15388. 10.1002/chem.201603038. 27619245

[ref36] Singha S.; Kim D.; Roy B.; Sambasivan S.; Moon H.; Rao A. S.; Kim J. Y.; Joo T.; Park J. W.; Rhee Y. M.; Wang T.; Kim K. H.; Shin Y. H.; Jung J.; Ahn K. H. A Structural Remedy Toward Bright Dipolar Fluorophores in Aqueous Media. Chem. Sci. 2015, 6, 4335–4342. 10.1039/c5sc01076d. 29218204 PMC5707477

[ref37] Kielesiński Ł.; Morawski O.; Dobrzycki Ł.; Sobolewski A. L.; Gryko D. TD. T. The Coumarin-Dimer Spring – The Struggle Between Charge Transfer and Steric Interactions. Chem.—Eur. J. 2017, 23, 9174–9184. 10.1002/chem.201701387. 28500858

[ref38] aChen M. C.; Chen D. G.; Chou P. T. Fluorescent Chromophores Containing the Nitro Group: Relatively Unexplored Emissive Properties. ChemPlusChem 2020, 86, 11–27. 10.1002/cplu.202000592. 33094565

[ref39] Frisch M. J.; Trucks G. W.; Schlegel H. B.; Scuseria G. E.; Robb M. A.; Cheeseman J. R.; Scalmani G.; Barone V.; Petersson G. A.; Nakatsuji H.; Li X.; Caricato M.; Marenich A. V.; Bloino J.; Janesko B. G.; Gomperts R.; Mennucci B.; Hratchian H. P.; Ortiz J. V.; Izmaylov A. F.; Sonnenberg J. L.; Williams-Young D.; Ding F.; Lipparini F.; Egidi F.; Goings J.; Peng B.; Petrone A.; Henderson T.; Ranasinghe D.; Zakrzewski V. G.; Gao J.; Rega N.; Zheng G.; Liang W.; Hada M.; Ehara M.; Toyota K.; Fukuda R.; Hasegawa J.; Ishida M.; Nakajima T.; Honda Y.; Kitao O.; Nakai H.; Vreven T.; Throssell K.; Montgomery J. A. Jr.; Peralta J. E.; Ogliaro F.; Bearpark M. J.; Heyd J. J.; Brothers E. N.; Kudin K. N.; Staroverov V. N.; Keith T. A.; Kobayashi R.; Normand J.; Raghavachari K.; Rendell A. P.; Burant J. C.; Iyengar S. S.; Tomasi J.; Cossi M.; Millam J. M.; Klene M.; Adamo C.; Cammi R.; Ochterski J. W.; Martin R. L.; Morokuma K.; Farkas O.; Foresman J. B.; Fox D. J. Gaussian 16, Revision A.03. Gaussian, Inc.: Wallingford CT, 2016.

[ref40] aBudzák Š.; Charaf-Eddin A.; Medved’ M.; Gryko D. T.; Jacquemin D. Optical Properties of V-Shaped Bis-Coumarins: Ab Initio Insights. Comput. Theor. Chem. 2016, 1076, 57–64. 10.1016/j.comptc.2015.12.001.

[ref41] Samanta A.; Fessenden R. W. Excited-State Dipole Moment of 7-Aminocoumarins as Determined from Time-Resolved Microwave Dielectric Absorption Measurements. J. Phys. Chem. A 2000, 104, 8577–8582. 10.1021/jp001676j.

[ref42] Kapturkiewicz A.; Herbich J.; Karpiuk J.; Nowacki J. Intramolecular Radiative and Radiationless Charge Recombination Processes in Donor-Acceptor Carbazole Derivatives. J. Phys. Chem. A 1997, 101, 2332–2344. 10.1021/jp9634565.

[ref43] Kielesiński Ł.; Morawski O. W.; Sobolewski A. L.; Gryko D. T. The Synthesis and Photophysical Properties of Tris-Coumarins. Phys. Chem. Chem. Phys. 2019, 21, 8314–8325. 10.1039/c9cp00978g. 30951072

[ref44] Birks J. B. Photophysics of Aromatic Molecules; Wiley: London, 1979; pp 97–100.

[ref45] Demas J. N. Excited State Lifetime Measurements; Academic Press: NY, 1983; pp 89–92.

[ref46] Górski K.; Deperasińska I.; Baryshnikov G. V.; Ozaki S.; Kamada K.; Ågren H.; Gryko D. T. Quadrupolar Dyes Based on Highly Polarized Coumarins. Org. Lett. 2021, 23, 6770–6774. 10.1021/acs.orglett.1c02349. 34474569 PMC8419859

