# Supplementary material for: Polarized, V-Shaped, and Conjoined Biscoumarins: From Lack of Dipole Moment Alignment to High Brightness
Source: J Org Chem. 2022 Apr 12;87(9):5961–75. doi: 10.1021/acs.joc.2c00232 (PMC9087199; doi:10.1021/acs.joc.2c00232)
Supplement: Supplementary file 1 — jo2c00232_si_001.pdf [file jo2c00232_si_001.pdf]

Supporting Information for

Polarized, V-shaped, Conjoined Bis-coumarins: From Lack of Dipole Moment Alignment to High Brightness

Łukasz Kielesiński, Irena Deperasińska, Olaf Morawski, Kateryna V. Vygranenko, Erik T. Ouellette and Daniel T. Gryko**

dtgryko@icho.edu.pl

deper@ifpan.edu.pl

Table of Contents

1. Synthetic part. S2
2. Crystallographic data. S3
3. Spectroscopic data. S5
4. Computational results. S11
5. NMR Spectra. S16
6. HRMS Spectra. S31

1. Synthetic part.

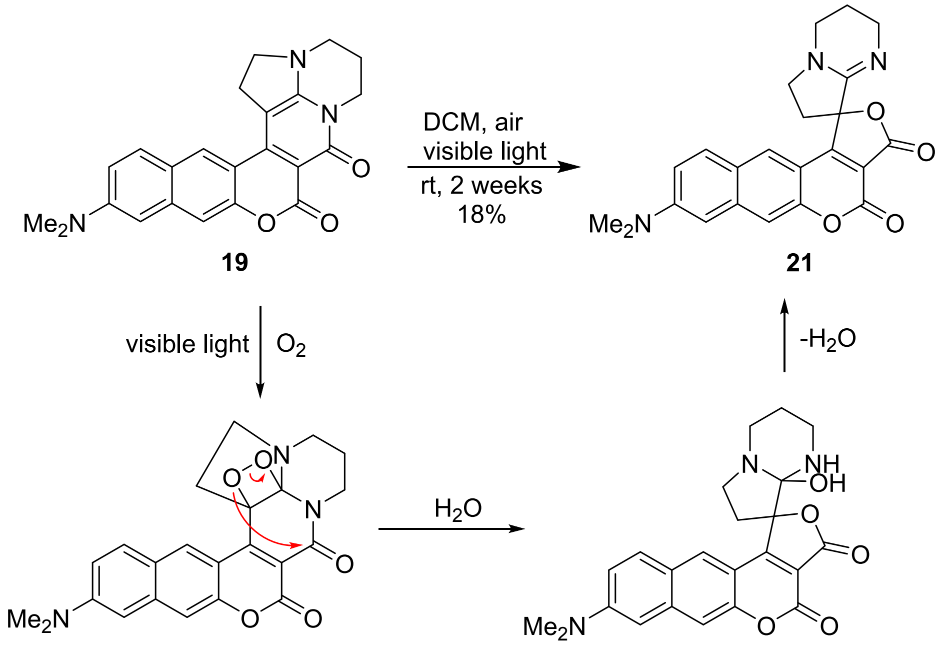

Scheme S1. Proposed reaction mechanism for the formation of spiro-coumarin **21**.

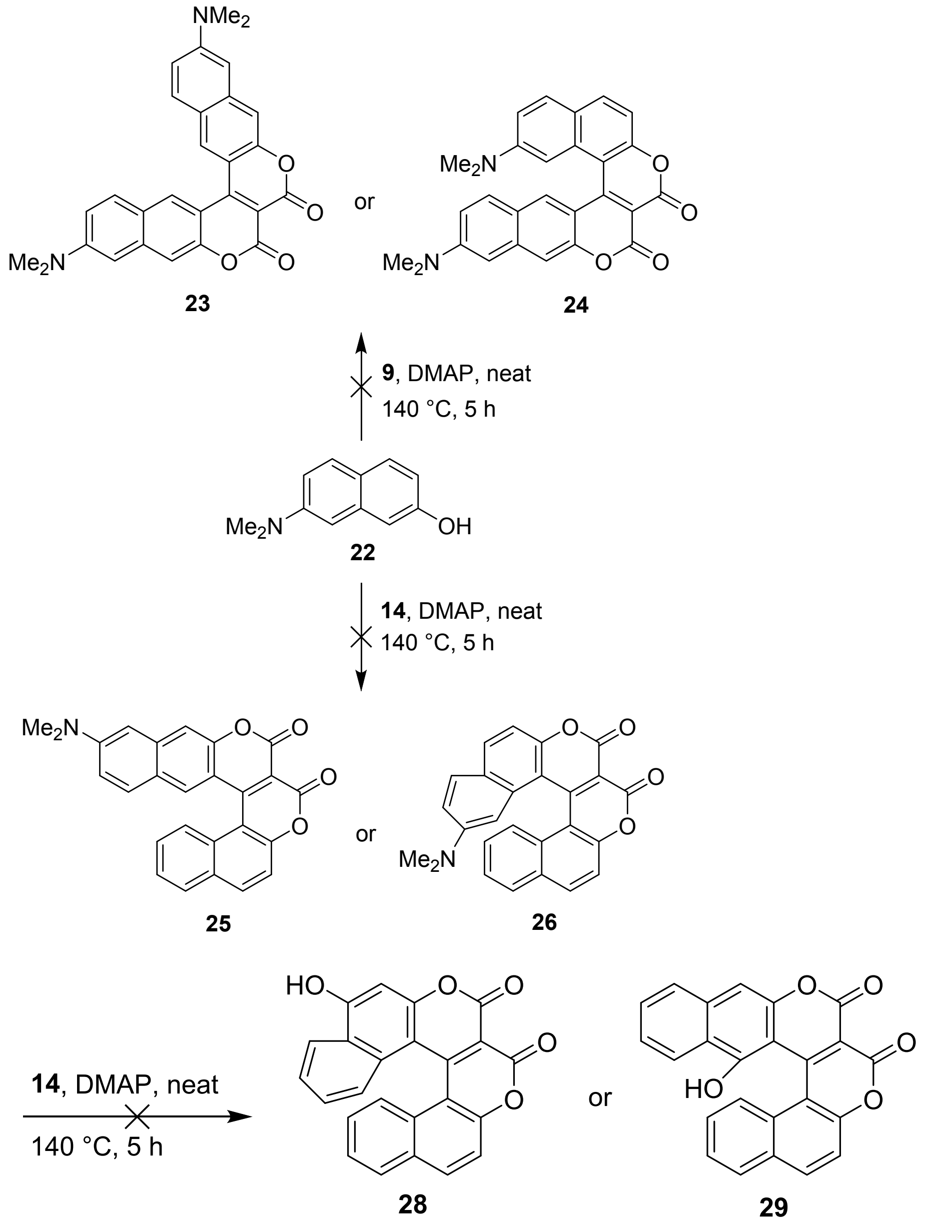

Scheme S2. Synthesis of helical bis-coumarins **23-26**, **28** and **29**.

2. Crystallographic data.

Table S1. Crystallographic data for spiro-coumarin **21**.

**Chemical formula**	$C_{24}H_{22}Cl_3N_3O_4$
**Formula weight**	522.79 g/mol
**Temperature**	100 K
**Wavelength**	0.72880 Å
**Crystal size**	0.04 x 0.04 x 0.04 mm^3
**Crystal habit**	orange shard

**Crystal system**	monoclinic	
**Space group**	$P2_1/n$	
**Unit cell dimensions**	a = 8.6134(5) Å	α = 90°
	b = 17.4046(10) Å	β = 92.411(2)°
	c = 15.1421(9) Å	γ = 90°
**Volume**	2268.0(2) $Å^3$	
**Z**	4	
**Diffractometer**	Bruker D8 with PHOTON-II CPAD detector	
**Radiation source**	synchrotron	
**Reflections collected**	32008	
**Independent reflections**	4640 [R(int) = 0.1001, R(sigma) = 0.0683]	
**Tmin, Tmax**	0.626, 0.690	
**Absorption correction**	multi-scan	
**Refinement method**	Full-matrix least-squares on F^2	
**Restraints / parameters**	0 / 309	
**Goodness-of-fit on F^2**	1.041	
**Final R indices**	[I=> 2σ(I)]	R1 = 0.0502, wR2 = 0.1262
	all data	R1 = 0.0663, wR2 = 0.1380

Single orange crystal of $C_{24}H_{22}Cl_3N_3O_4$ was selected on a Bruker D8 with PHOTON-II CPAD detector diffractometer. The crystal was kept at 100 K during data collection. Using Olex2 [1], the structure was solved with the SHELXT [2] structure solution program using Intrinsic Phasing and refined with the SHELXL [3] refinement package using Least Squares minimisation.

[1] Dolomanov, O.V., Bourhis, L.J., Gildea, R.J, Howard, J.A.K.; Puschmann, H. *J. Appl. Cryst*. **2009**, *42*, 339-341.
[2] Sheldrick, G.M. Acta Cryst. **2015**, *A71*, 3-8.
[3] Sheldrick, G.M. Acta Cryst. **2015**, *C71*, 3-8.

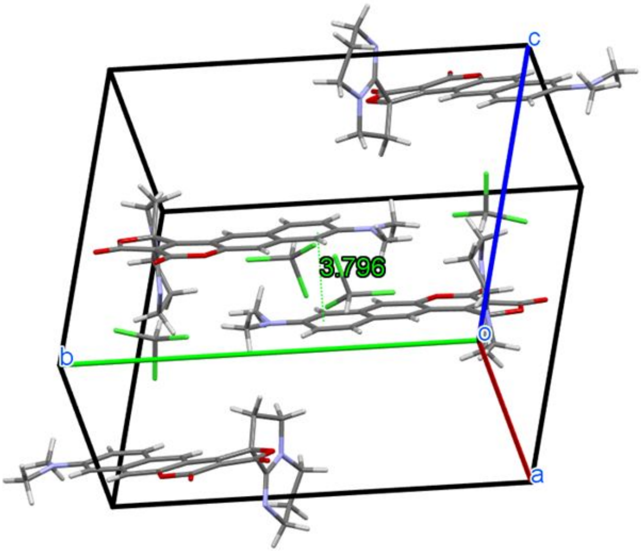

Figure S1. Packing diagram of crystals in elementary cell of spiro-coumarin **21**.

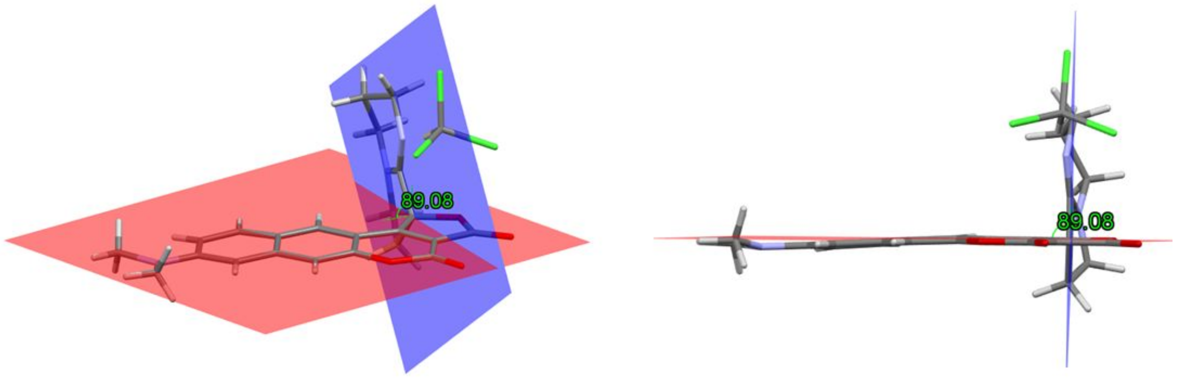

Figure S2. The plane and the value of dihedral angle between coumarin moiety and DBN ring.

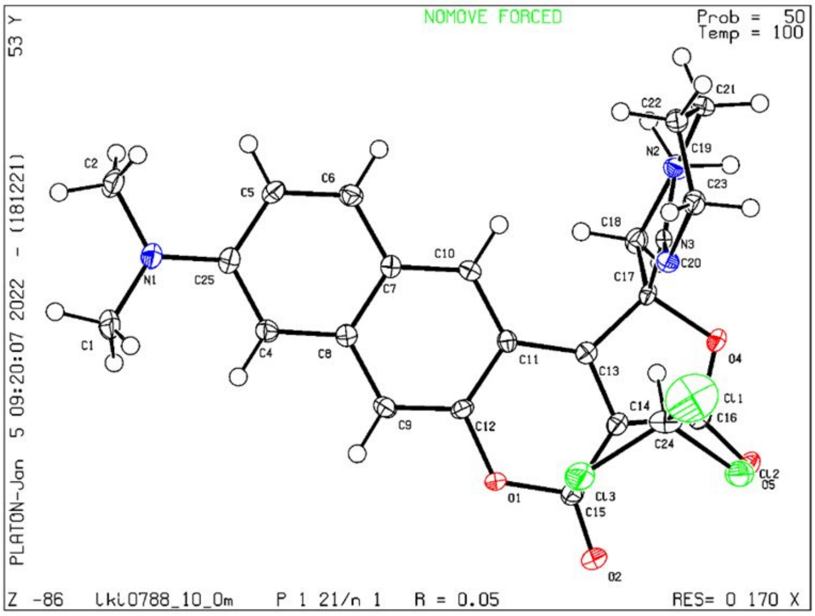

Figure S3. Thermal ellipsoid plot for spiro-coumarin **21** with 50% probability level of ellipsoids contour.

3. Spectroscopic data.

Lippert-Mattaga equations

In the Lippert-Mataga formulas, solvatochromism of absorption and fluorescence spectra can be related to modified Lippert-Mataga solvent polarity parameter f'(ε,n)=(ε-1)/(2ε+1) – ½×(n^2-1)/(2n^2+1):[1]

$$hc\nu^{abs} = hc\nu_0^{abs} - \frac{2\overrightarrow{\mu_g}\left(\overrightarrow{\mu_e} - \overrightarrow{\mu_g}\right)}{a^3}\left[\frac{\varepsilon - 1}{2\varepsilon + 1} - \frac{1}{2}\frac{n^2 - 1}{2n^2 + 1}\right] \qquad (1a)$$

$$hc\nu^{flu} = hc\nu_0^{flu} - \frac{2\overrightarrow{\mu_e}\left(\overrightarrow{\mu_e} - \overrightarrow{\mu_g}\right)}{a^3}\left[\frac{\varepsilon - 1}{2\varepsilon + 1} - \frac{1}{2}\frac{n^2 - 1}{2n^2 + 1}\right] \qquad (1b)$$

where $hc\nu^{abs}$ and $hc\nu^{flu}$ are the spectral positions of a solvent-equilibrated absorption and fluorescence spectra maxima, $hc\nu_0^{abs}$ and $hc\nu_0^{flu}$ are the relevant maxima in gas phase, μ_g and μ_e are the ground and excited state dipole moments of the solute, a is the cavity radius, ε is dielectric constant of the solvent and n its refraction index. When the change of the dipole moment, Δμ is nearly perpendicular to the dipole moment μ then scalar product of these vectors is close to zero and the solvatochromic shift expressed with formulas (1) shall be small despite large dipole moment and large increase of it upon optical excitation.

[1] Kapturkiewicz, A.; Herbich, J.; Karpiuk, J.; Nowacki, J. *J. Phys. Chem. A* **1997**, *101*, 2332-2344.

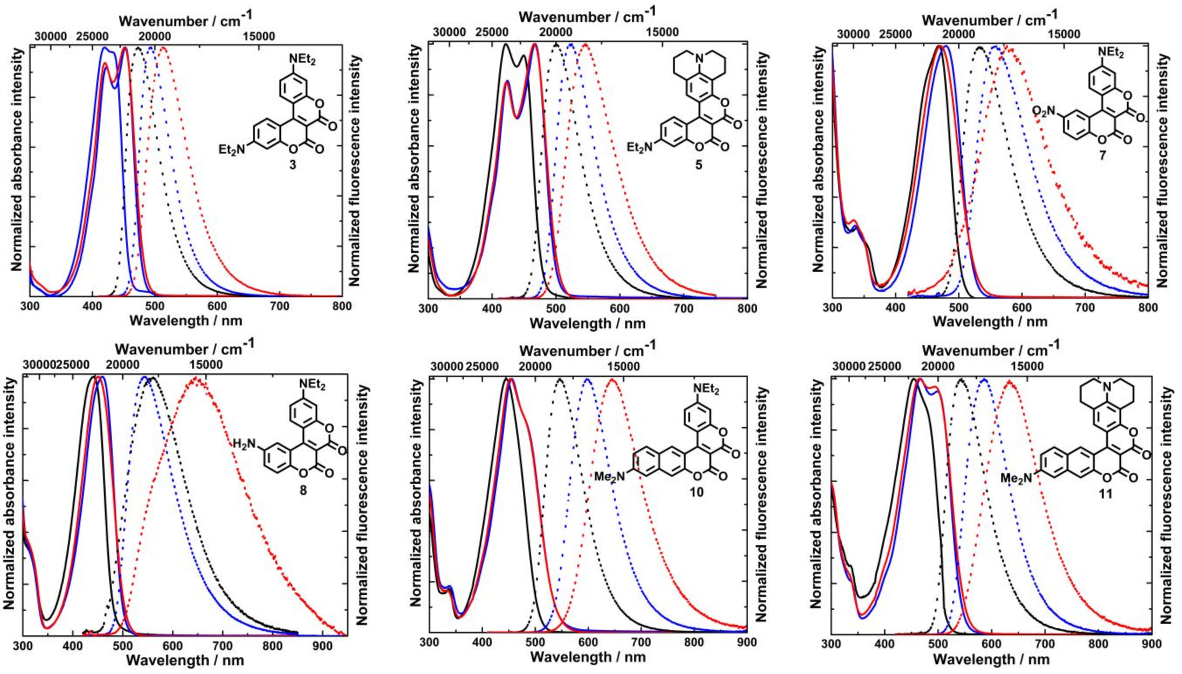

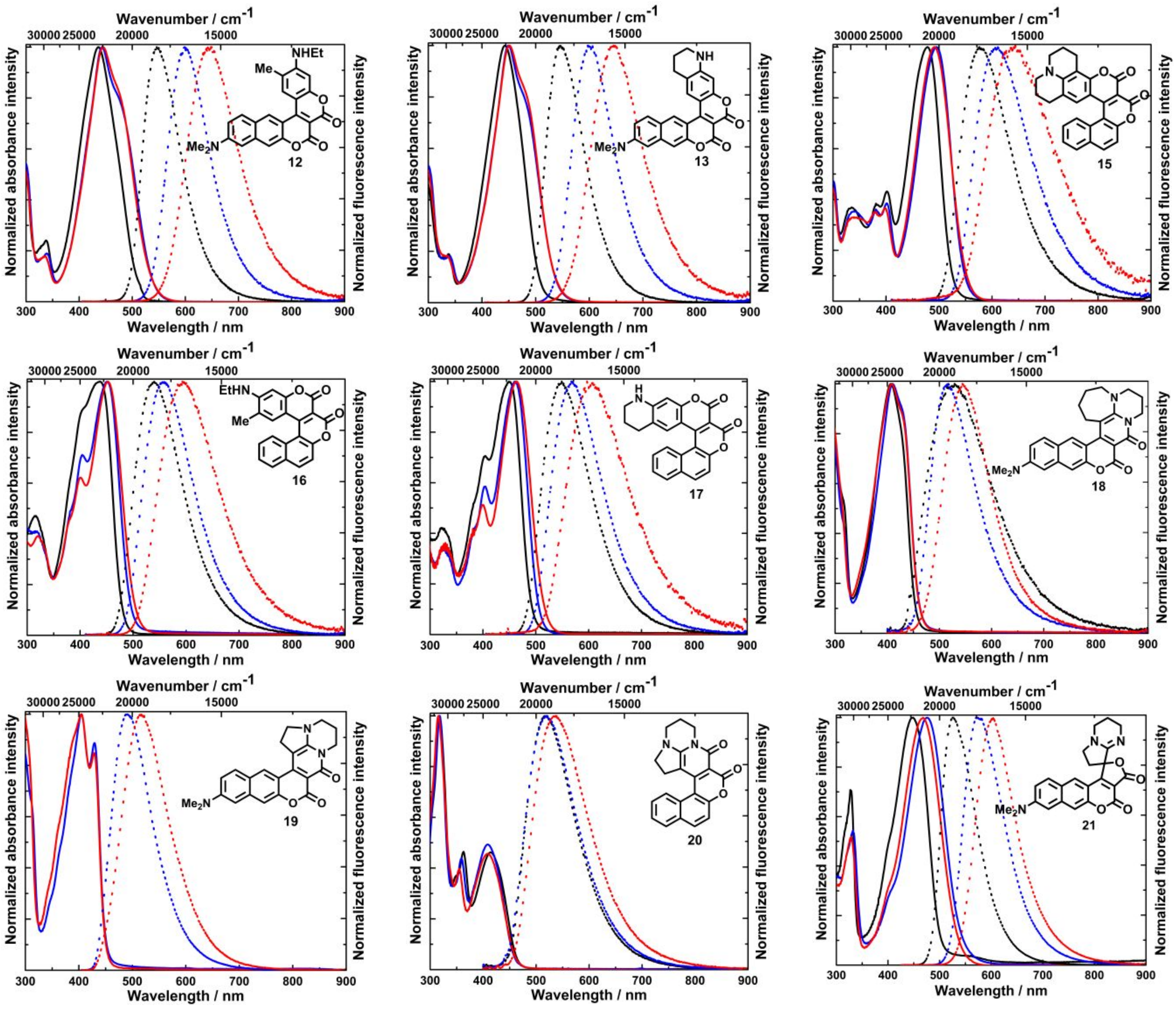

Figure S4. Absorption (solid line) and emission (dot line) spectra of dyes **3** (excited at 382 nm), **5** (excited at 382 nm), **7** (excited at 410 nm), **8** (excited at 410 nm), **10** (excited at 400 nm), **11** (excited at 410 nm), **12** (excited at 395 nm), **13** (excited at 395 nm), **15** (excited at 400 nm), **16** (excited at 400 nm), **17** (excited at 395 nm), **18** (excited at 390 nm), **19** (excited at 395 nm), **20** (excited at 390 nm) and **21** (excited at 420 nm) measured in toluene (black), DCM (blue) and ACN (red).

Table S2. Fluorescence decay times (τ_1, τ_2) and the amplitude of decay components (A_1, A_2) of compounds **3**, **5**, **7**, **8**, **10-13** and **15-21** in toluene, DCM and ACN.

comp.	solvent	τ_1 [ns]	A_1	τ_2 [ns]	A_2	τ_{aver}^{a} [ns]
**3**	toluene	1.45				1.45
	DCM	1.87				1.87
	ACN	2.59				2.59
**5**	toluene	2.06				2.06
	DCM	3.96				3.96
	ACN	4.36				4.36
**7**	toluene	6.55				6.55
	DCM	0.82				0.82

	ACN	0.034	9.01	2.38	0.1	0.06
**8**	toluene	0.81	2.35	5.29	0.27	1.27
	DCM	1.52	4.91	6.32	2.97	3.33
	ACN	0.53	0.59	3.70	0.07	0.92
**10**	toluene	1.45				1.45
	DCM	5.17				5.17
	ACN	5.16				5.16
**11**	toluene	0.78	2.19	4.96	0.27	1.24
	DCM	3.78				3.78
	ACN	4.74				4.74
**12**	toluene	3.69				3.69
	DCM	5.58				5.58
	ACN	4.82				4.82
**13**	toluene	3.40				3.40
	DCM	5.51				5.51
	ACN	4.60				4.60
**15**	toluene	5.59				5.59
	DCM	4.14				4.14
	ACN	1.22				1.22
**16**	toluene	2.23				2.23
	DCM	3.41				3.41
	ACN	2.18				2.18
**17**	toluene	3.06				3.06
	DCM	3.88				3.88
	ACN	1.45				1.45
**18**	toluene	0.28	15.55	2.75	0.45	0.35
	DCM	0.43	13.80	2.08	0.59	0.50
	ACN	0.30	3.78	0.79	2.30	0.49
**19**	toluene					
	DCM	2.32				2.32
	ACN	3.21				3.21
**20**	toluene	1.86				1.86
	DCM	1.58	9.39	2.97	6.89	2.17
	ACN	2.44				2.44
**21**	toluene	5.10				5.10
	DCM	5.50				5.50
	ACN	5.60				5.60

[a] $\tau_{aver} = (A_1*\tau_1*\tau_1 + A_2*\tau_2*\tau_2)/(A_1*\tau_1 + A_2*\tau_2)$.

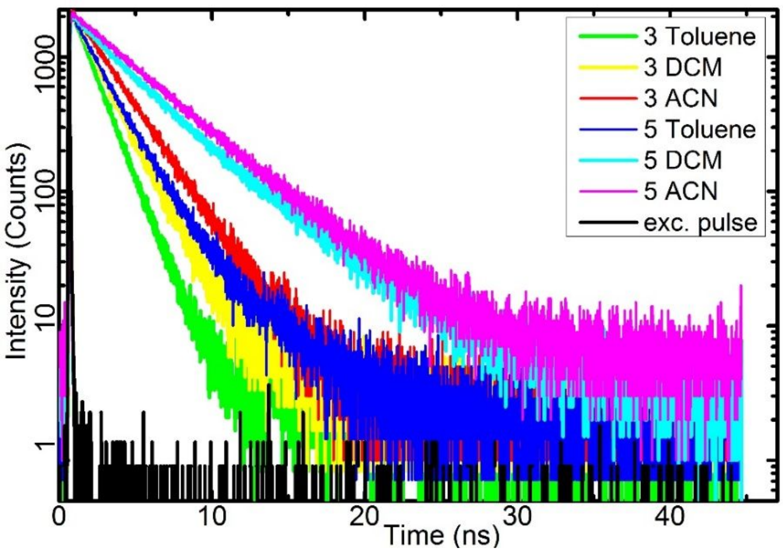
3 Toluene
3 DCM
3 ACN
5 Toluene
5 DCM
5 ACN
exc. pulse
Intensity (Counts)
Time (ns)

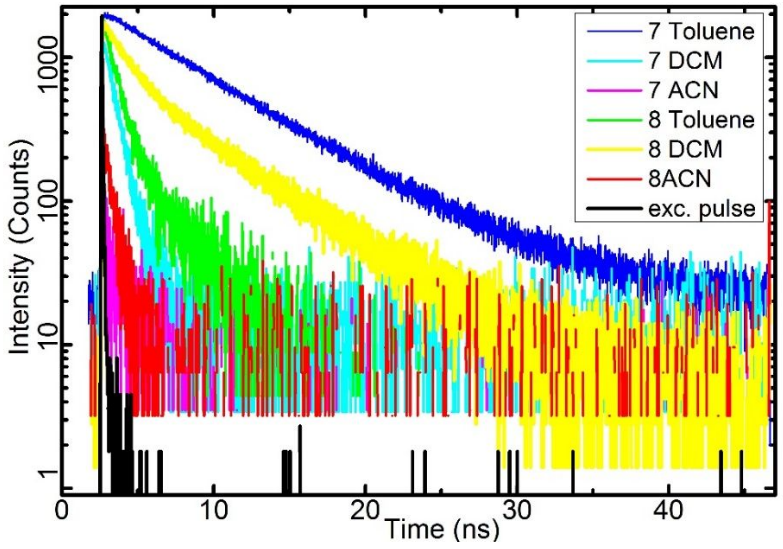
7 Toluene
7 DCM
7 ACN
8 Toluene
8 DCM
8ACN
exc. pulse
Intensity (Counts)
Time (ns)

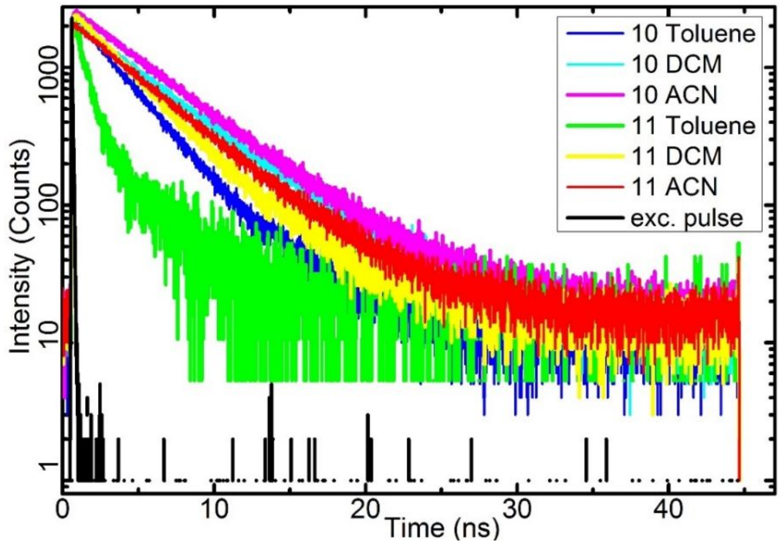
10 Toluene
10 DCM
10 ACN
11 Toluene
11 DCM
11 ACN
exc. pulse
Intensity (Counts)
Time (ns)

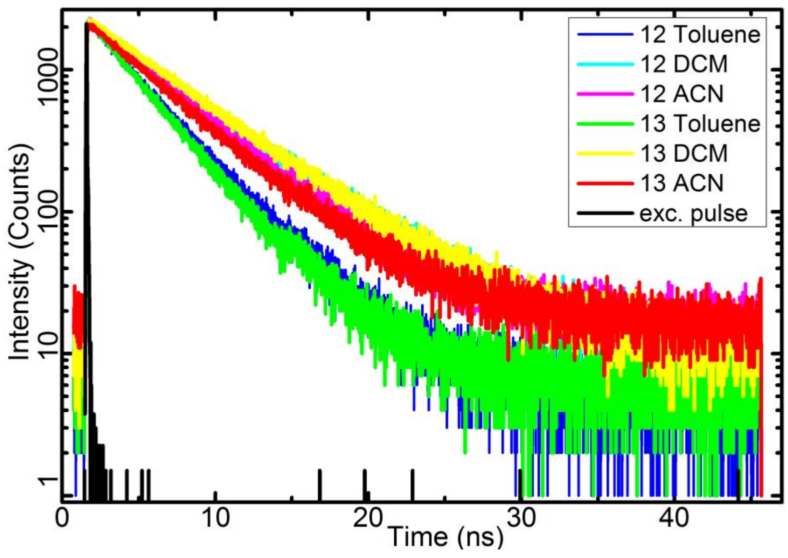

Intensity (Counts)
Time (ns)
12 Toluene
12 DCM
12 ACN
13 Toluene
13 DCM
13 ACN
exc. pulse

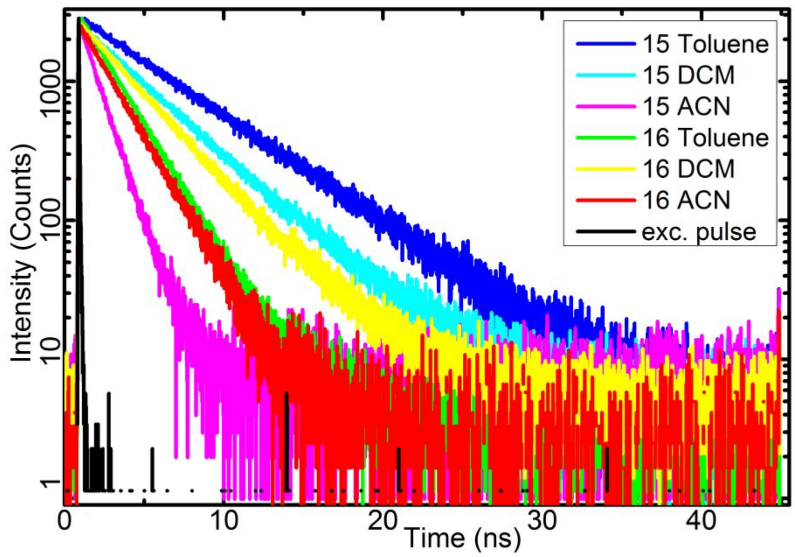

Intensity (Counts)
Time (ns)
15 Toluene
15 DCM
15 ACN
16 Toluene
16 DCM
16 ACN
exc. pulse

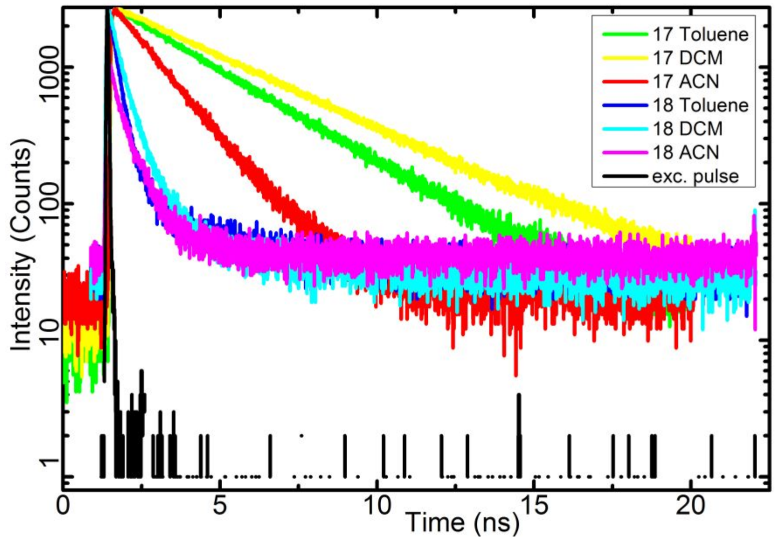

Intensity (Counts)
Time (ns)
17 Toluene
17 DCM
17 ACN
18 Toluene
18 DCM
18 ACN
exc. pulse

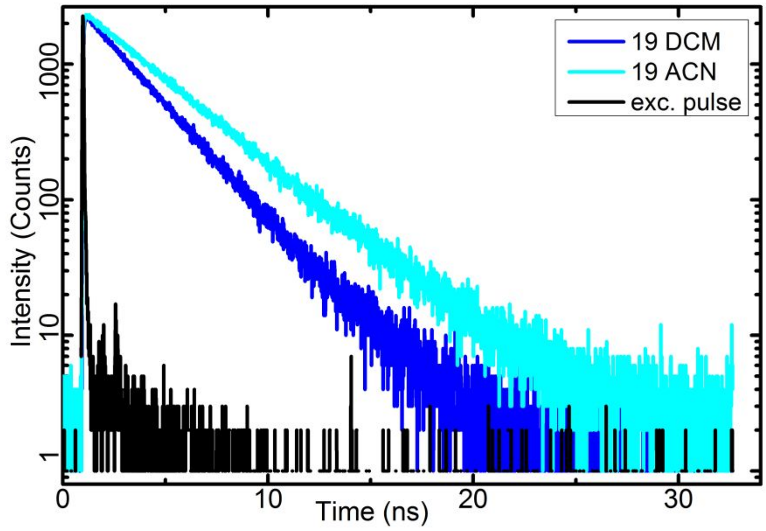

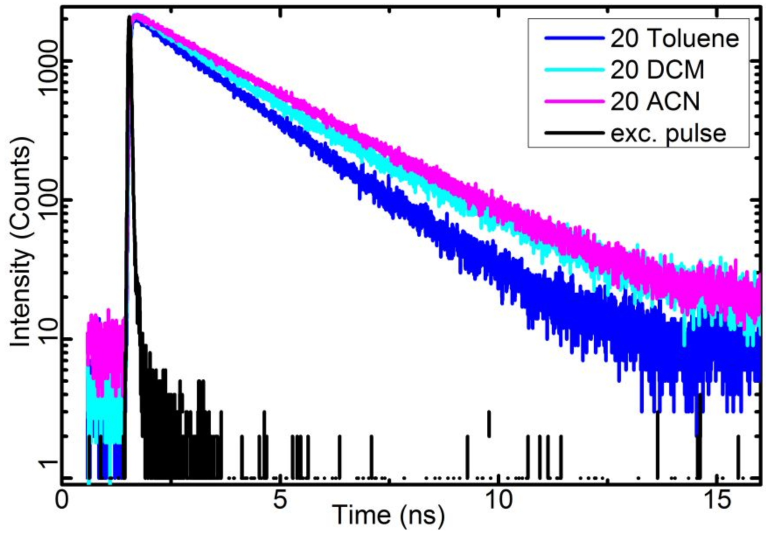

Figure S5. Semilogarithmic plots of fluorescence decays of compounds **3** and **5** (observed at 500 nm recorded with resolution 12.2 ps per channel), **7** and **8** (observed at 550 nm recorded with resolution 12.2 ps per channel), **10** and **11** (observed at 550 nm (toluene), 600 nm (DCM) and 620 nm (ACN) recorded with resolution 12.2 ps per channel), **12** and **13** (observed at 530 nm (toluene), 600 nm (DCM) and 630 nm (ACN) recorded with resolution 12.2 ps per channel), **15** and **16** (recorded with resolution 12.22 ps per channel), **17** and **18** (recorded with resolution 6.11 ps per channel), **19** (recorded with resolution 12.2 ps per channel) and **20** (recorded with resolution 4.07 ps per channel).

4. Computational results.

Table S3. The energies of electronic transitions $S_0 \rightarrow S_1$ and $S_1 \rightarrow S_0$ corresponding to structures previously optimized in the ground and excited state in three solvents: toluene, DCM and ACN. Calculations made with the use of two functionals: B3LYP and M06/6-31G(d,p). The last two columns contain the radiative transition rate constants for fluorescence, resulting from the calculated values of energy and the oscillator strength (f) for the $S_1 \rightarrow S_0$ transition.

comp.	solvent	B3LYP/6-31G(d,p)				M06/6-31G(d,p)				B3LYP	M06
		$S_0 \rightarrow S_1$		$S_1 \rightarrow S_0$		$S_0 \rightarrow S_1$		$S_1 \rightarrow S_0$		$k_r^{calc.}$/ 10^9	$k_r^{calc.}$/ 10^9
		[nm]	f	[nm]	f	[nm]	f	[nm]	f	[sec$^-$]	[sec$^-$]
**3**	toluene	412.5	0.8229	451.9	0.5716	391.1	0.8574	421.7	0.6707	0.187	0.251
	DCM	435.2	0.9855	459.6	0.9460	411.7	1.0195	433.5	1.0475	0.299	0.372
	ACN	443.9	1.0428	466.3	1.0650	419.5	1.0754	441.9	1.1165	0.327	0.381
**5**	toluene	435.8	0.7251	478.6	0.4891					0.142	
	DCM	449.9	0.9016	492.2	0.7195					0.198	
	ACN	459.7	0.9678	498.8	0.8110					0.217	
**7**	toluene	458.3	0.0650	631.3	0.0028	420.5	0.4609	545.7	0.0052	0.000	0.001
	DCM	476.7	0.1190	663.1	0.0057	438.2	0.5918	562.1	0.0109	0.001	0.002
	ACN	482.2	0.1560	662.8	0.0069	444.4	0.6457	566.9	0.0139	0.001	0.003
**8**	toluene	442.1	0.2249	597.7	0.1038	420.5	0.2406	547.5	0.1300	0.019	0.029
	DCM	460.1	0.3617	603.6	0.1504					0.028	
	ACN	467.5	0.4174	608.9	0.1694	443.9	0.4392	564.5	0.2061	0.031	0.043
**10**	toluene	476.1	0.5061	542.7	0.3056					0.069	
	DCM	504.1	0.6900	557.3	0.5559					0.119	
	ACN	514.7	0.7570	565.3	0.6504					0.136	
**11**	toluene	475.7	0.5629	539.0	0.3311					0.008	
	DCM	504.0	0.7549	552.5	0.6241					0.136	
	ACN	514.8	0.8253	560.5	0.7388					0.157	
**12**	toluene	472.5	0.4919	543.4	0.2879					0.065	
	DCM	499.2	0.6810	556.4	0.5301					0.114	
	ACN	509.6	0.7509	563.8	0.6246					0.131	
**13**	toluene	473.6	0.4783	545.1	0.2809					0.0630	
	DCM	500.3	0.6645	556.7	0.5233					0.113	
	ACN	510.5	0.7335	563.6	0.6194					0.130	
**15**	toluene	454.6	0.4179	583.8	0.2165					0.0423	
	DCM	477.4	0.5620	588.4	0.3393					0.0653	
	ACN	486.4	0.6174	592.8	0.3913	467.5	0.6658	544.4	0.4606	0.0742	0.104
**16**	toluene	424.4	0.4085	547.2	0.1994					0.0444	
	DCM	444.2	0.5436	547.4	0.3216					0.0716	
	ACN	452.0	0.5960	549.3	0.3751					0.0829	
**17**	toluene	430.9	0.3985	554.9	0.1979					0.0429	
	DCM	451.7	0.5363	557.0	0.3187					0.0685	
	ACN	459.9	0.5899	559.6	0.3713					0.0790	
**18**	toluene	418.0	0.5089	479.8	0.3297	399.8	0.5580	449.9	0.4317	0.0955	0.142
	DCM	433.5	0.6854	488.3	0.5872					0.164	
	ACN	436.2	0.7231	493.5	0.6802	418.0	0.8009	468.3	0.7959	0.186	0.242
**19**	toluene	405.3	0.5723	459.3	0.3772	390.1	0.6181	435.9	0.4621	0.0955	0.162
	DCM	417.1	0.7723	466.0	0.6736					0.164	
	ACN	422.7	0.8102	470.1	0.7783	404.9	0.8733	450.0	0.8780	0.186	0.289
**20**	toluene	399.7	0.1303	511.8	0.0705					0.0179	
	DCM	404.8	0.1970	509.5	0.1200					0.0308	
	ACN	406.9	0.2249	509.9	0.1440					0.0369	

Table S4. Comparison of the transitions energy, the oscillator strengths and dipole moments of the selected conjoint coumarins with the transitions energies, oscillator strengths and dipole moments of theirs' components.

comp.	conjoint coumarin			components of the coumarin		
	E_{abs} [nm]	f	μ [D]	E_{abs} [nm]	f	μ [D]
**3**	412.5	0.8229	14.85	348.31	0.5688	8.14
				348.31	0.5688	8.14

**5**	425.8	0.7251	14.97	359.68	0.5178	8.13
				348.31	0.5688	8.14
**10**	476.1	0.5061	16.16	348.31	0.5689	8.14
				416.48	0.4324	9.09
**11**	475.7	0.5629	16.10	359.68	0.5178	8.13
				416.48	0.4324	9.09
**15**	454.6	0.4179	14.03	344.93	0.2755	6.10
				359.68	0.5178	8.13
**19**	405.3	0.5723	13.51	416.48	0.4324	9.09
				315.64	0.2672	6.90

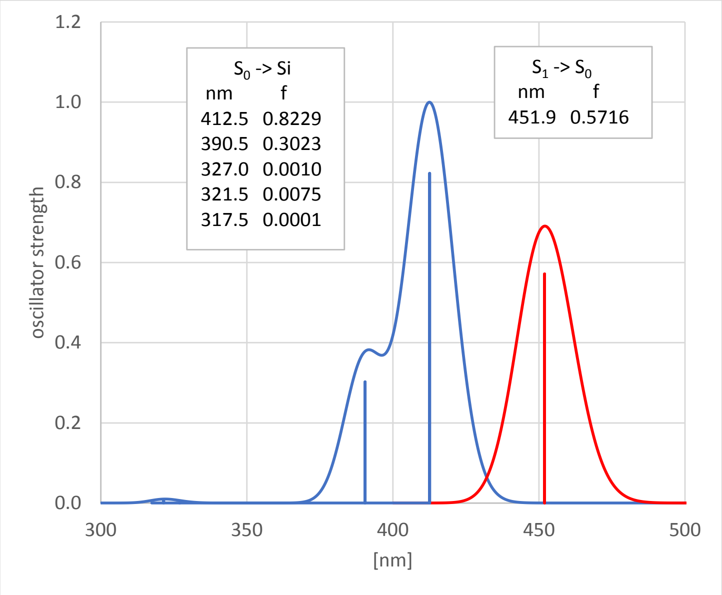

Figure S6. Calculated absorption and fluorescence spectra (**3**) in toluene as an example of the typical for conjoint coumarins overlapping of two energetically close transitions in absorption $S_0 \rightarrow S_1$ and $S_0 \rightarrow S_2$. The non-separation of these two bands introduces uncertainty in the estimates of the corresponding energies, as well as the magnitude of the Stokes shifts. Two energetically close transitions $S_0 \rightarrow S_1$ and $S_0 \rightarrow S_2$ correspond to excitations from two energetically close HOMO and HOMO-1 orbitals (each located on one of the conjoint coumarin arms) to the LUMO orbital.

Correlation calculations/experiment

The graphs shown in Figure S7 and Figure S8 below are a comparison of the calculated (B3LYP method, Table S3) and measured (Table 1, article) values of the absorption and fluorescence energy and the coumarin fluorescence rate constants. Despite numerous deviations, there is a correlation between the experimental data and the calculation results (±1200 cm^{-1}, ie ±0.15 eV,

which is a rather typical value for such comparisons).[1] It should be remembered that the sources of the discrepancies lie not only in the quality of the calculation method, but also in the fact that the quantities calculated for the electron transitions are compared with the maxima of the vibrationally not separated bands. Moreover, in the case of absorption, the band corresponding to the excitation to state S_1 is not separated with excitation band to state S_2 (Figure S6). Two energetically close transitions $S_0 \rightarrow S_1$ and $S_0 \rightarrow S_2$. they correspond to excitations from two energetically close HOMO and HOMO-1 orbitals (each located on one of the conjoint coumarin arms) to the LUMO orbital, located throughout the molecule (Figure S9 and Figure S10).

[1] a) Budzák, Š.; Charaf-Eddin, A.; Medved, M.; Gryko, D. T.; Jacquemin, D. *Comput. Theor. Chem.* **2016**, *1076*, 57-64. b) Xue, Y.; An, L.; Zheng, Y.; Zhang, L.; Gong, X.; Qian, Y.; Liu, Y. *Comput. Theor. Chem.* **2012**, *981*, 90-98. c) Zhao, Y.; Truhlar, D. G. *Theor. Chem. Acc.* **2008**, *120*, 215-241.

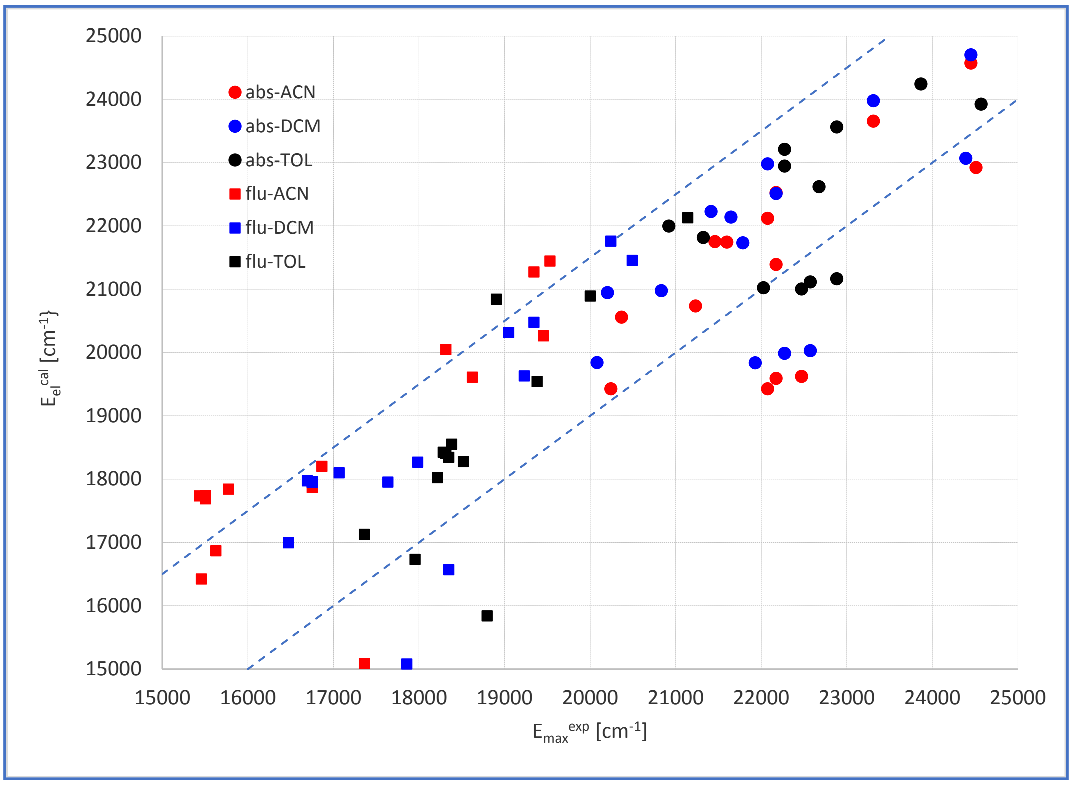

Figure S7. Calculated absorption and fluorescence energies of V-shaped coumarins in three solvents of different polarity, (toluene, DCM and ACN) as a function of the experimentally

determined energies. Points the most deviating from the correlation correspond to the compounds **7** and **8**

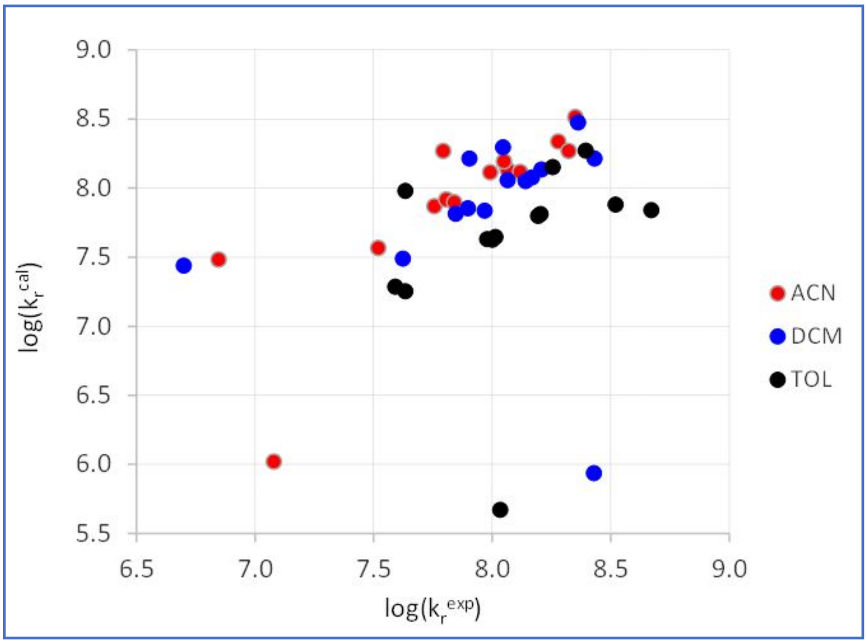

Figure S8. Calculated values of the rate constants for radiative transitions rate for V-shaped coumarins in the toluene, DCM and ACN as a function of their experimentally determined values. Points clearly deviating from the correlation correspond to the compounds **7** and **18**.

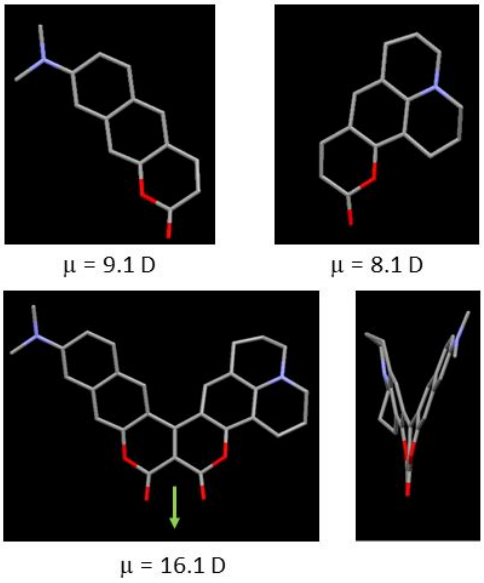

Figure S9. Coumarin **11** as the combination of two coumarins. Molecule **11** is V-shaped not only on the plane but also in space. The dipole moment of dye **11** has a large value, slightly less than the sum of the dipole moments of the components, and its direction is close to the central C-C bond shared by both coumarin moieties.

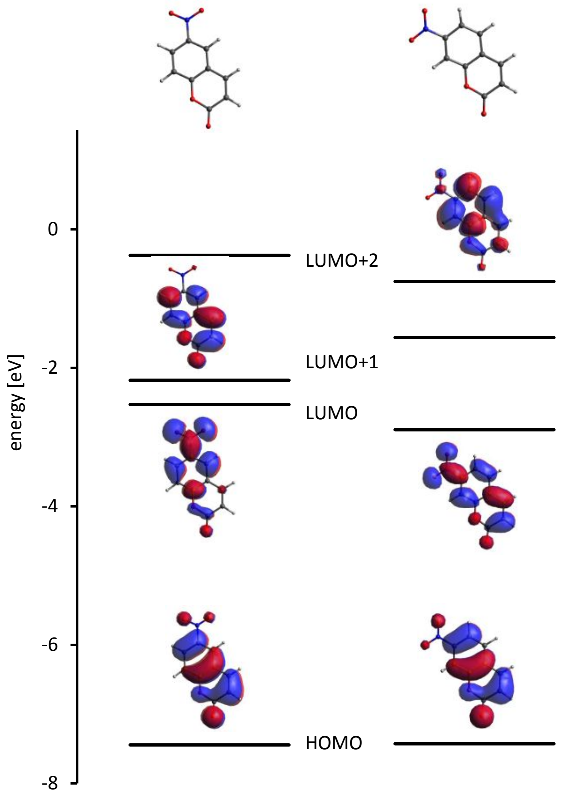

Figure S10. Comparison of energetic arrangement of molecular orbitals of 6- and 7-nitrocoumarins. The LUMO orbitals of both molecules participate in the formation of the S_1 state of the dark forms of compound **7** and its counterpart V-coumarin. Orbital LUMO+1 of 6-nitrocoumarin, energetically close to LUMO, participates in the formation of the S_1 state of the bright form of compound **7**. An orbital with a similar shape in the case of 7-nitrocoumarin is LUMO+2, too high lying to play a similar role.

5. NMR Spectra.

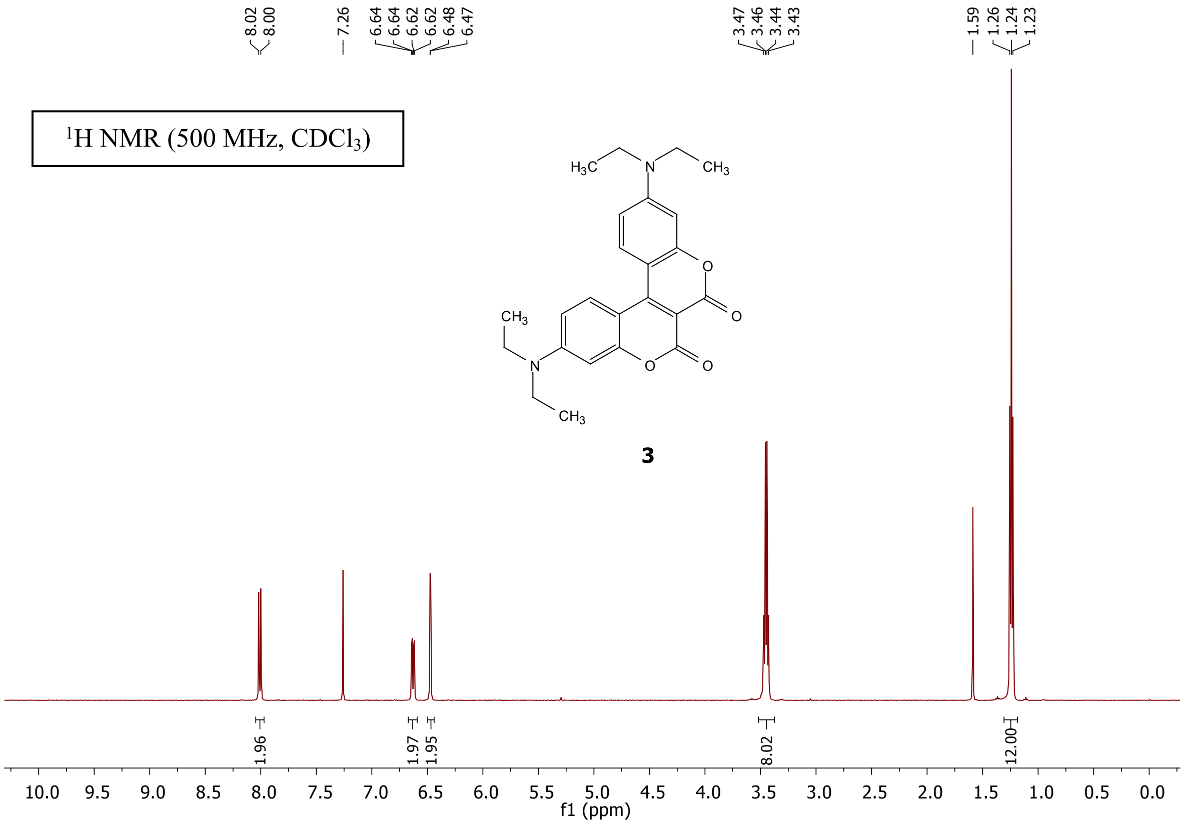

^{1}H NMR (500 MHz, $CDCl_3$)
8.02
8.00
7.26
6.64
6.64
6.62
6.62
6.48
6.47
3.47
3.46
3.44
3.43
1.59
1.26
1.24
1.23
H_3C
N
CH_3
O
O
CH_3
N
O
O
CH_3
3
1.96
1.97
1.95
8.02
12.00
f1 (ppm)

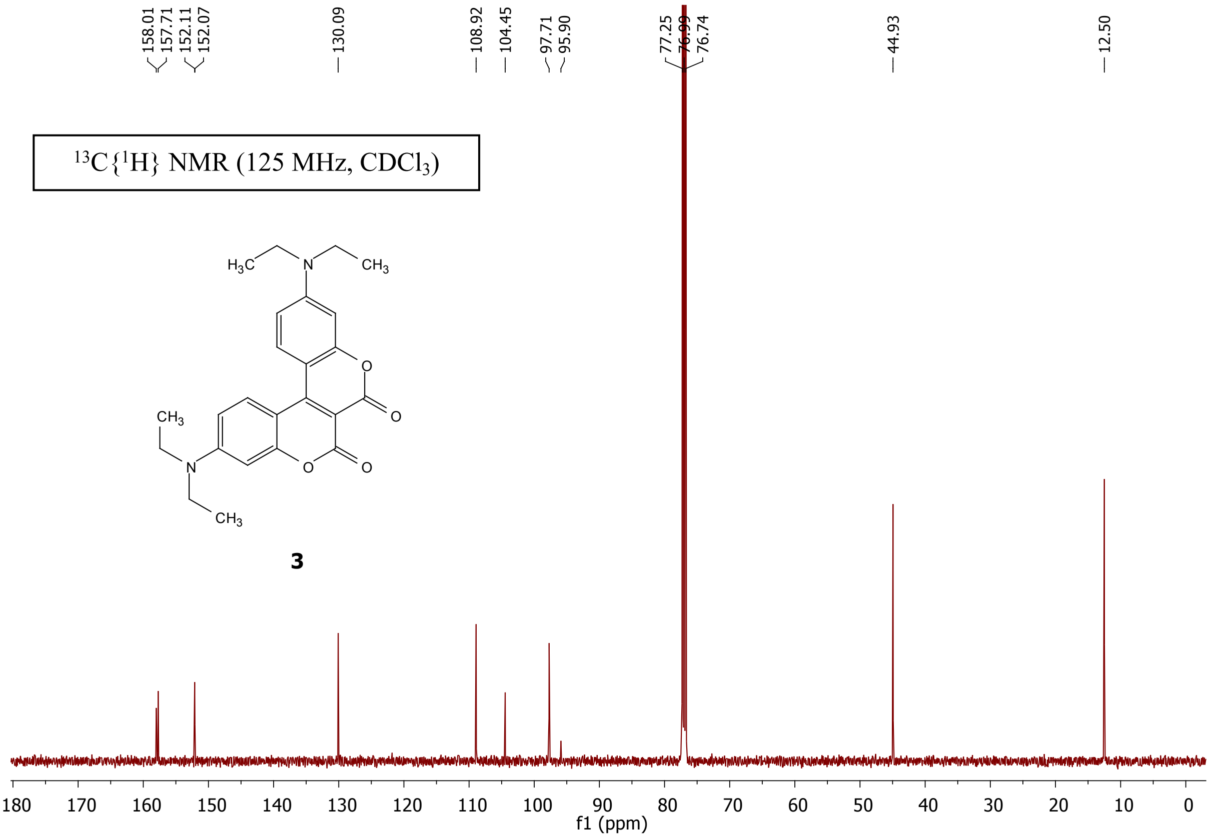

$^{13}C\{^{1}H\}$ NMR (125 MHz, $CDCl_3$)
158.01
157.71
152.11
152.07
130.09
108.92
104.45
97.71
95.90
77.25
76.99
76.74
44.93
12.50
H_3C
N
CH_3
O
O
CH_3
N
O
O
CH_3
3
f1 (ppm)

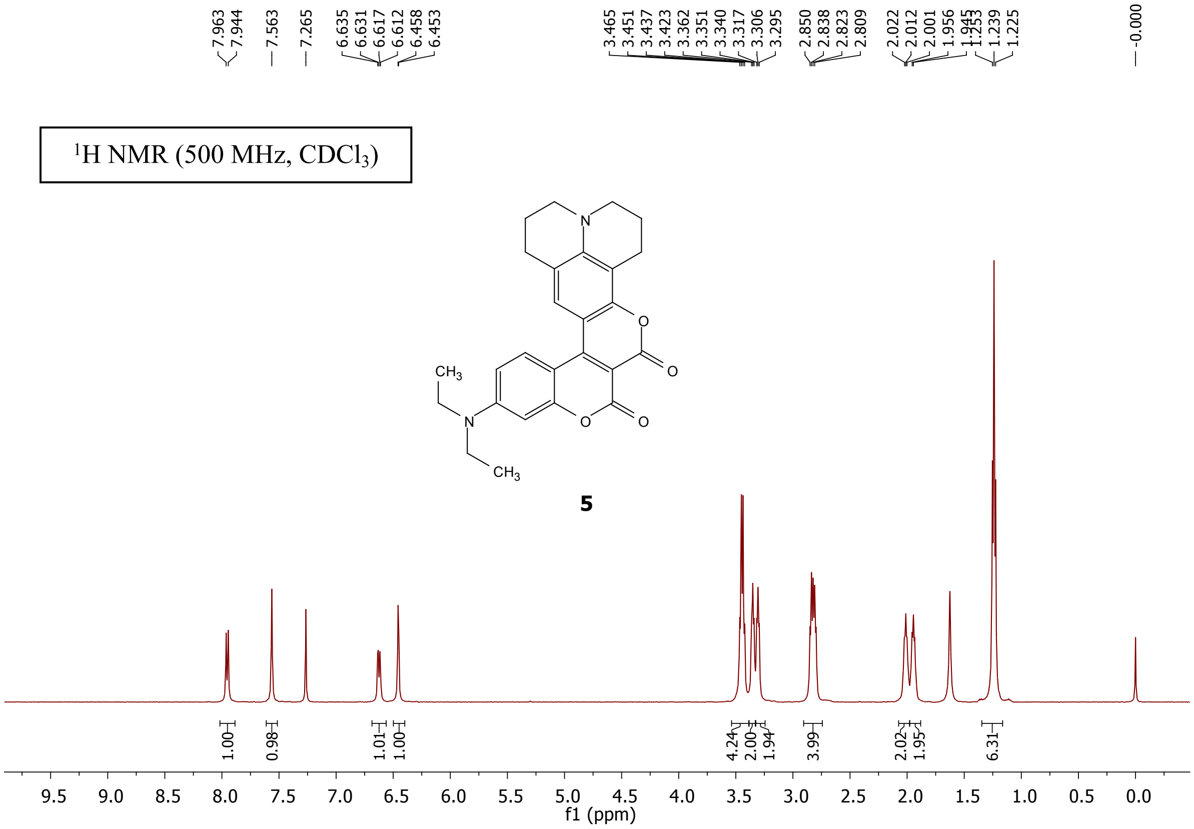

7.963
7.944
7.563
7.265
6.635
6.631
6.617
6.612
6.458
6.453
3.465
3.451
3.437
3.423
3.362
3.351
3.340
3.317
3.306
3.295
2.850
2.838
2.823
2.809
2.022
2.012
2.001
1.956
1.945
1.253
1.239
1.225
-0.000
^{1}H NMR (500 MHz, $CDCl_3$)
N
O
O
O
O
CH_3
N
CH_3
5
1.00
0.98
1.01
1.00
4.24
2.00
1.94
3.99
2.02
1.95
6.31
f1 (ppm)

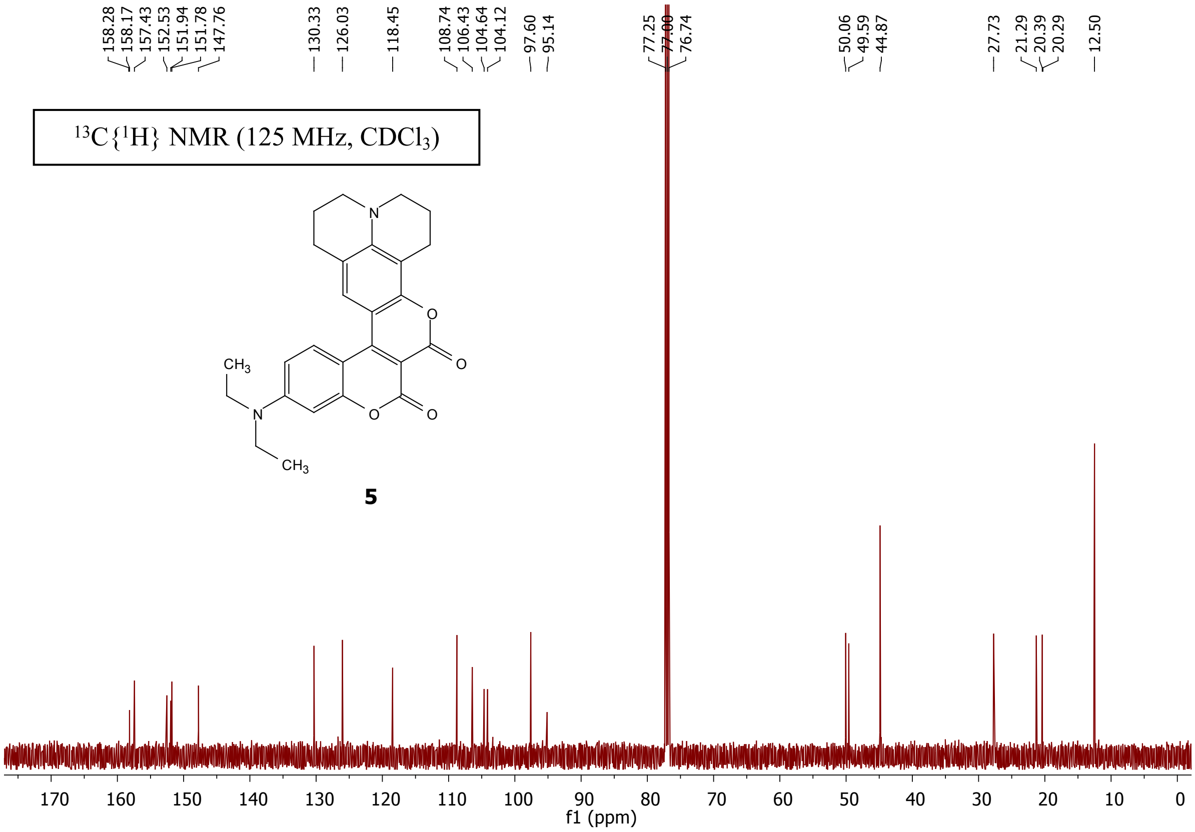

158.28
158.17
157.43
152.53
151.94
151.78
147.76
130.33
126.03
118.45
108.74
106.43
104.64
104.12
97.60
95.14
77.25
77.00
76.74
50.06
49.59
44.87
27.73
21.29
20.39
20.29
12.50
^{13}C{^{1}H} NMR (125 MHz, $CDCl_3$)
N
O
O
O
O
CH_3
N
CH_3
5
f1 (ppm)

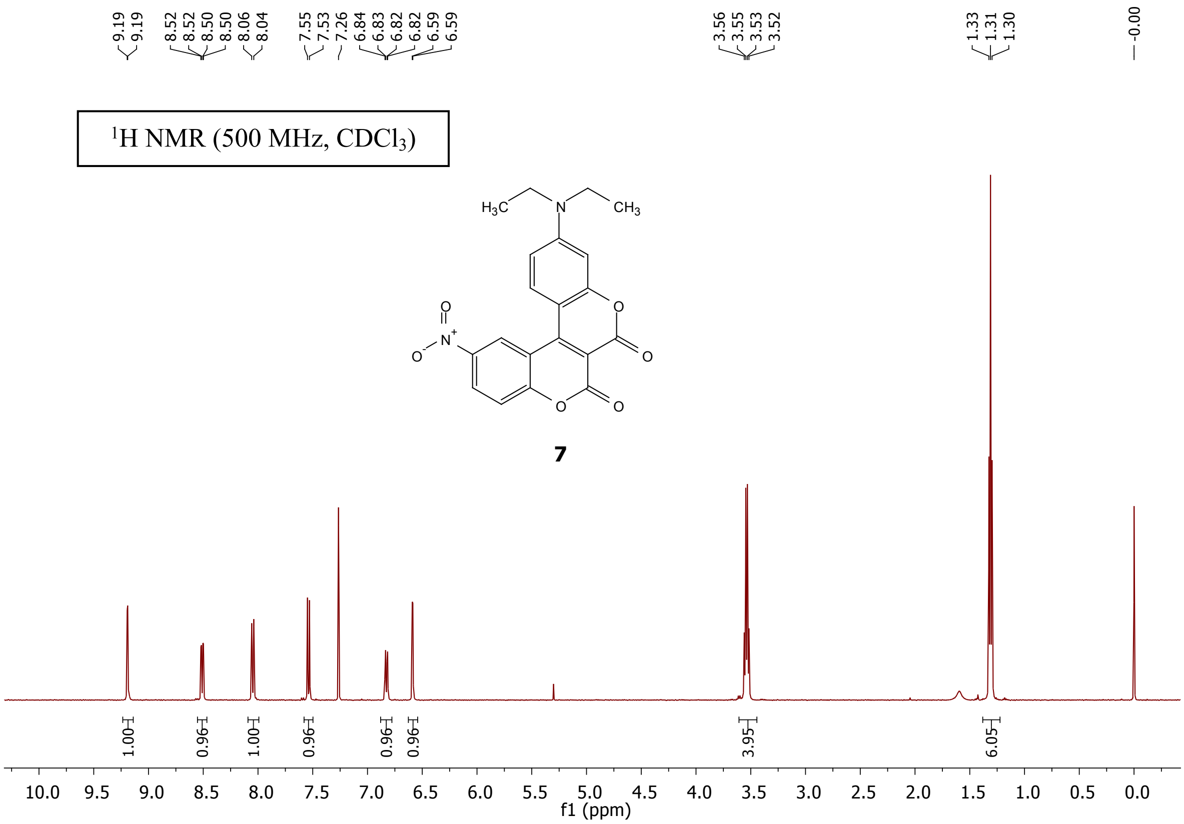
9.19
9.19
8.52
8.52
8.50
8.50
8.06
8.04
7.55
7.53
7.26
6.84
6.83
6.82
6.82
6.59
6.59
3.56
3.55
3.53
3.52
1.33
1.31
1.30
-0.00
¹H NMR (500 MHz, CDCl₃)
H₃C
N
CH₃
O
O
O
O
O
N⁺
O⁻
7
1.00
0.96
1.00
0.96
0.96
0.96
3.95
6.05
10.0 9.5 9.0 8.5 8.0 7.5 7.0 6.5 6.0 5.5 5.0 4.5 4.0 3.5 3.0 2.5 2.0 1.5 1.0 0.5 0.0
f1 (ppm)

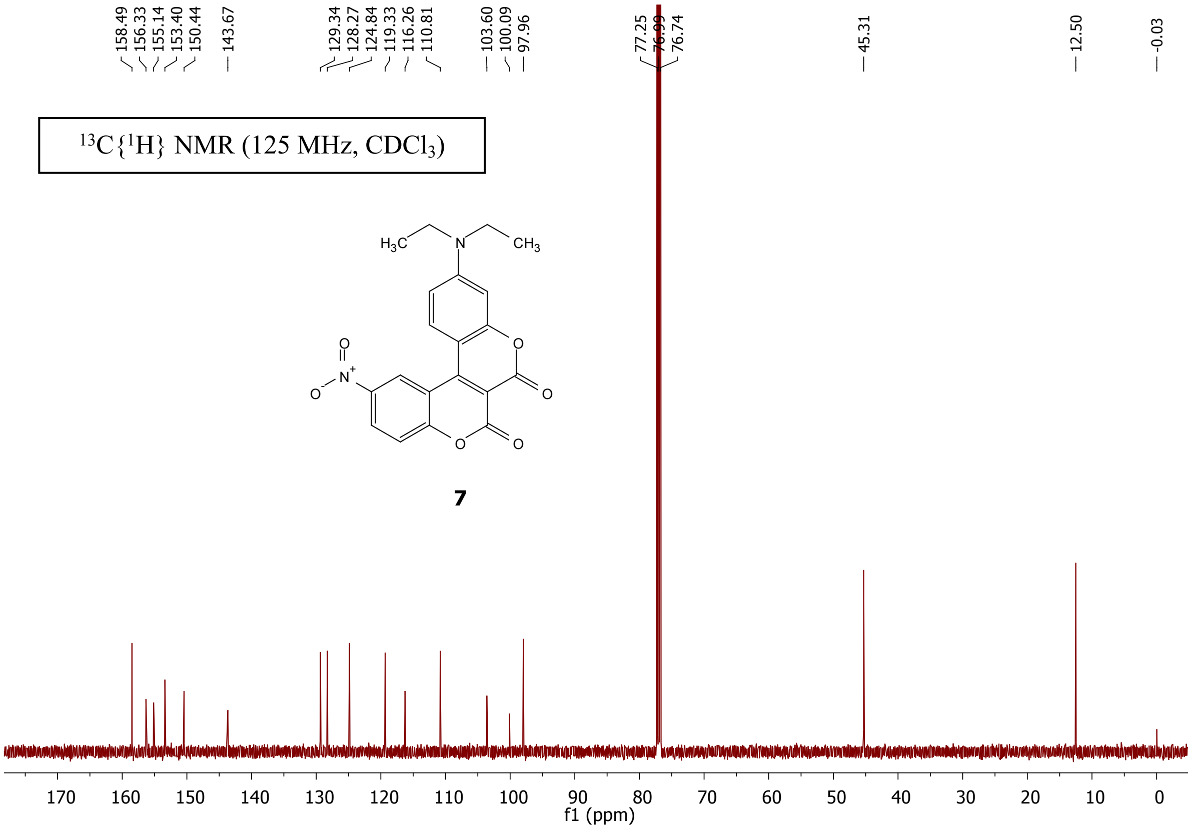
158.49
156.33
155.14
153.40
150.44
143.67
129.34
128.27
124.84
119.33
116.26
110.81
103.60
100.09
97.96
77.25
76.99
76.74
45.31
12.50
-0.03
¹³C{¹H} NMR (125 MHz, CDCl₃)
H₃C
N
CH₃
O
O
O
O
O
N⁺
O⁻
7
170 160 150 140 130 120 110 100 90 80 70 60 50 40 30 20 10 0
f1 (ppm)

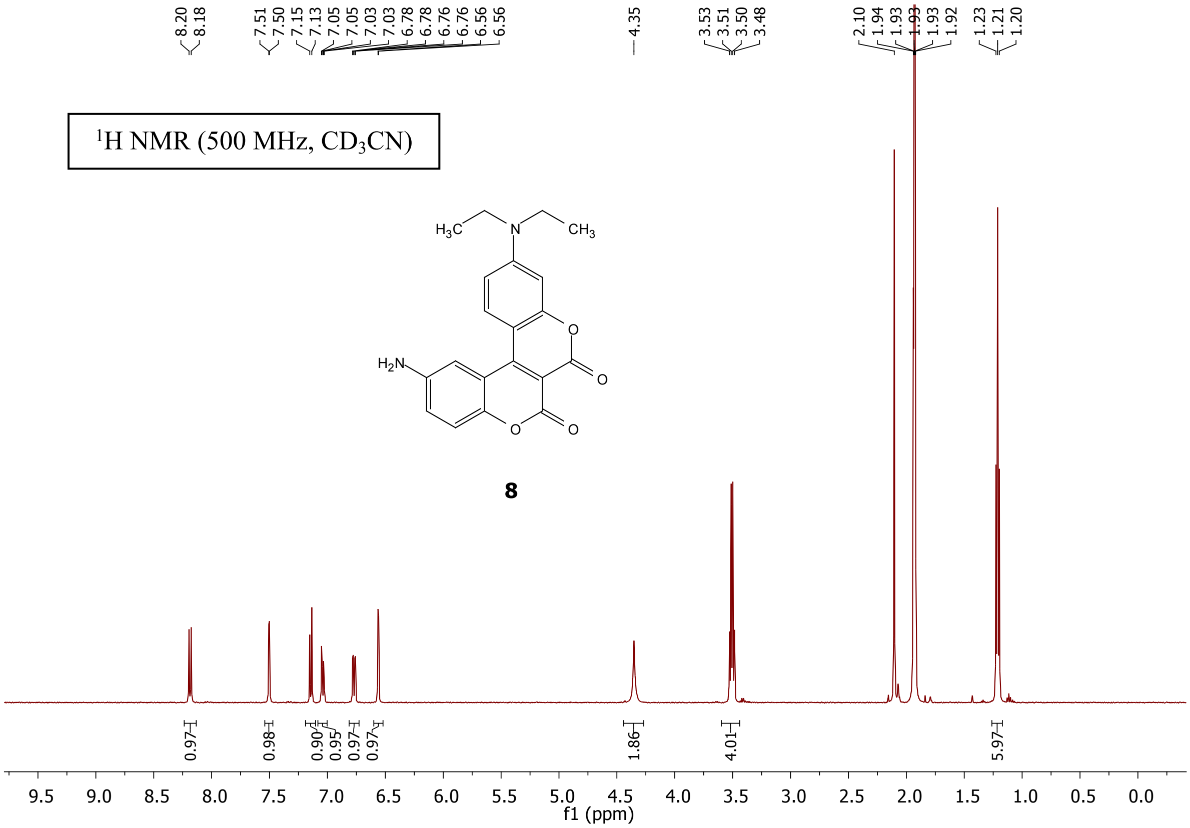

8.20
8.18
7.51
7.50
7.15
7.13
7.05
7.05
7.03
7.03
6.78
6.78
6.76
6.76
6.56
6.56
4.35
3.53
3.51
3.50
3.48
2.10
1.94
1.93
1.93
1.93
1.92
1.23
1.21
1.20
^{1}H NMR (500 MHz, CD_3CN)
H_3C
N
CH_3
H_2N
O
O
O
O
8
0.97
0.98
0.90
0.95
0.97
0.97
1.86
4.01
5.97
f1 (ppm)

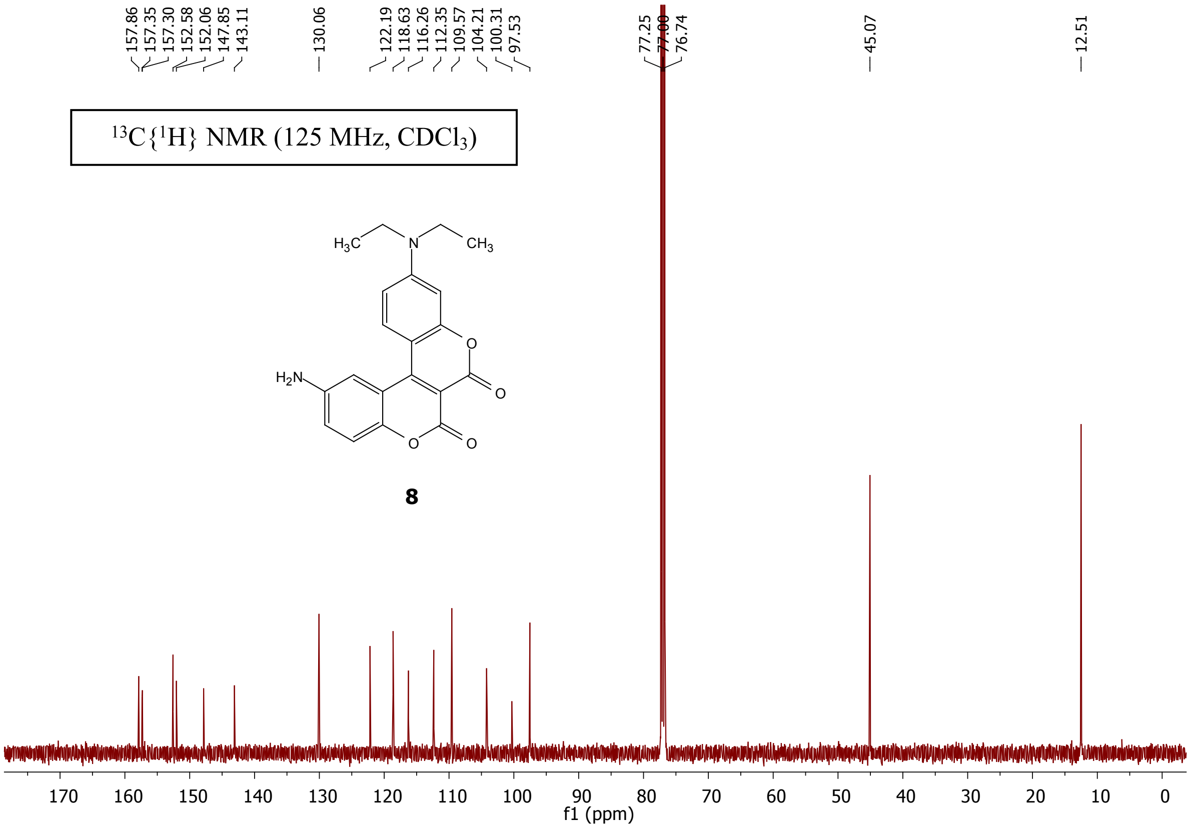

157.86
157.35
157.30
152.58
152.06
147.85
143.11
130.06
122.19
118.63
116.26
112.35
109.57
104.21
100.31
97.53
77.25
77.00
76.74
45.07
12.51
$^{13}C\{^1H\}$ NMR (125 MHz, $CDCl_3$)
H_3C
N
CH_3
H_2N
O
O
O
O
8
f1 (ppm)

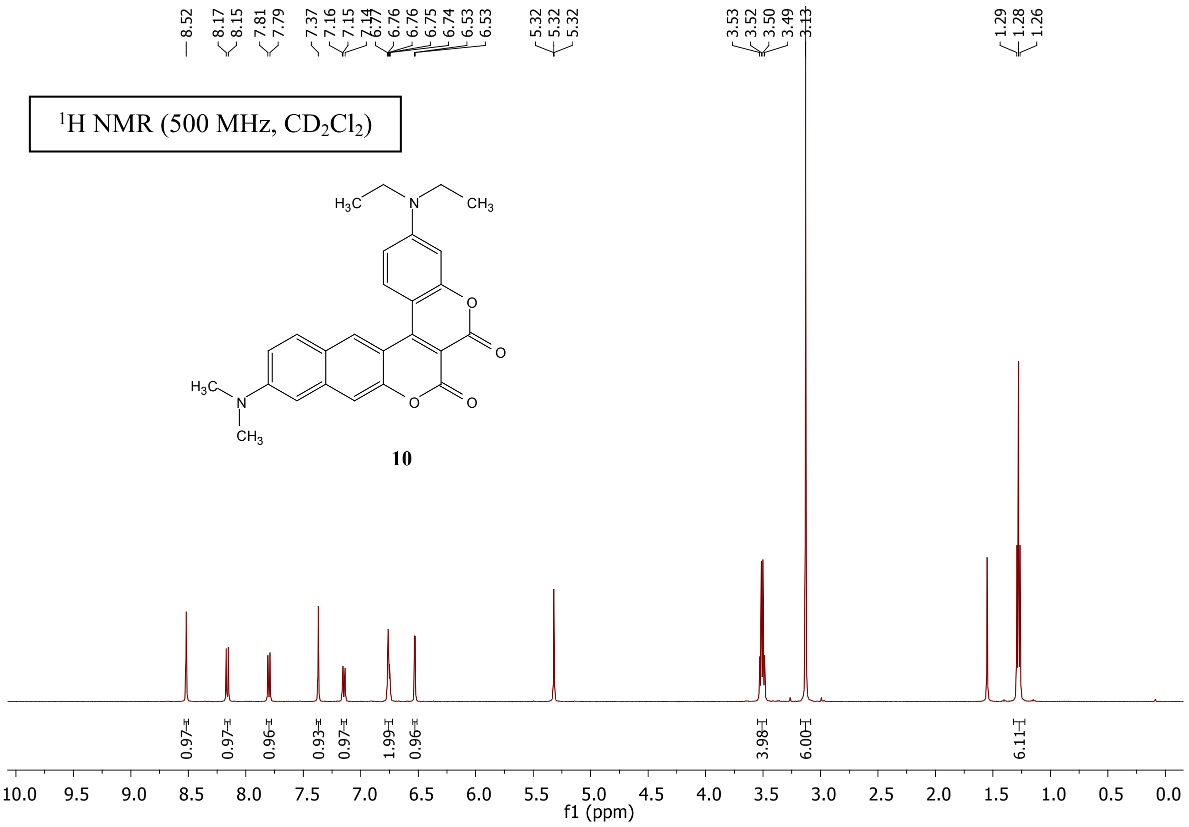

8.52
8.17
8.15
7.81
7.79
7.37
7.16
7.15
7.14
6.77
6.76
6.76
6.75
6.74
6.53
6.53
5.32
5.32
5.32
3.53
3.52
3.50
3.49
1.29
1.28
1.26
¹H NMR (500 MHz, CD₂Cl₂)
H₃C
N
CH₃
O
O
O
O
H₃C
N
CH₃
10
0.97
0.97
0.96
0.93
0.97
1.99
0.96
3.98
6.00
6.11
10.0
9.5
9.0
8.5
8.0
7.5
7.0
6.5
6.0
5.5
5.0
4.5
4.0
3.5
3.0
2.5
2.0
1.5
1.0
0.5
0.0
f1 (ppm)

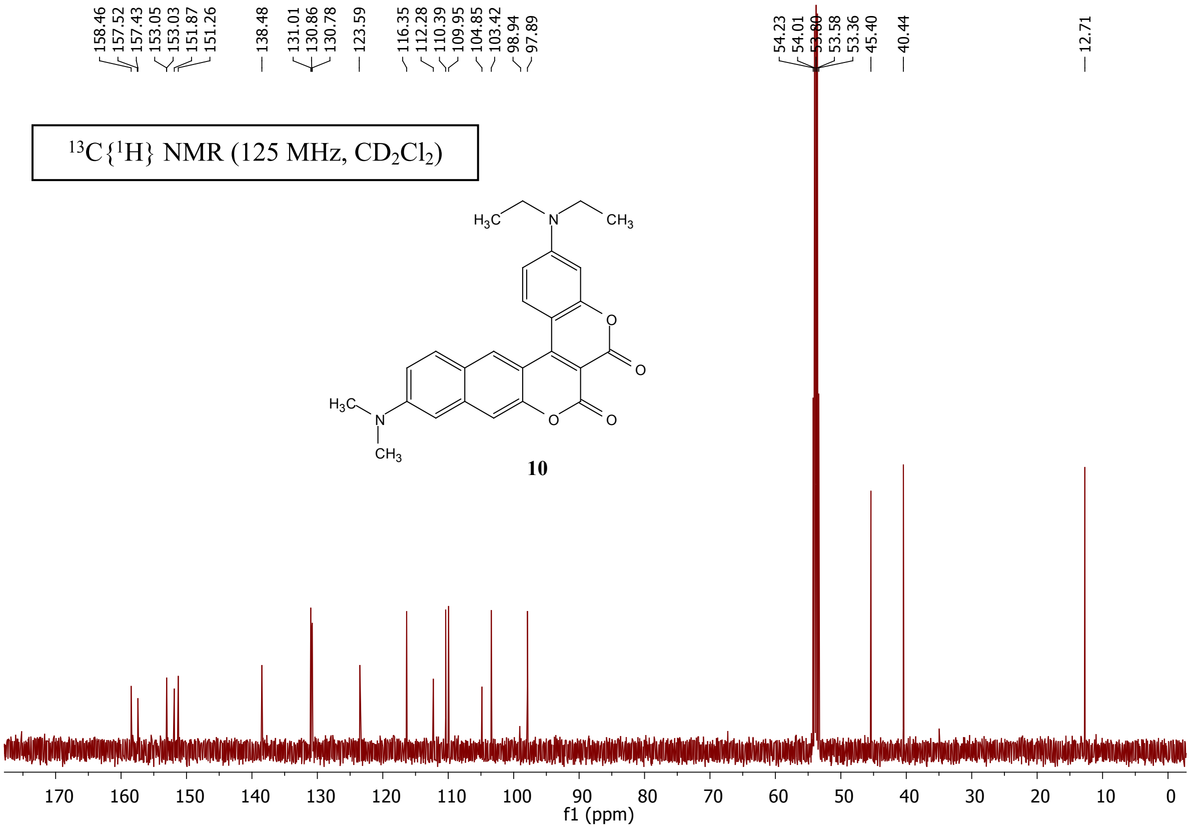

158.46
157.52
157.43
153.05
153.03
151.87
151.26
138.48
131.01
130.86
130.78
123.59
116.35
112.28
110.39
109.95
104.85
103.42
98.94
97.89
54.23
54.01
53.58
53.36
45.40
40.44
12.71
¹³C{¹H} NMR (125 MHz, CD₂Cl₂)
H₃C
N
CH₃
O
O
O
O
H₃C
N
CH₃
10
170
160
150
140
130
120
110
100
90
80
70
60
50
40
30
20
10
0
f1 (ppm)

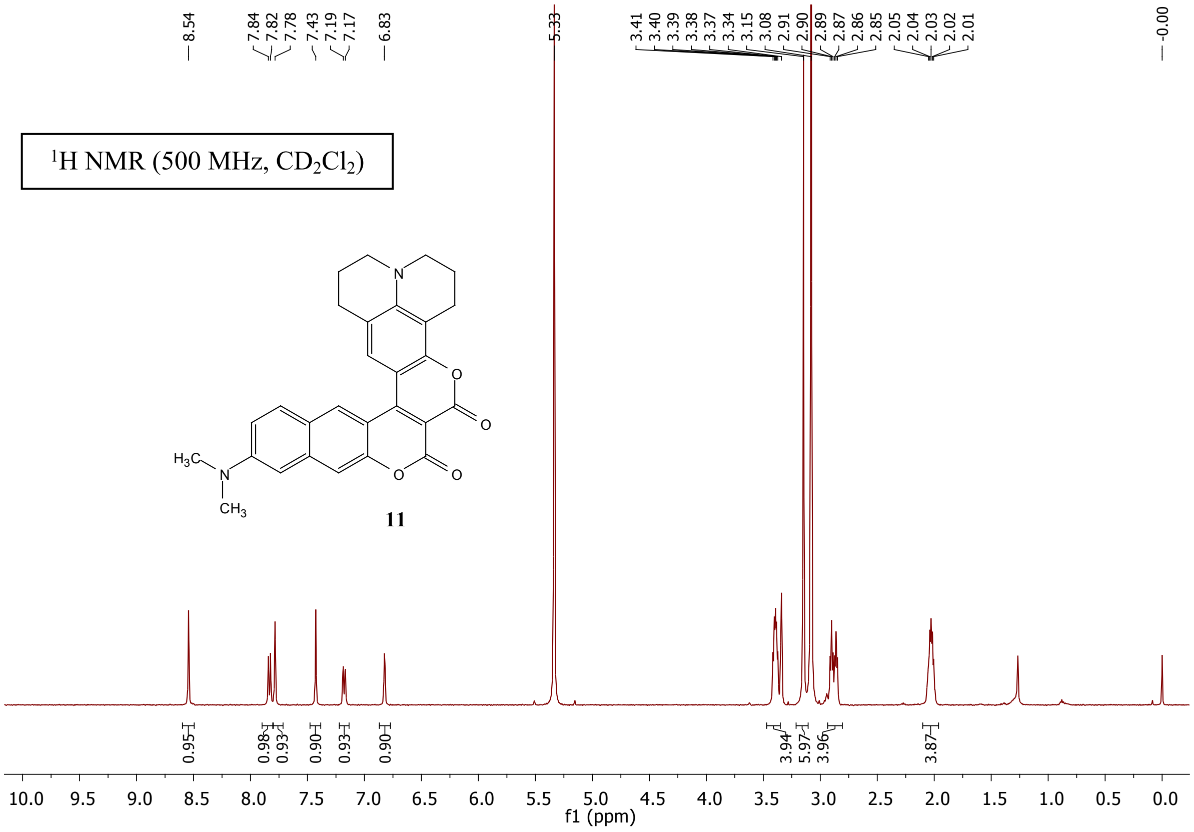
8.54
7.84
7.82
7.78
7.43
7.19
7.17
6.83
5.33
3.41
3.40
3.39
3.38
3.37
3.34
3.15
3.08
2.91
2.90
2.89
2.87
2.86
2.85
2.05
2.04
2.03
2.02
2.01
-0.00
1H NMR (500 MHz, CD2Cl2)
N
O
O
O
O
H3C
N
CH3
11
0.95
0.98
0.93
0.90
0.93
0.90
3.94
5.97
3.96
3.87
10.0 9.5 9.0 8.5 8.0 7.5 7.0 6.5 6.0 5.5 5.0 4.5 4.0 3.5 3.0 2.5 2.0 1.5 1.0 0.5 0.0
f1 (ppm)

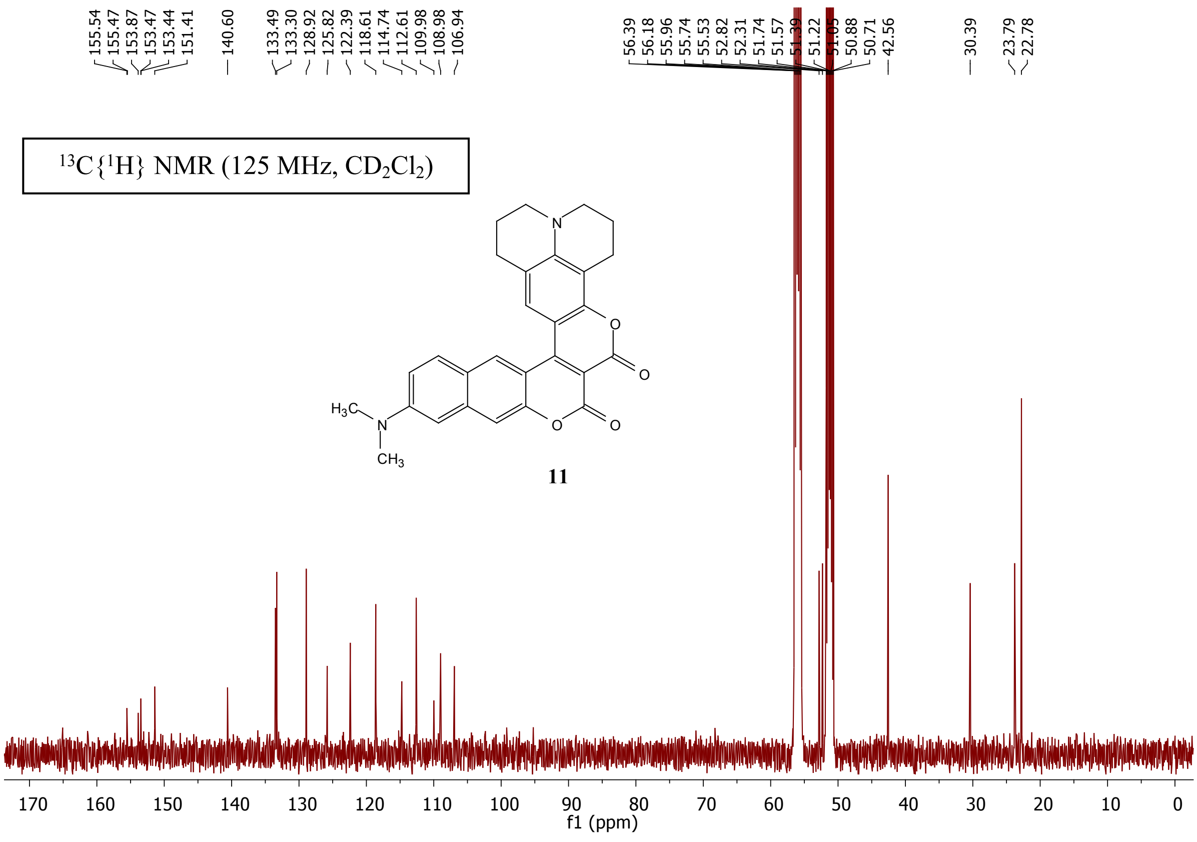
155.54
155.47
153.87
153.47
153.44
151.41
140.60
133.49
133.30
128.92
125.82
122.39
118.61
114.74
112.61
109.98
108.98
106.94
56.39
56.18
55.96
55.74
55.53
52.82
52.31
51.74
51.57
51.39
51.22
51.05
50.88
50.71
42.56
30.39
23.79
22.78
13C{1H} NMR (125 MHz, CD2Cl2)
N
O
O
O
O
H3C
N
CH3
11
170 160 150 140 130 120 110 100 90 80 70 60 50 40 30 20 10 0
f1 (ppm)

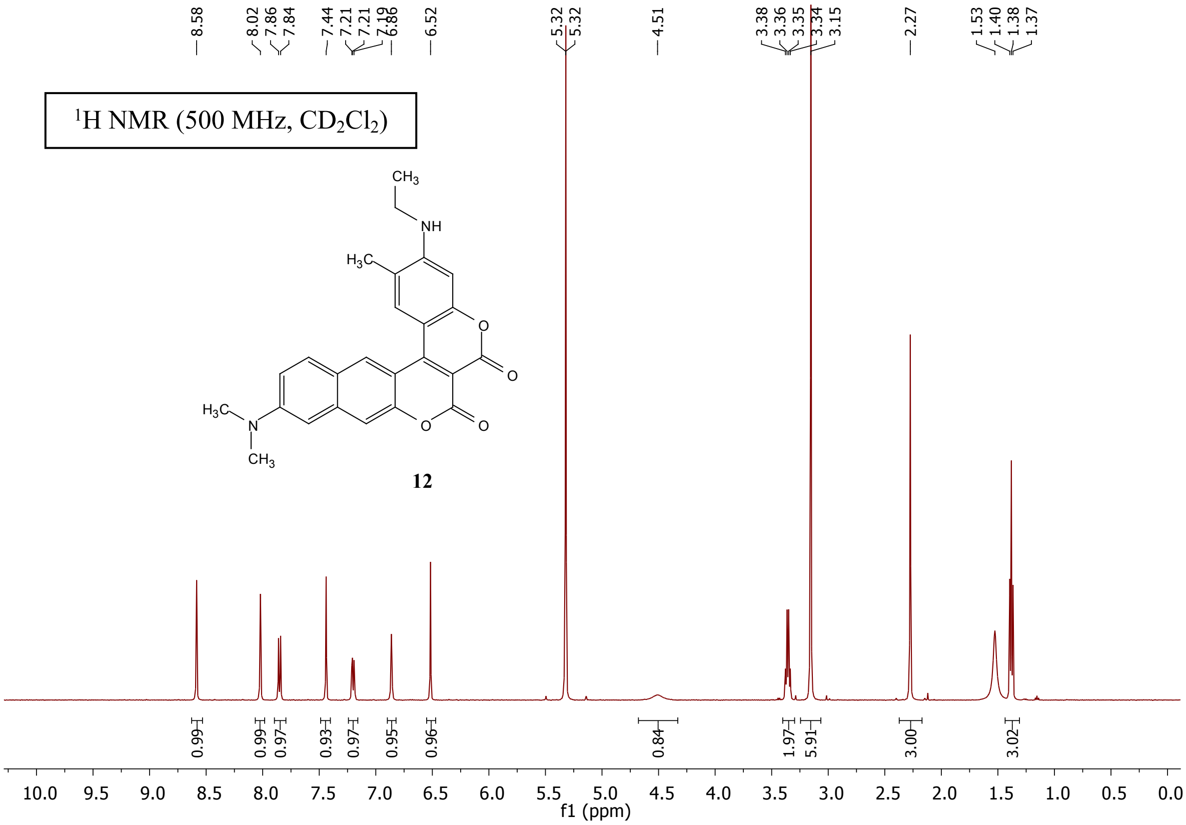

8.58
8.02
7.86
7.84
7.44
7.21
7.21
7.19
6.86
6.52
5.32
5.32
4.51
3.38
3.36
3.35
3.34
3.15
2.27
1.53
1.40
1.38
1.37
^{1}H NMR (500 MHz, CD_2Cl_2)
12
0.99
0.99
0.97
0.93
0.97
0.95
0.96
0.84
1.97
5.91
3.00
3.02
f1 (ppm)

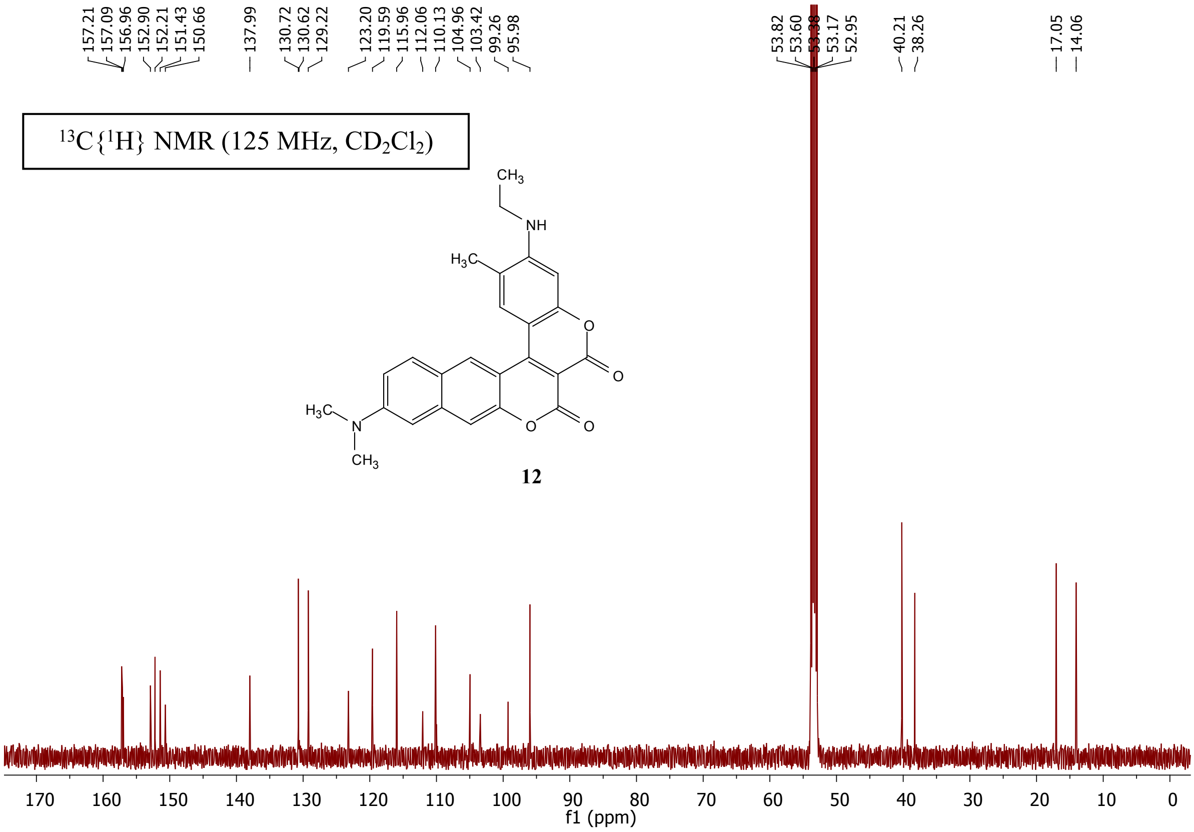

157.21
157.09
156.96
152.90
152.21
151.43
150.66
137.99
130.72
130.62
129.22
123.20
119.59
115.96
112.06
110.13
104.96
103.42
99.26
95.98
53.82
53.60
53.17
52.95
40.21
38.26
17.05
14.06
^{13}C{^{1}H} NMR (125 MHz, CD_2Cl_2)
12
f1 (ppm)

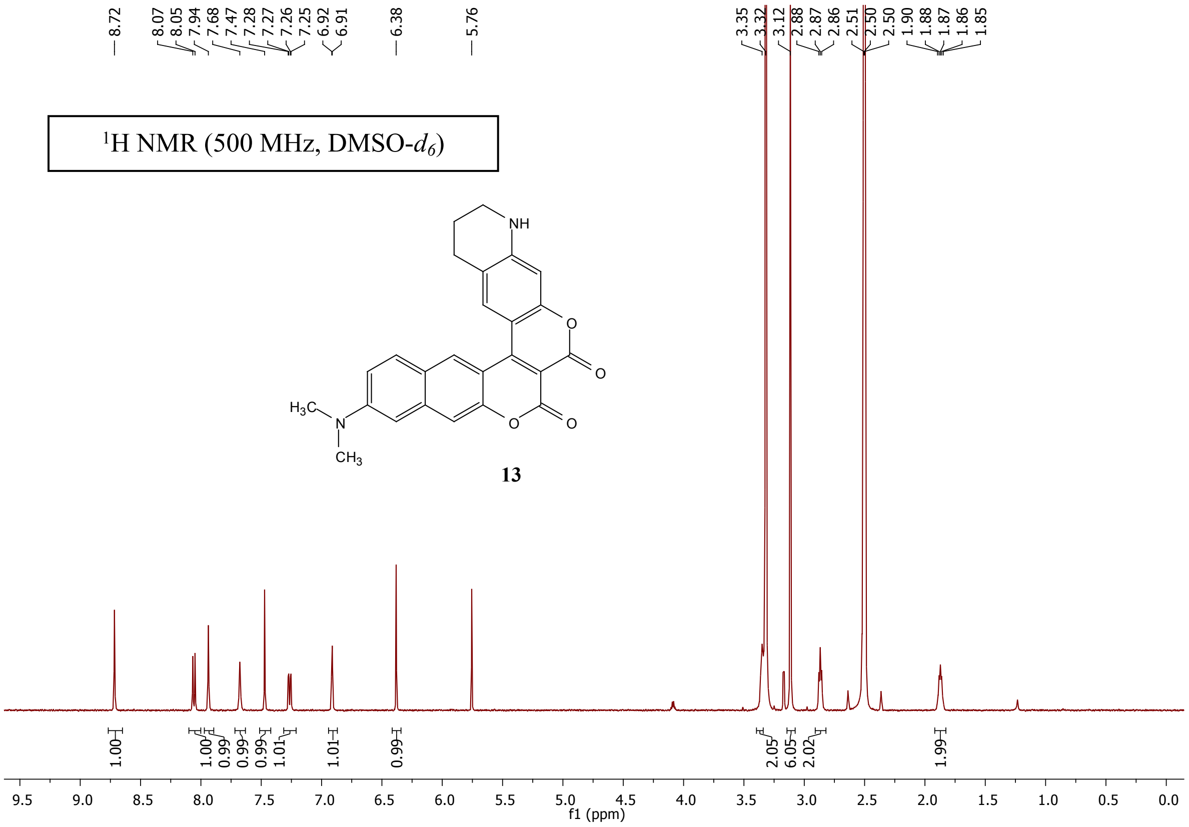

8.72
8.07
8.05
7.94
7.68
7.47
7.28
7.27
7.26
7.25
6.92
6.91
6.38
5.76
3.35
3.32
3.12
2.88
2.87
2.86
2.51
2.50
2.50
1.90
1.88
1.87
1.86
1.85
1H NMR (500 MHz, DMSO-d6)
NH
O
O
O
O
H3C
N
CH3
13
1.00
1.00
0.99
0.99
0.99
1.01
1.01
0.99
2.05
6.05
2.02
1.99
f1 (ppm)

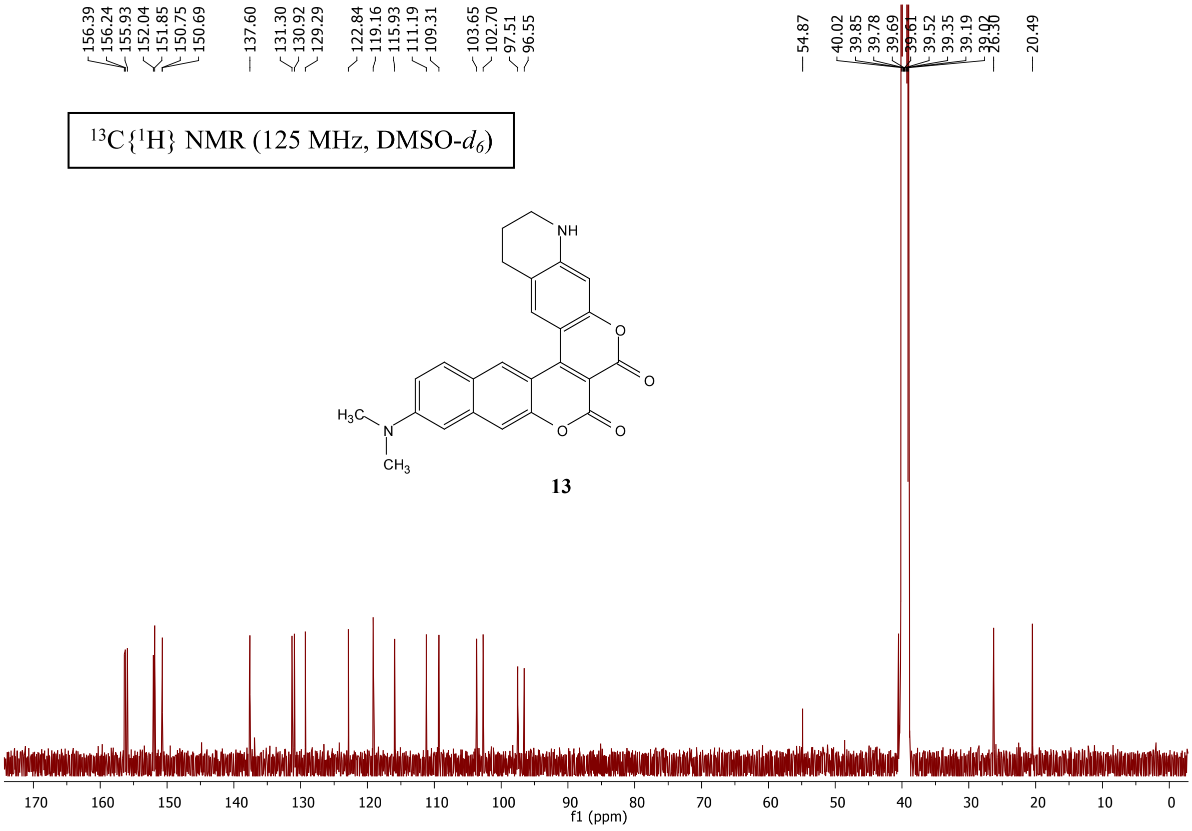

156.39
156.24
155.93
152.04
151.85
150.75
150.69
137.60
131.30
130.92
129.29
122.84
119.16
115.93
111.19
109.31
103.65
102.70
97.51
96.55
54.87
40.02
39.85
39.78
39.69
39.61
39.52
39.35
39.19
26.30
20.49
13C{1H} NMR (125 MHz, DMSO-d6)
NH
O
O
O
O
H3C
N
CH3
13
f1 (ppm)

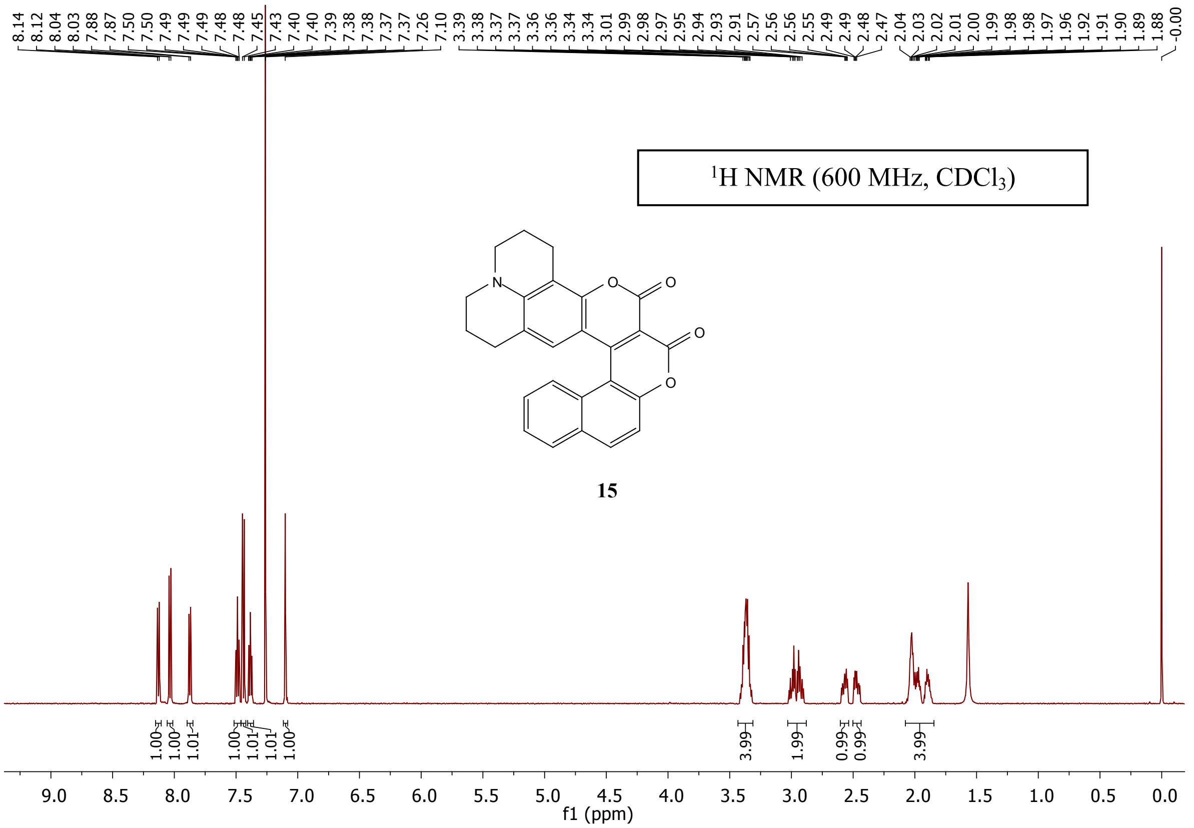

8.14 8.12 8.04 8.03 7.88 7.87 7.50 7.49 7.49 7.49 7.48 7.45 7.43 7.40 7.39 7.38 7.38 7.37 7.37 7.26 7.10 3.39 3.38 3.37 3.37 3.36 3.36 3.34 3.01 2.99 2.98 2.97 2.95 2.94 2.93 2.91 2.57 2.56 2.56 2.55 2.49 2.49 2.48 2.47 2.04 2.03 2.02 2.01 2.00 1.99 1.98 1.98 1.97 1.96 1.92 1.91 1.90 1.89 1.88 -0.00
^{1}H NMR (600 MHz, $CDCl_3$)
N
O
O
O
O
15
1.00 1.00 1.01 1.00 1.01 1.01 1.00 3.99 1.99 0.99 0.99 3.99
9.0 8.5 8.0 7.5 7.0 6.5 6.0 5.5 5.0 4.5 4.0 3.5 3.0 2.5 2.0 1.5 1.0 0.5 0.0
f1 (ppm)

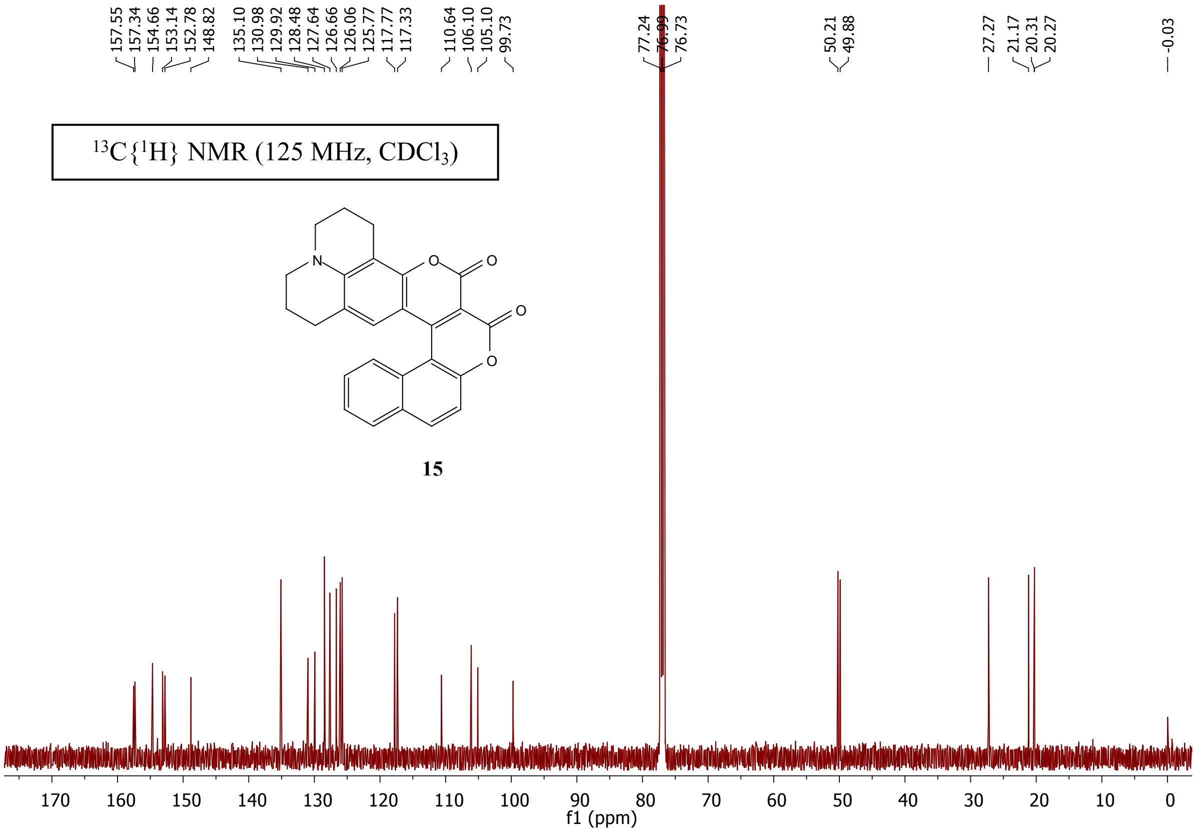

157.55 157.34 154.66 153.14 152.78 148.82 135.10 130.98 129.92 128.48 127.64 126.66 126.06 125.77 117.77 117.33 110.64 106.10 105.10 99.73 77.24 76.99 76.73 50.21 49.88 27.27 21.17 20.31 20.27 -0.03
$^{13}C\{^{1}H\}$ NMR (125 MHz, $CDCl_3$)
N
O
O
O
O
15
170 160 150 140 130 120 110 100 90 80 70 60 50 40 30 20 10 0
f1 (ppm)

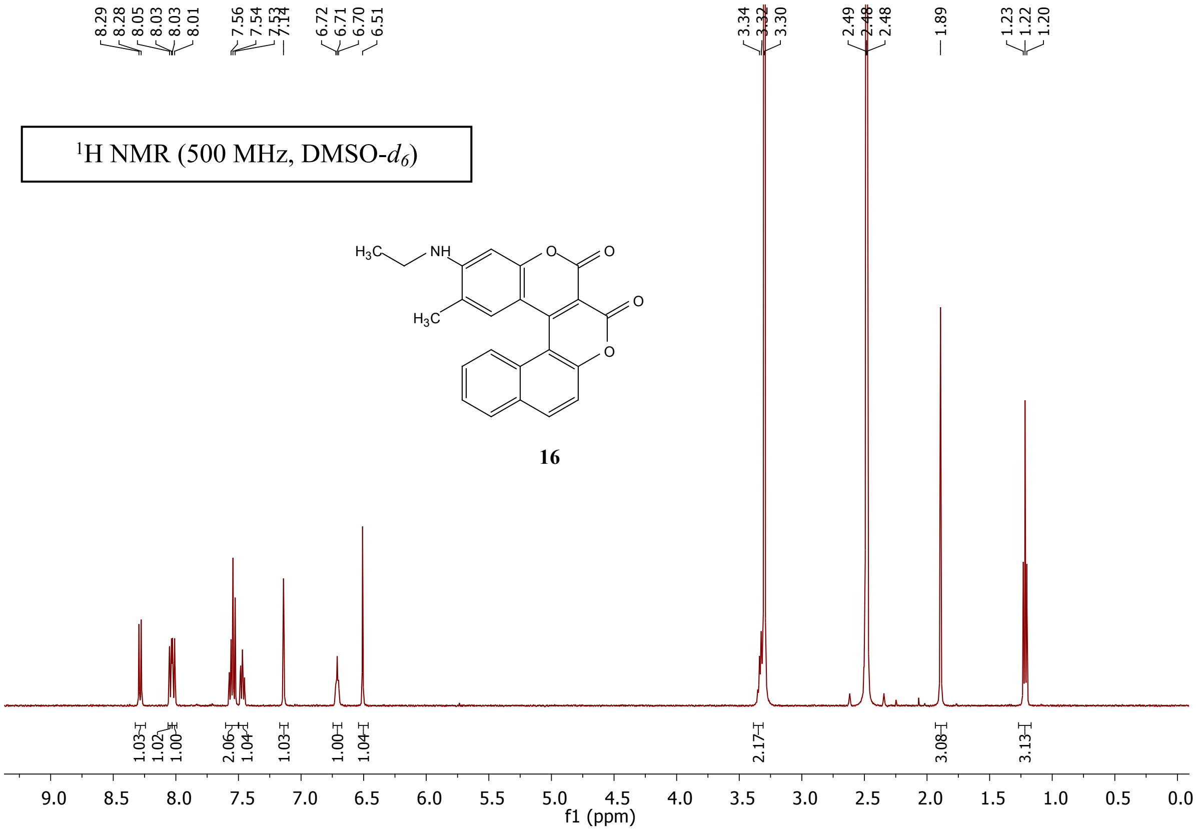

1H NMR (500 MHz, DMSO-d6)
16
f1 (ppm)

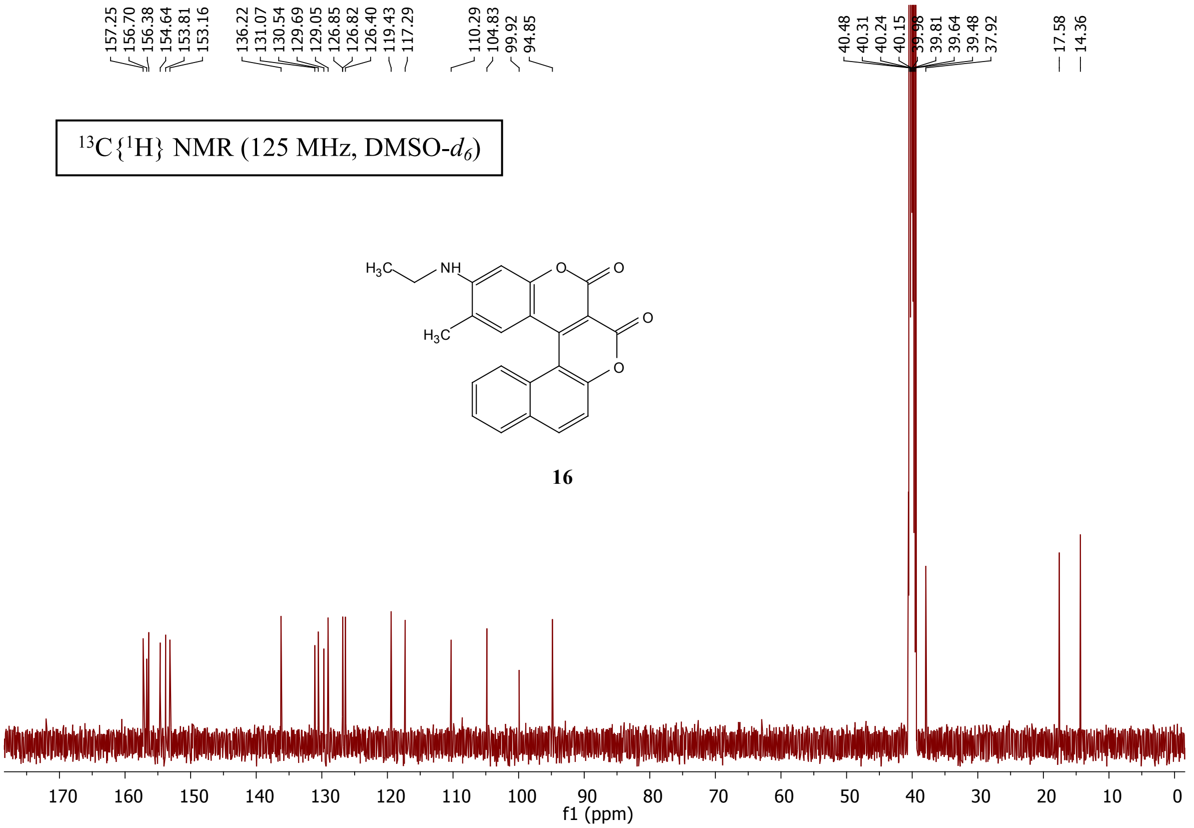

13C{1H} NMR (125 MHz, DMSO-d6)
16
f1 (ppm)

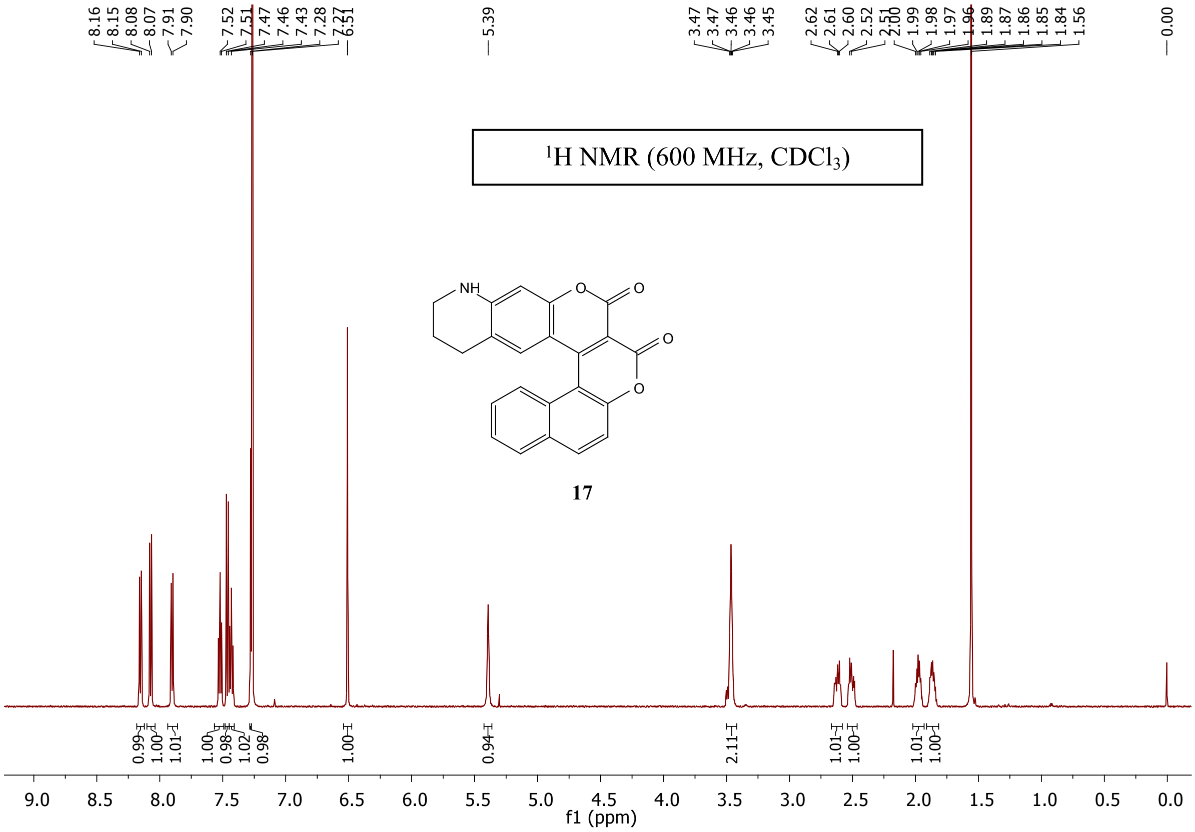
1H NMR (600 MHz, CDCl3)
17
f1 (ppm)

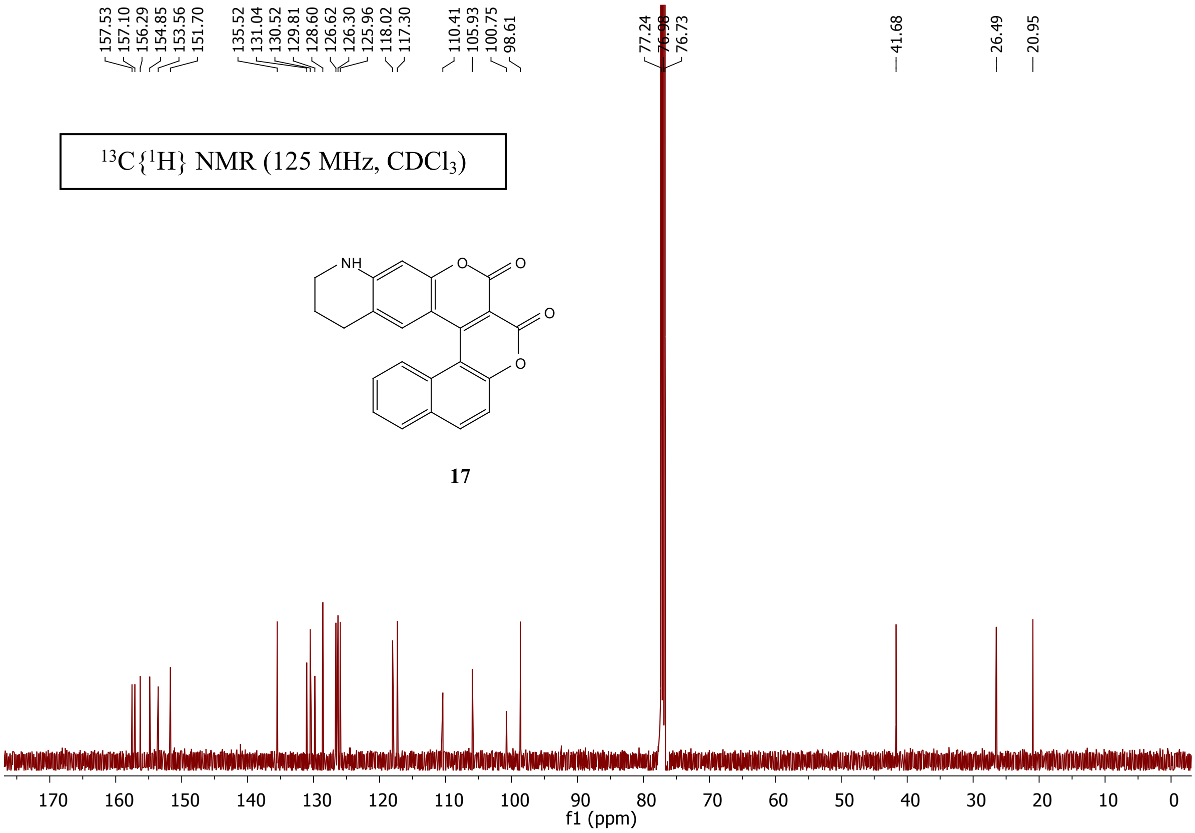
13C{1H} NMR (125 MHz, CDCl3)
17
f1 (ppm)

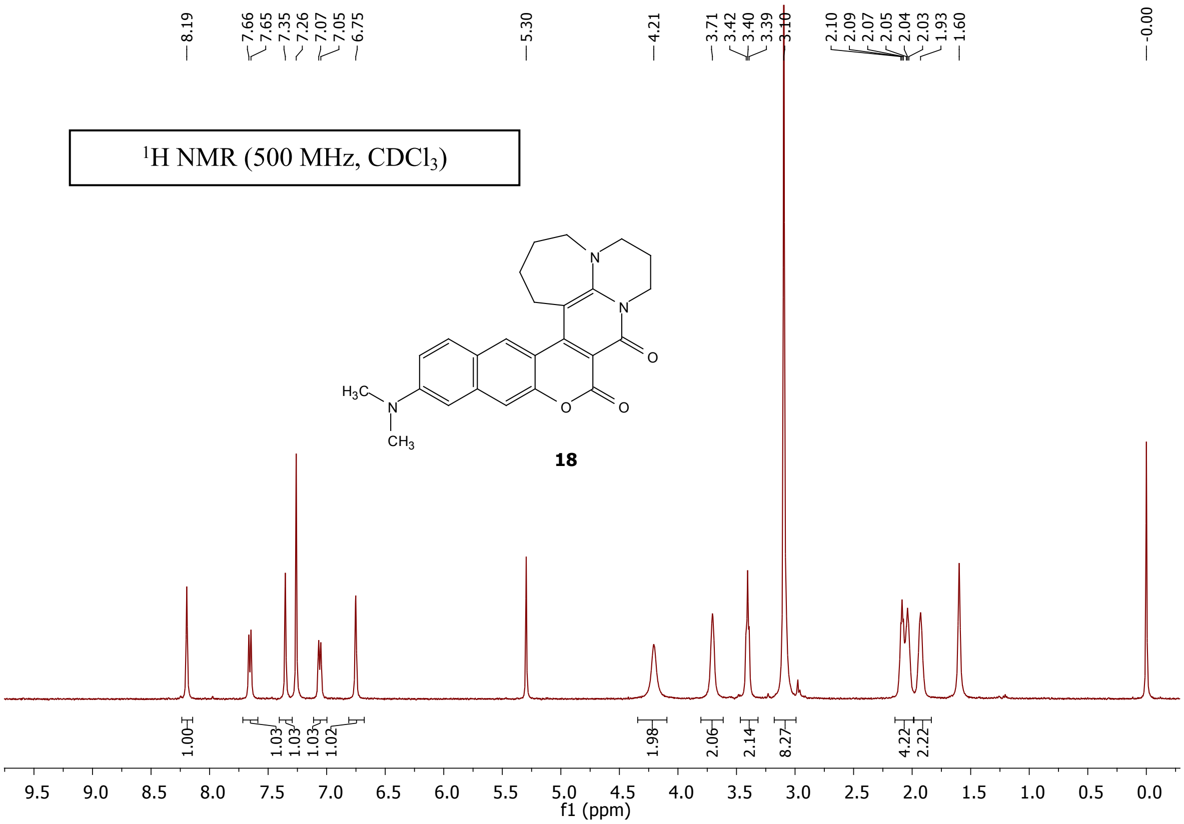
8.19
7.66
7.65
7.35
7.26
7.07
7.05
6.75
5.30
4.21
3.71
3.42
3.40
3.39
2.10
2.09
2.07
2.05
2.04
2.03
1.93
1.60
-0.00
^{1}H NMR (500 MHz, CDCl$_3$)
H$_3$C
N
CH$_3$
O
N
N
O
O
18
1.00
1.03
1.03
1.03
1.02
1.98
2.06
2.14
8.27
4.22
2.22
9.5 9.0 8.5 8.0 7.5 7.0 6.5 6.0 5.5 5.0 4.5 4.0 3.5 3.0 2.5 2.0 1.5 1.0 0.5 0.0
f1 (ppm)

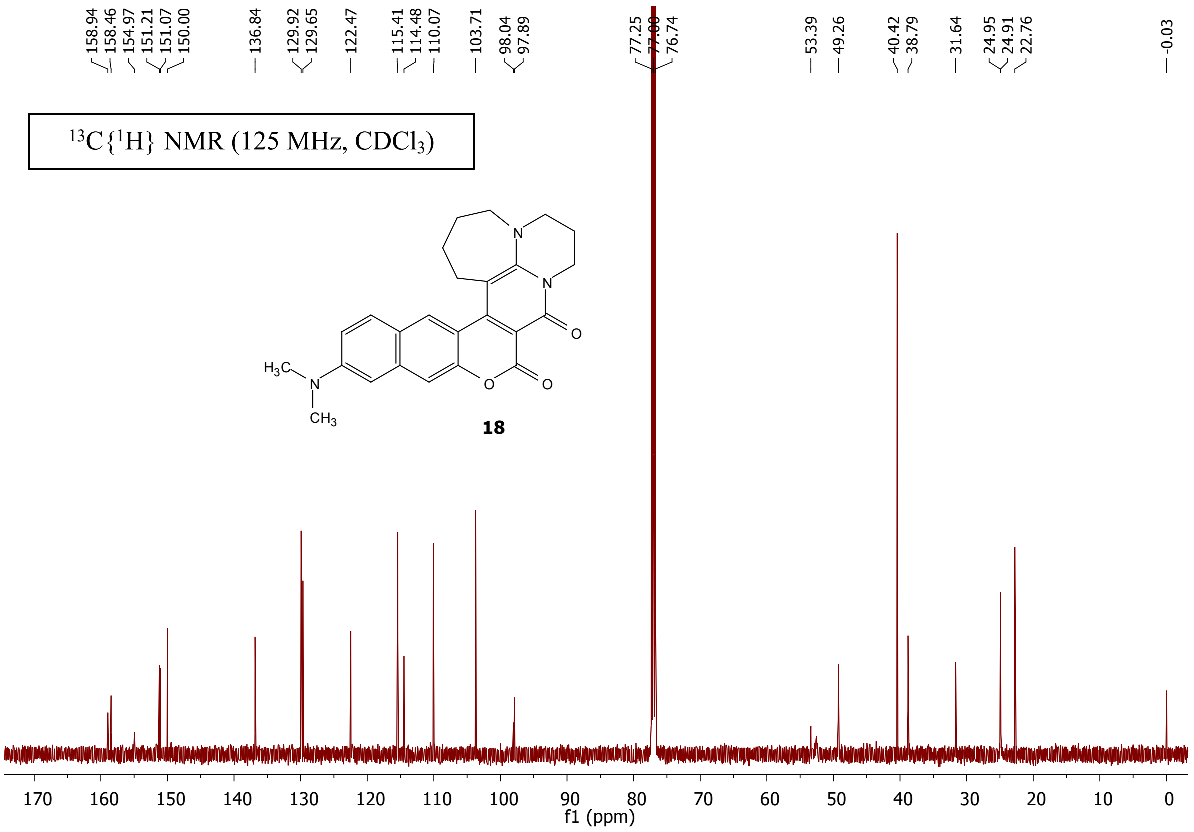
158.94
158.46
154.97
151.21
151.07
150.00
136.84
129.92
129.65
122.47
115.41
114.48
110.07
103.71
98.04
97.89
77.25
76.74
53.39
49.26
40.42
38.79
31.64
24.95
24.91
22.76
-0.03
^{13}C{^{1}H} NMR (125 MHz, CDCl$_3$)
H$_3$C
N
CH$_3$
O
N
N
O
O
18
170 160 150 140 130 120 110 100 90 80 70 60 50 40 30 20 10 0
f1 (ppm)

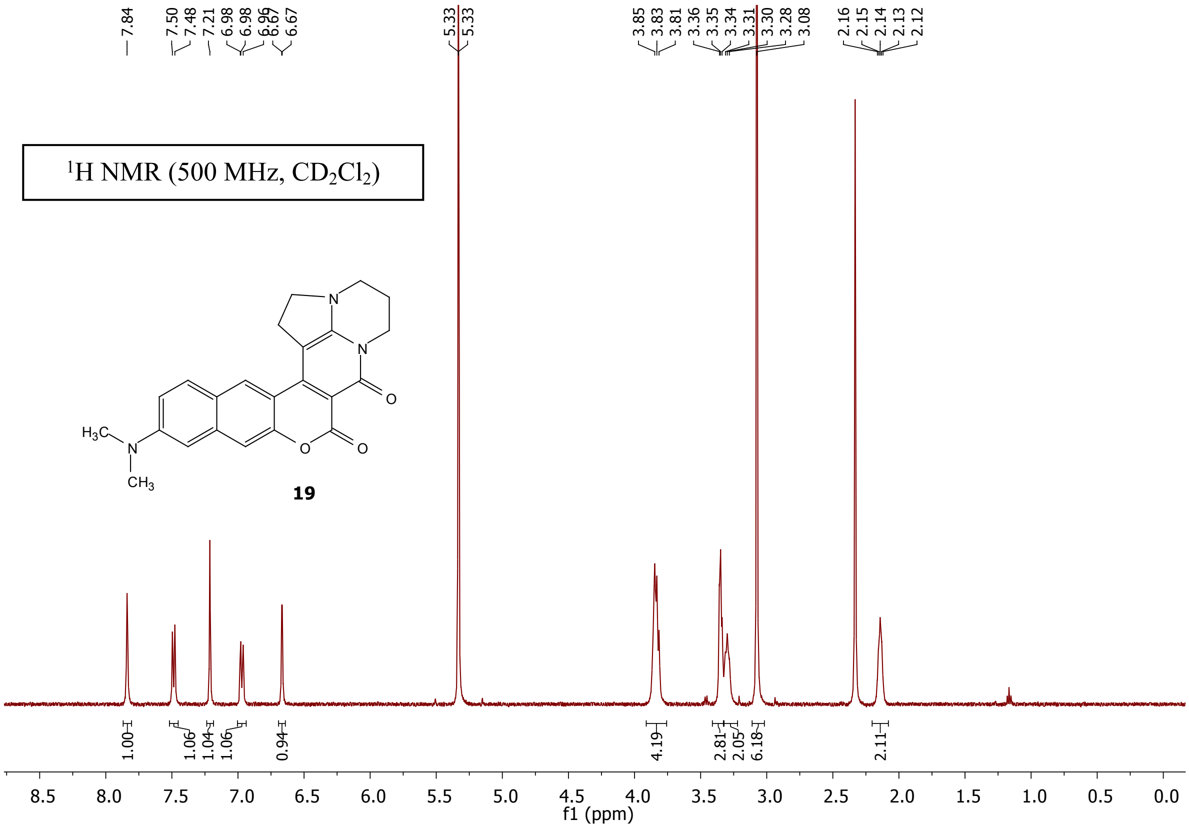

^1H NMR (500 MHz, CD_2Cl_2)
19
f1 (ppm)

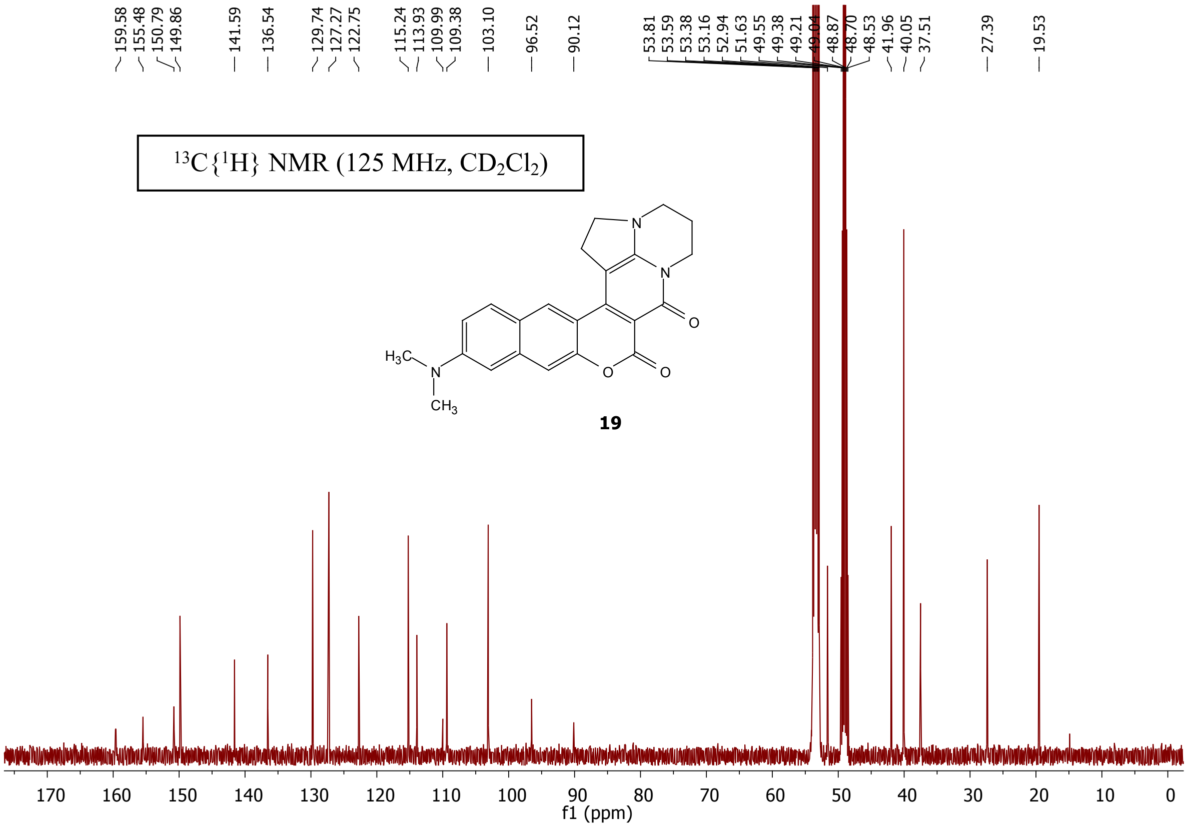

$^{13}C\{^1H\}$ NMR (125 MHz, CD_2Cl_2)
19
f1 (ppm)

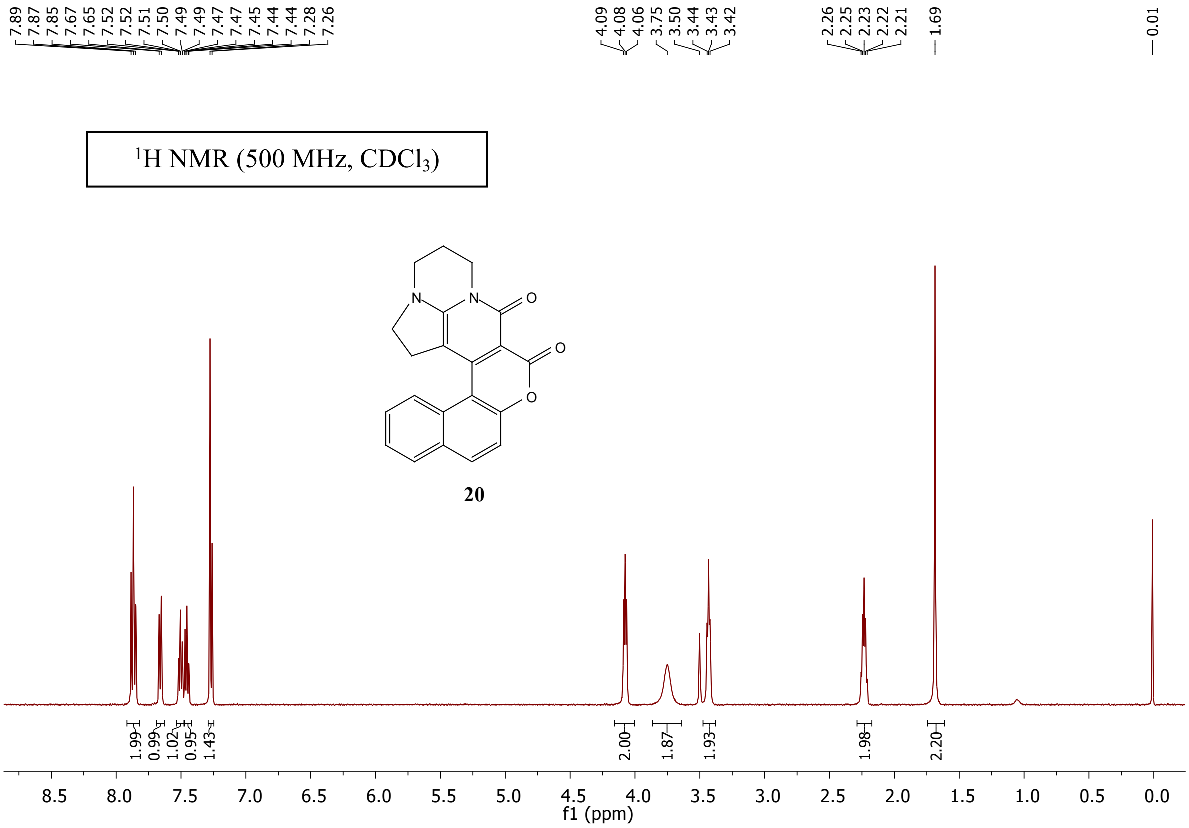
7.89 7.87 7.85 7.67 7.65 7.52 7.51 7.50 7.49 7.49 7.47 7.45 7.44 7.44 7.28 7.26
4.09 4.08 4.06 3.75 3.50 3.44 3.43 3.42
2.26 2.25 2.23 2.22 2.21
1.69
0.01
^{1}H NMR (500 MHz, CDCl$_3$)
N
N
O
O
O
20
1.99 0.99 1.02 0.95 1.43
2.00 1.87 1.93
1.98 2.20
8.5 8.0 7.5 7.0 6.5 6.0 5.5 5.0 4.5 4.0 3.5 3.0 2.5 2.0 1.5 1.0 0.5 0.0
f1 (ppm)

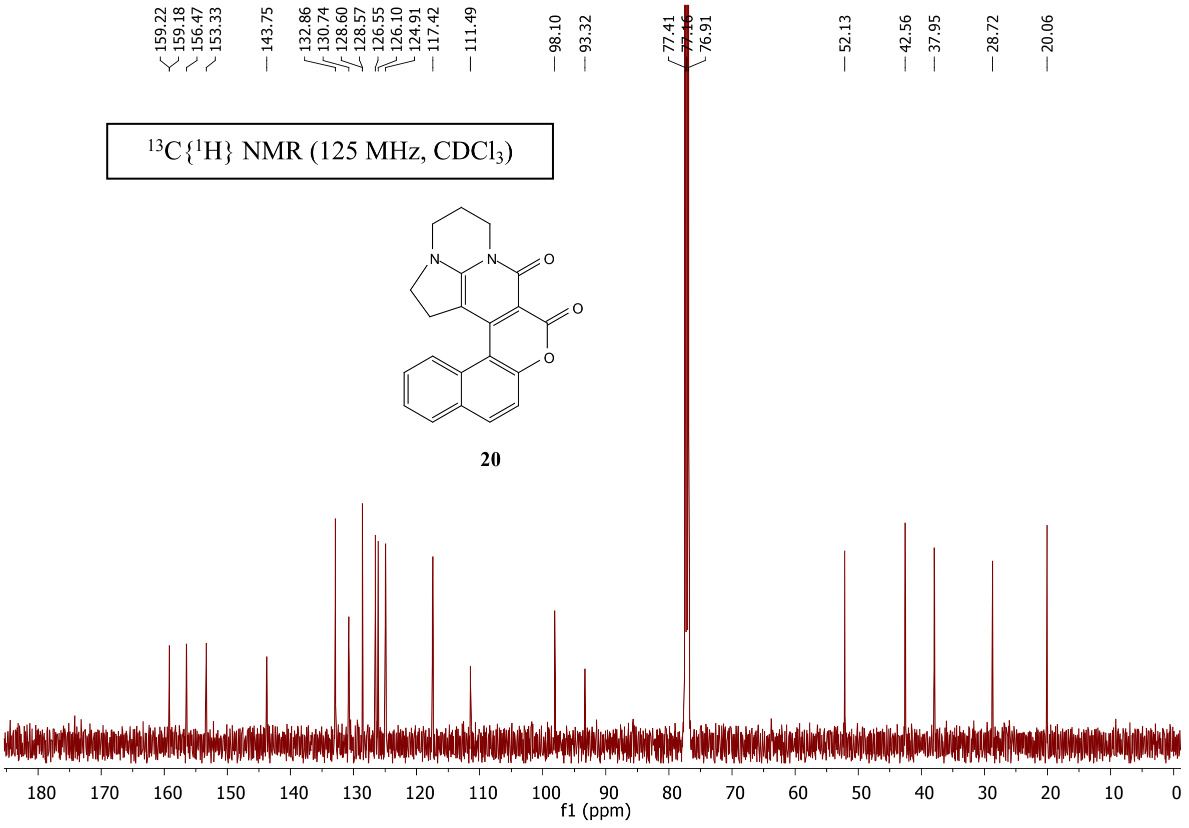
159.22 159.18 156.47 153.33
143.75
132.86 130.74 128.60 128.57 126.55 126.10 124.91 117.42
111.49
98.10
93.32
77.41 77.16 76.91
52.13
42.56
37.95
28.72
20.06
^{13}C{^{1}H} NMR (125 MHz, CDCl$_3$)
N
N
O
O
O
20
180 170 160 150 140 130 120 110 100 90 80 70 60 50 40 30 20 10 0
f1 (ppm)

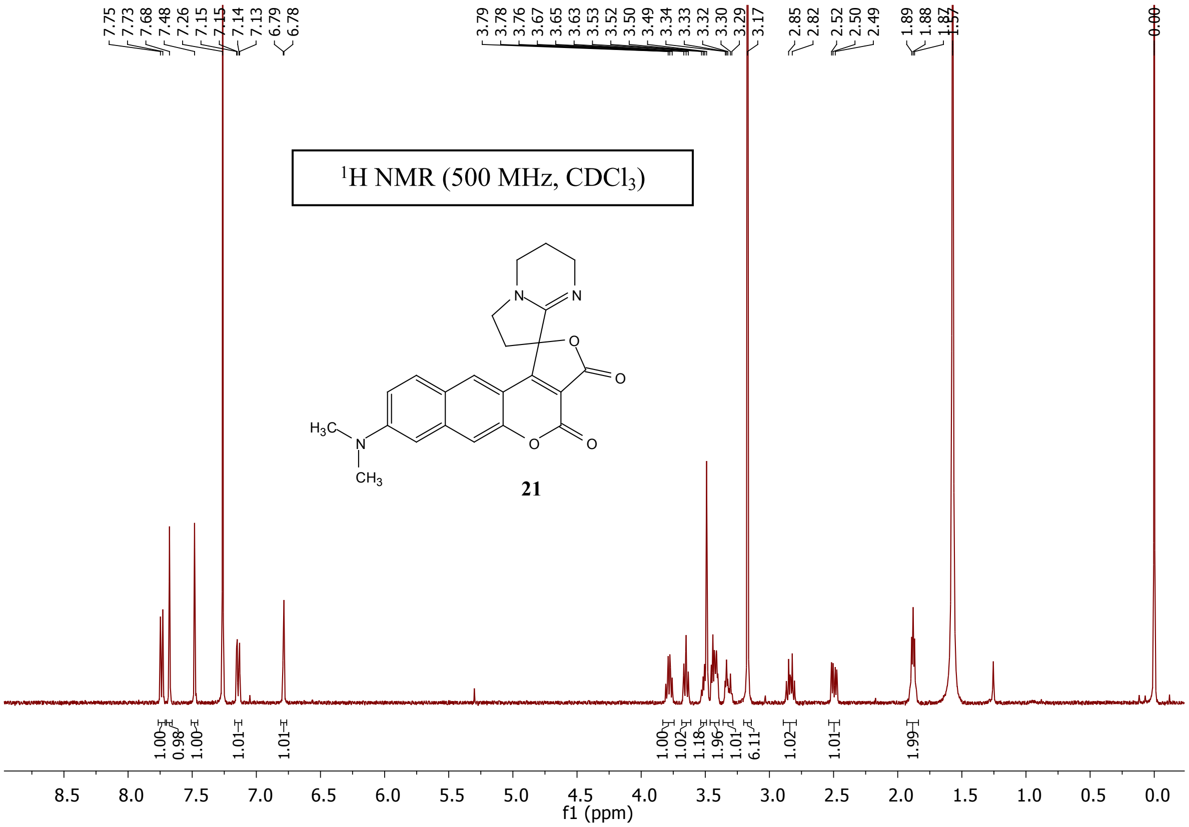

7.75
7.73
7.68
7.48
7.26
7.15
7.15
7.14
7.13
6.79
6.78
3.79
3.78
3.76
3.67
3.65
3.63
3.53
3.52
3.50
3.49
3.34
3.33
3.32
3.30
3.29
3.17
2.85
2.82
2.52
2.50
2.49
1.89
1.88
1.87
1.57
0.00
^{1}H NMR (500 MHz, $CDCl_3$)
N
N
O
O
O
O
H_3C
N
CH_3
21
1.00
0.98
1.00
1.01
1.01
1.00
1.02
1.18
1.96
1.01
6.11
1.02
1.01
1.99
f1 (ppm)

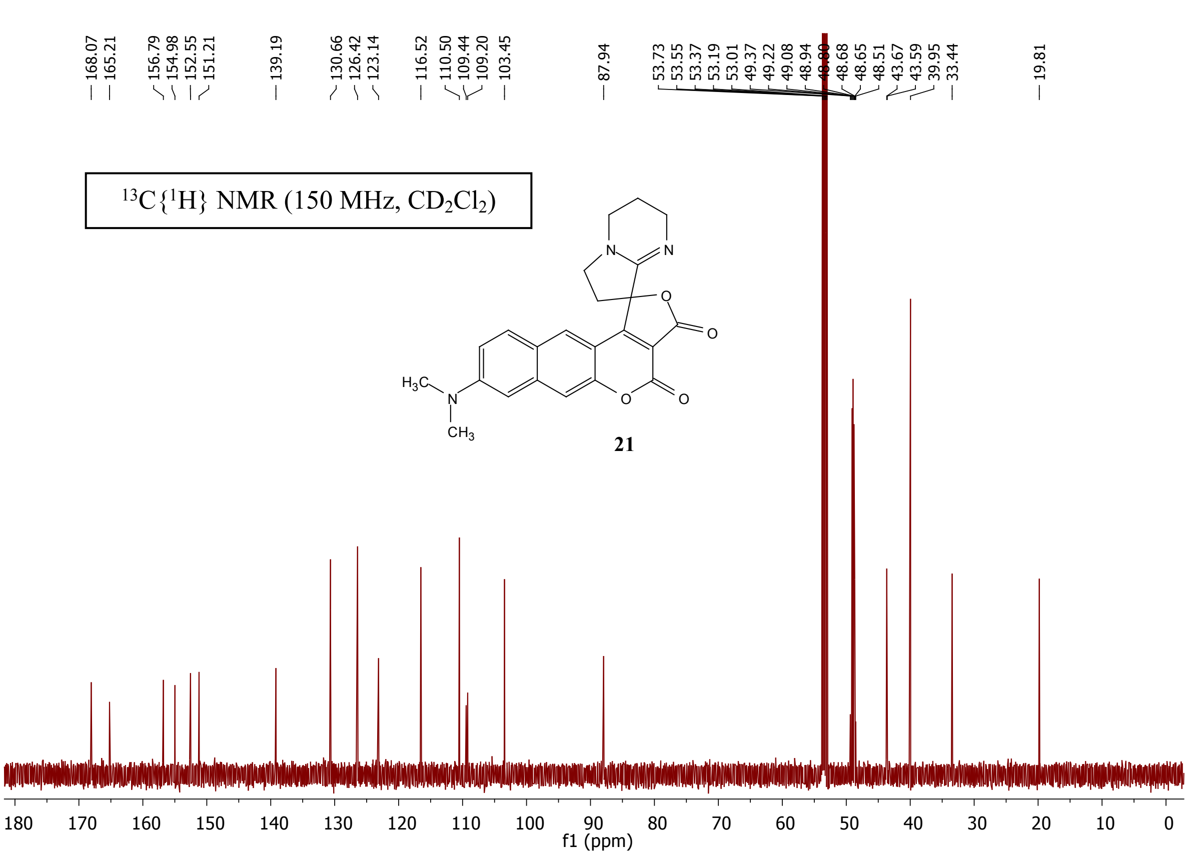

168.07
165.21
156.79
154.98
152.55
151.21
139.19
130.66
126.42
123.14
116.52
110.50
109.44
109.20
103.45
87.94
53.73
53.55
53.37
53.19
53.01
49.37
49.22
49.08
48.94
48.80
48.68
48.65
48.51
43.67
43.59
39.95
33.44
19.81
$^{13}C\{^{1}H\}$ NMR (150 MHz, CD_2Cl_2)
N
N
O
O
O
O
H_3C
N
CH_3
21
f1 (ppm)

6. HRMS Spectra.

Elements Used:
C: 0-200 H: 0-200 N: 0-2 O: 0-4 Na: 0-1

Mass	Calc. Mass	mDa	PPM	DBE	Formula	i-FIT	i-FIT Norm	Fit Conf %	C	H	N	O	Na
429.1781	429.1790	-0.9	-2.1	12.5	C24 H26 N2 O4 Na	562.9	n/a	n/a	24	26	2	4	1

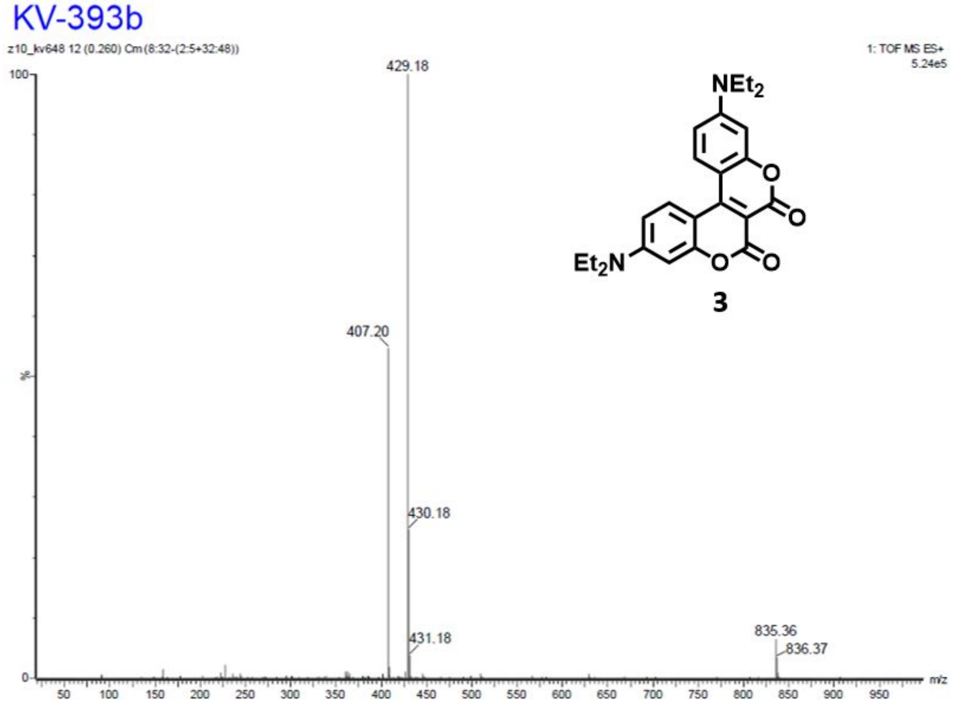

Elements Used:
C: 0-150 H: 0-150 N: 0-3 O: 0-4

Mass	Calc. Mass	mDa	PPM	DBE	Formula	i-FIT	i-FIT Norm	Fit Conf %	C	H	N	O
431.1966	431.1971	-0.5	-1.2	14.5	C26 H27 N2 O4	542.9	n/a	n/a	26	27	2	4

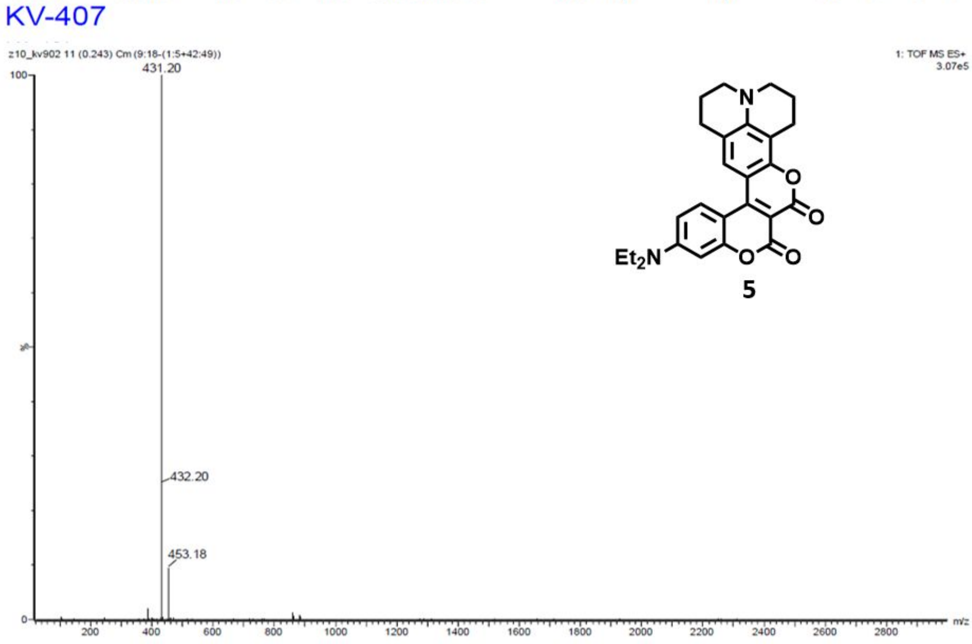

Elemental Composition Report

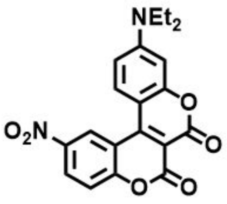

7

Single Mass Analysis
Tolerance = 10.0 PPM / DBE: min = -1.5, max = 50.0
Selected filters: None

Monoisotopic Mass, Odd and Even Electron Ions
16 formula(e) evaluated with 1 results within limits (up to 50 best isotopic matches for each mass)
Elements Used:
C: 0-70 H: 0-100 N: 0-3 O: 6-6

L. Kielesinski
LK 711
z10_lk0121h 55 (2.097) Cm (48:70)
AUTOSPEC
26-Jan-2021 11:19:37
Operator: Malgorzata Grela
Voltage EI+
1.83e4

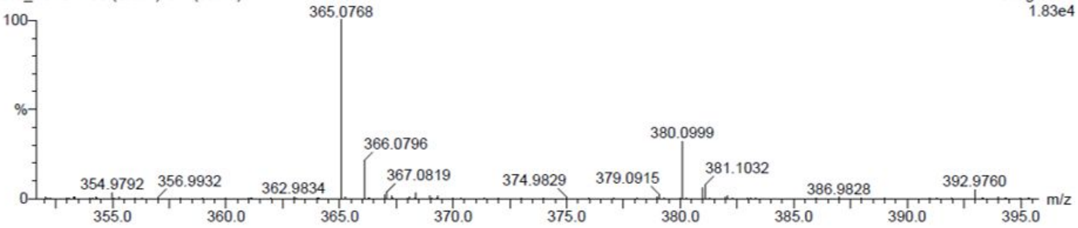

Mass	Calc. Mass	mDa	PPM	DBE	i-FIT	Formula
Minimum:				-1.5		
Maximum:		5.0	10.0	50.0		
380.0999	380.1008	-0.9	-2.4	14.0	3.0	C20 H16 N2 O6

Elemental Composition Report

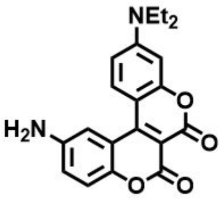

8

Single Mass Analysis
Tolerance = 15.0 PPM / DBE: min = -1.5, max = 50.0
Selected filters: None

Monoisotopic Mass, Odd and Even Electron Ions
23 formula(e) evaluated with 1 results within limits (up to 50 best isotopic matches for each mass)
Elements Used:
C: 0-70 H: 0-100 N: 2-4 O: 4-5

L. Kielesinski
LKI0725-10
z10_lk0463h 165 (6.293) Cm (150:165)
AUTOSPEC
18-Mar-2021 17:50:41
Operator: Marian Olejnik
Voltage EI+
615

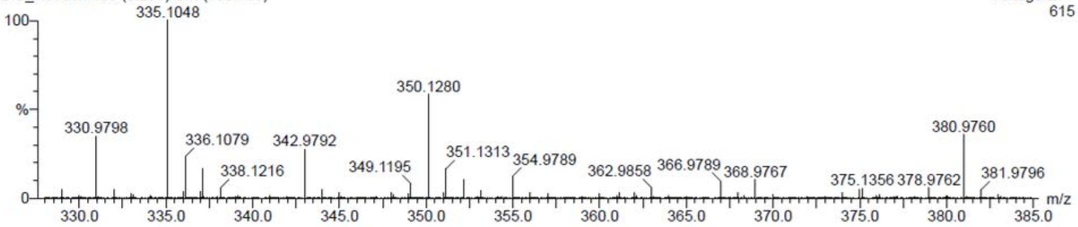

Mass	Calc. Mass	mDa	PPM	DBE	i-FIT	Formula
Minimum:				-1.5		
Maximum:		5.0	15.0	50.0		
350.1280	350.1267	1.3	3.7	13.0	22.3	C20 H18 N2 O4

Elemental Composition Report

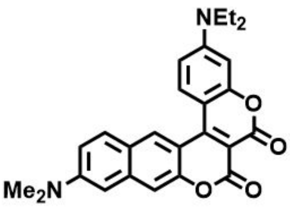

10

Single Mass Analysis
Tolerance = 10.0 mDa / DBE: min = -1.5, max = 50.0
Selected filters: None

Monoisotopic Mass, Odd and Even Electron Ions
28 formula(e) evaluated with 1 results within limits (up to 50 closest results for each mass)
Elements Used:
C: 0-70 H: 0-100 N: 2-2 O: 0-4

L. Kielesinski
LK572
z10_lk1359h 147 (5.607) Cm (145:167)
AUTOSPEC
30-May-2019 11:06:36
operator: Malgorzata Grela
Voltage EI+
1.08e3

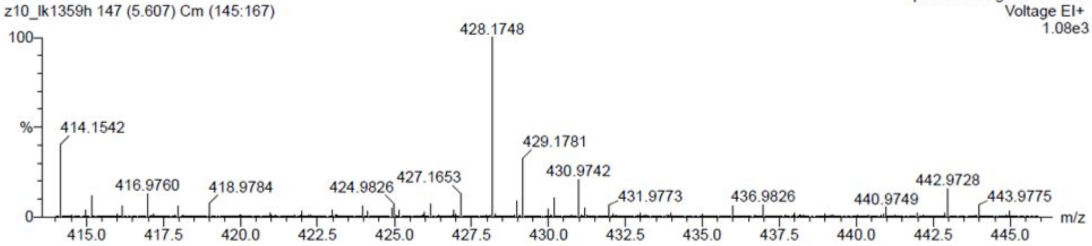

Minimum:				-1.5		
Maximum:		10.0	20.0	50.0		
Mass	Calc. Mass	mDa	PPM	DBE	i-FIT	Formula
428.1748	428.1736	1.2	2.8	16.0	13.7	C26 H24 N2 O4

Elemental Composition Report

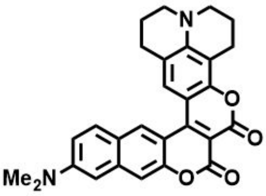

11

Single Mass Analysis
Tolerance = 20.0 PPM / DBE: min = -1.5, max = 50.0
Selected filters: None

Monoisotopic Mass, Odd and Even Electron Ions
6 formula(e) evaluated with 1 results within limits (up to 50 best isotopic matches for each mass)
Elements Used:
C: 0-70 H: 0-100 N: 2-2 O: 4-4

L. Kielesinski
LKI0751-10
z10_lk0934h 172 (6.560) Cm (170:177)
AUTOSPEC
19-May-2021 16:21:06
Operator: Marian Olejnik
Voltage EI+
123

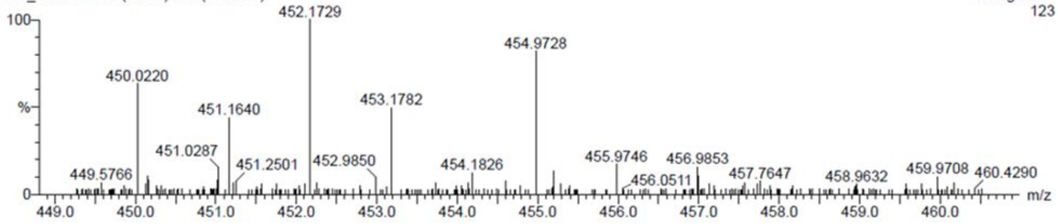

Minimum:				-1.5		
Maximum:		5.0	20.0	50.0		
Mass	Calc. Mass	mDa	PPM	DBE	i-FIT	Formula
452.1729	452.1736	-0.7	-1.5	18.0	4.7	C28 H24 N2 O4

Elemental Composition Report

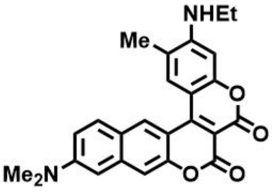

12

Single Mass Analysis

Tolerance = 20.0 PPM / DBE: min = -1.5, max = 50.0
Selected filters: None

Monoisotopic Mass, Odd and Even Electron Ions
36 formula(e) evaluated with 1 results within limits (up to 50 best isotopic matches for each mass)
Elements Used:
C: 0-70 H: 0-100 N: 2-2 O: 0-6

L. Kielesinski
LKI0756-10
z10_lk1298h 72 (2.745) Cm (64:72)

AUTOSPEC

31-May-2021 15:43:41
Operator: Klara Nestorowicz
Voltage EI+
4.68e3

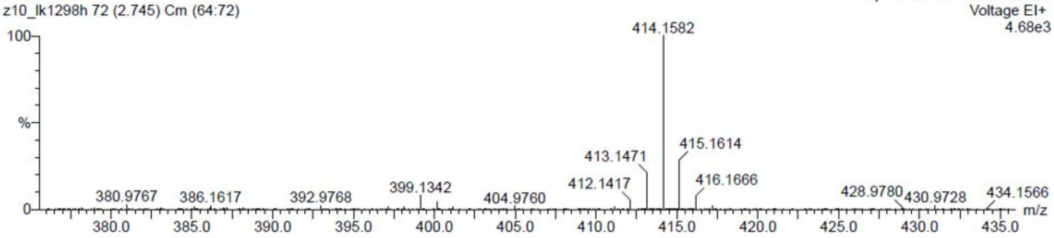

Minimum:				-1.5		
Maximum:		5.0	20.0	50.0		
Mass	Calc. Mass	mDa	PPM	DBE	i-FIT	Formula
414.1582	414.1580	0.2	0.5	16.0	21.5	C25 H22 N2 O4

Single Mass Analysis

Tolerance = 3.0 mDa / DBE: min = -1.5, max = 50.0
Element prediction: Off
Number of isotope peaks used for i-FIT = 3
Monoisotopic Mass, Even Electron Ions
302 formula(e) evaluated with 1 results within limits (up to 100 closest results for each mass)
Elements Used:
C: 0-100 H: 0-200 N: 0-5 O: 0-10 Na: 1-1

Mass	Calc. Mass	mDa	PPM	DBE	Formula	i-FIT	i-FIT Norm	Fit Conf %	C	H	N	O	Na
435.1322	435.1321	0.1	0.2	16.5	C25 H20 N2 O4 Na	78.8	n/a	n/a	25	20	2	4	1

LKI0758-10
z10_lk1668c 13 (0.260) Cm (9:20-(2.7+26:32))

1: TOF MS ES+
1.76e5

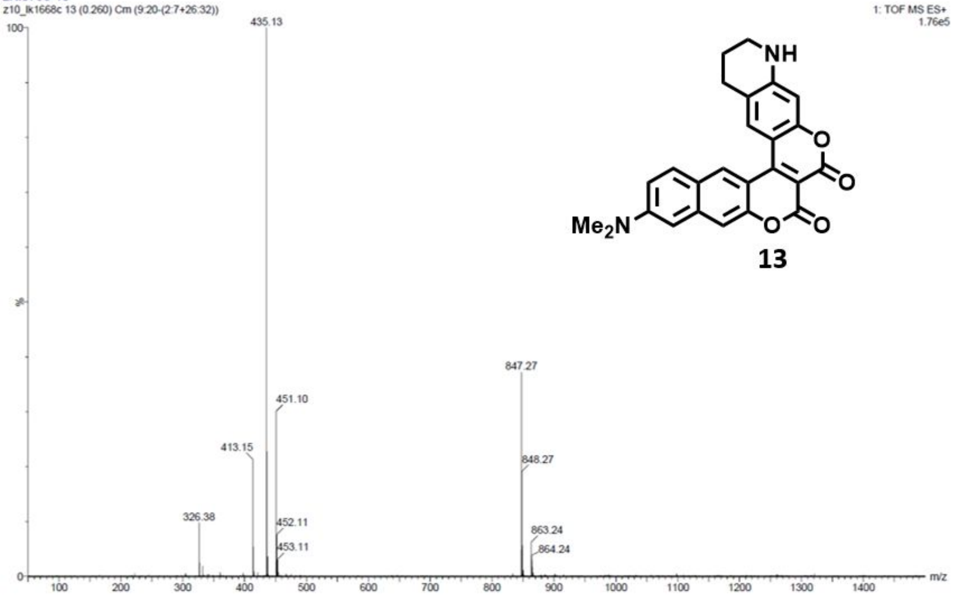

13

Elemental Composition Report

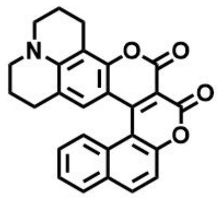

15

Single Mass Analysis
Tolerance = 15.0 PPM / DBE: min = -1.5, max = 50.0
Selected filters: None

Monoisotopic Mass, Odd and Even Electron Ions
49 formula(e) evaluated with 1 results within limits (up to 50 best isotopic matches for each mass)
Elements Used:
C: 0-70 H: 0-100 N: 0-4 O: 4-5

L. Kielesinski
LKI0730-10
z10_lk0524h 300 (11.442) Cm (295:318)

AUTOSPEC

18-Mar-2021 18:47:42
Operator: Marian Olejnik
Voltage EI+
462

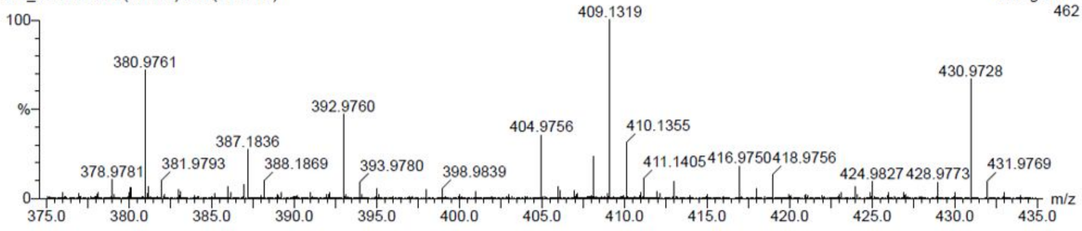

Minimum:				-1.5		
Maximum:		5.0	15.0	50.0		
Mass	Calc. Mass	mDa	PPM	DBE	i-FIT	Formula
409.1319	409.1314	0.5	1.2	18.0	7.0	C26 H19 N O4

Elemental Composition Report

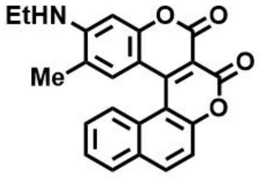

16

Single Mass Analysis
Tolerance = 15.0 PPM / DBE: min = -1.5, max = 50.0
Selected filters: None

Monoisotopic Mass, Odd and Even Electron Ions
49 formula(e) evaluated with 1 results within limits (up to 50 best isotopic matches for each mass)
Elements Used:
C: 0-70 H: 0-100 N: 0-1 O: 0-4

L. Kielesinski
LKI0737-10
z10_lk0698h 535 (20.405) Cm (531:561)

AUTOSPEC

31-Mar-2021 14:20:45
Operator: Marian Olejnik
Voltage EI+
452

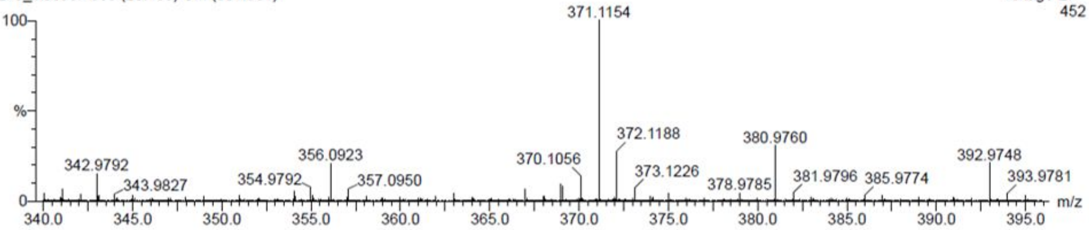

Minimum:				-1.5		
Maximum:		5.0	15.0	50.0		
Mass	Calc. Mass	mDa	PPM	DBE	i-FIT	Formula
371.1154	371.1158	-0.4	-1.1	16.0	2.8	C23 H17 N O4

Elemental Composition Report

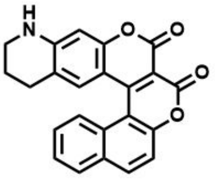

17

Single Mass Analysis

Tolerance = 20.0 PPM / DBE: min = -1.5, max = 50.0
Selected filters: None

Monoisotopic Mass, Odd and Even Electron Ions
15 formula(e) evaluated with 1 results within limits (up to 50 best isotopic matches for each mass)
Elements Used:
C: 0-70 H: 0-100 N: 1-1 O: 2-4

L. Kielesinski
LKI0746-10
z10_lk0908h 198 (7.552) Cm (198:213)

AUTOSPEC

19-May-2021 17:45:02
Operator: Marian Olejnik
Voltage EI+
1.45e3

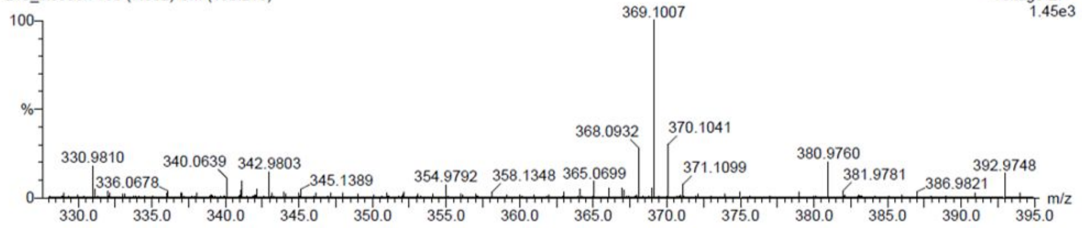

Minimum:				-1.5		
Maximum:		5.0	20.0	50.0		
Mass	Calc. Mass	mDa	PPM	DBE	i-FIT	Formula
369.1007	369.1001	0.6	1.6	17.0	12.2	C23 H15 N O4

Single Mass Analysis

Tolerance = 3.0 mDa / DBE: min = 0.0, max = 100.0
Element prediction: Off
Number of isotope peaks used for i-FIT = 2
Monoisotopic Mass, Even Electron Ions
488 formula(e) evaluated with 3 results within limits (all results (up to 1000) for each mass)
Elements Used:
C: 0-50 H: 0-100 N: 0-6 O: 0-6 Na: 0-1

Mass	Calc. Mass	mDa	PPM	DBE	Formula	i-FIT	i-FIT Norm	Fit Conf %	C	H	N	O	Na
438.1788	438.1794	-0.6	-1.4	14.5	C25 H25 N3 O3 Na	459.2	8.056	0.03	25	25	3	3	1
	438.1777	1.1	2.5	13.5	C22 H24 N5 O5	451.1	0.000	99.97	22	24	5	5	
	438.1818	-3.0	-6.8	17.5	C27 H24 N3 O3	463.6	12.468	0.00	27	24	3	3	

LK 680
z10_lk2084a 10 (0.226) Cm (9:12-(2:7+17:22))

1: TOF MS ES+
1.29e5

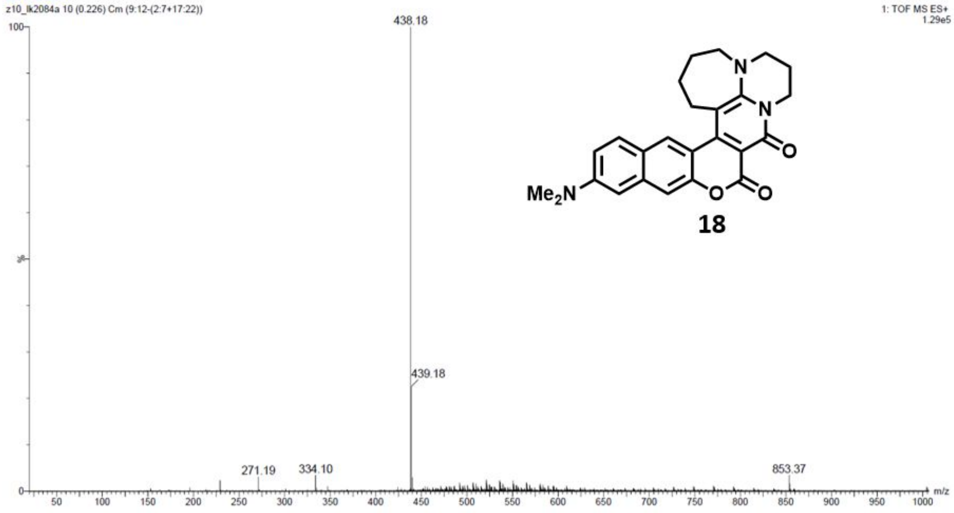

18

Elemental Composition Report

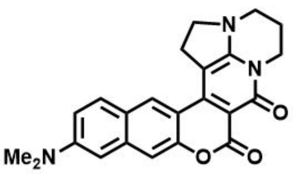

19

Single Mass Analysis
Tolerance = 20.0 PPM / DBE: min = -1.5, max = 50.0
Selected filters: None

Monoisotopic Mass, Odd and Even Electron Ions
24 formula(e) evaluated with 1 results within limits (up to 50 best isotopic matches for each mass)
Elements Used:
C: 0-70 H: 0-100 N: 3-3 O: 0-4

L. Kielesinski
LK 687
z10_lk2401h 50 (1.907) Cm (28:65)

AUTOSPEC

15-Dec-2020 15:46:34
Operator: Malgorzata Grela
Voltage EI+
7.13e3

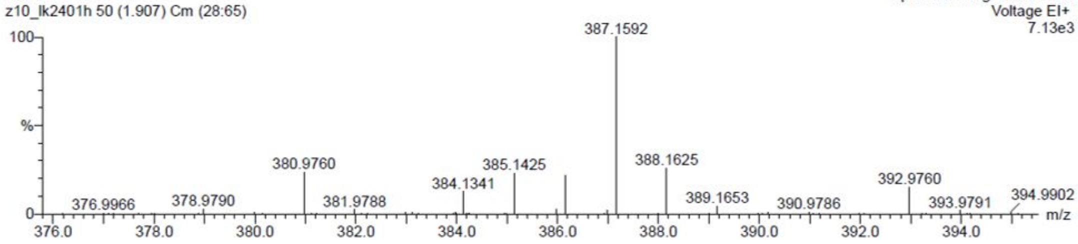

Minimum:				-1.5		
Maximum:		5.0	20.0	50.0		
Mass	Calc. Mass	mDa	PPM	DBE	i-FIT	Formula
387.1592	387.1583	0.9	2.3	15.0	1.9	C23 H21 N3 O3

Single Mass Analysis
Tolerance = 3.0 mDa / DBE: min = -1.5, max = 50.0
Element prediction: Off
Number of isotope peaks used for i-FIT = 3
Monoisotopic Mass, Even Electron Ions
153 formula(e) evaluated with 1 results within limits (up to 100 closest results for each mass)
Elements Used:
C: 0-120 H: 0-200 N: 0-5 O: 0-5 Na: 1-1

Mass	Calc. Mass	mDa	PPM	DBE	Formula	i-FIT	i-FIT Norm	Fit Conf %	C	H	N	O	Na
367.1059	367.1059	0.0	0.0	14.5	C21 H16 N2 O3 Na	76.2	n/a	n/a	21	16	2	3	1

LKI0770-10
z10_lk1763b 25 (0.486) Cm (25:48-2:8)

1: TOF MS ES+
1.05e5

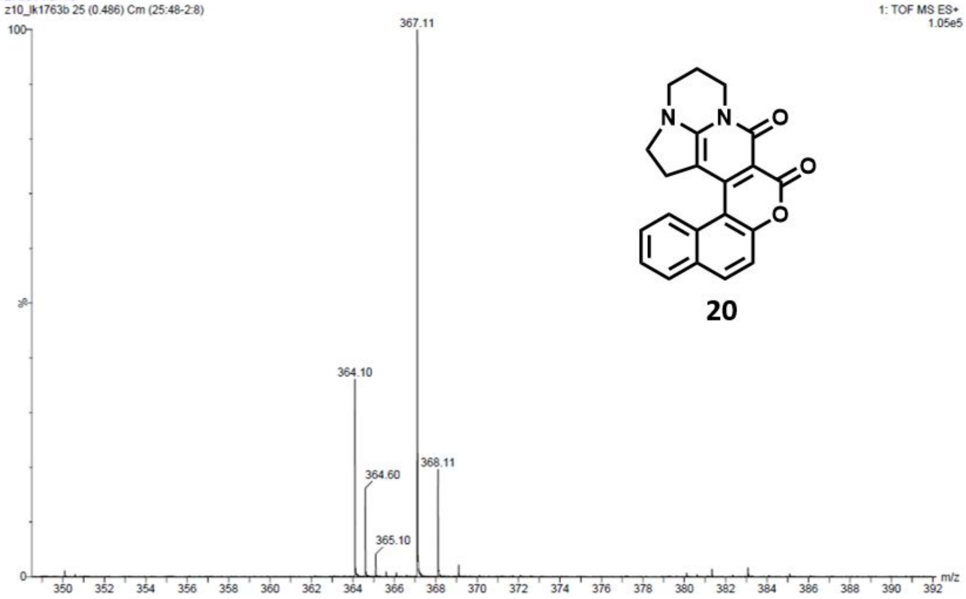

20

Elemental Composition Report

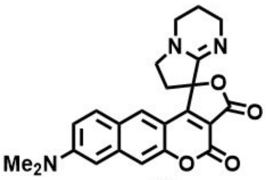

21

Single Mass Analysis
Tolerance = 20.0 PPM / DBE: min = -1.5, max = 80.0
Selected filters: None

Monoisotopic Mass, Odd and Even Electron Ions
14 formula(e) evaluated with 1 results within limits (up to 50 best isotopic matches for each mass)
Elements Used:
C: 0-100 H: 0-200 N: 1-3 O: 4-4

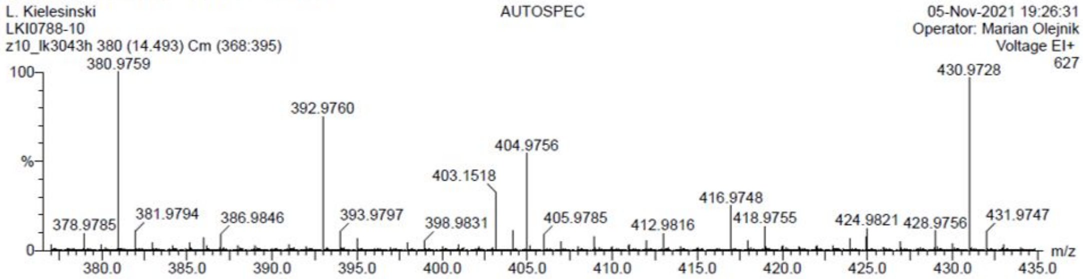

Minimum:				-1.5		
Maximum:		5.0	20.0	80.0		
Mass	Calc. Mass	mDa	PPM	DBE	i-FIT	Formula
403.1518	403.1532	-1.4	-3.5	15.0	2.9	C23 H21 N3 O4
