# Supplementary material for: Polarized, V-Shaped, and Conjoined Biscoumarins: From Lack of Dipole Moment Alignment to High Brightness
Source: J Org Chem. 2022 Apr 12;87(9):5961–75. doi: 10.1021/acs.joc.2c00232 (PMC9087199; doi:10.1021/acs.joc.2c00232)
Supplement: Supplementary file 2 — jo2c00232_si_002.pdf [file jo2c00232_si_002.pdf]

compound 3

S0				S1			
	x	y	z		x	y	z
H	-7.4205	-0.5790	-1.4983	H	-7.5402	-0.4228	-1.2007
H	-6.4436	0.8302	-1.0608	H	-6.5319	0.9438	-0.6944
H	-7.9735	0.5172	-0.2214	H	-7.9855	0.5210	0.2309
H	-5.3550	-4.9279	-0.0359	H	-5.4718	-4.8617	-0.3974
H	-4.1249	-3.9336	0.7670	H	-4.1800	-3.9931	0.4559
H	-5.8191	-3.8159	1.2629	H	-5.8508	-3.8949	1.0376
H	-6.4208	-2.8900	-1.0006	H	-6.5229	-2.7116	-1.0808
H	-4.7584	-3.0018	-1.5172	H	-4.8811	-2.8040	-1.6852
H	-7.0357	-1.5614	0.7891	H	-6.9693	-1.6362	0.9334
H	-6.0718	-0.1787	1.2476	H	-5.9641	-0.2964	1.4529
H	-4.5945	1.0924	0.5136	H	-4.5579	1.0509	0.7188
C	-7.0897	0.0438	-0.6611	C	-7.1425	0.1124	-0.3337
C	-5.1558	-3.9444	0.4019	C	-5.2255	-3.9422	0.1415
C	-6.3600	-0.7967	0.3926	C	-6.3300	-0.8257	0.5712
C	-5.3893	-2.8429	-0.6380	C	-5.4739	-2.7324	-0.7714
C	7.5811	-1.0889	-0.9429	C	7.6114	-1.0972	-0.9191
H	6.4630	0.2361	0.3421	H	6.4979	0.2048	0.3966
H	5.9836	0.2606	-1.3511	H	6.0064	0.2532	-1.2918
H	6.3063	-3.0348	-0.2949	H	6.3982	-3.0184	-0.0451
H	4.6061	-3.3229	-0.1099	H	4.7006	-3.3473	0.0932
C	5.4162	-2.6654	0.2144	C	5.4731	-2.6305	0.3829
H	0.6752	-1.6511	0.5736	H	0.6709	-1.7373	0.4075
N	5.1458	-1.3101	-0.2756	N	5.1861	-1.3429	-0.2536
H	-0.6857	-1.5845	-0.8727	H	-0.7386	-1.5030	-1.1114
C	-0.0585	1.0139	0.0170	C	-0.0213	0.9739	0.0022
O	2.2919	2.4991	-0.3675	O	2.2913	2.4657	-0.4279
C	1.2311	0.3556	-0.0153	C	1.2318	0.3127	-0.0402
C	1.4871	-1.0098	0.2583	C	1.4906	-1.0735	0.1616
C	-1.5251	-1.0145	-0.4984	C	-1.5666	-0.9679	-0.6644
C	-1.3221	0.3126	-0.0485	C	-1.3319	0.2933	-0.0776
C	2.3783	1.1469	-0.2344	C	2.3930	1.1114	-0.2461
C	2.7527	-1.5530	0.2095	C	2.7677	-1.6051	0.1214
C	-2.7702	-1.6041	-0.5359	C	-2.8220	-1.5566	-0.6829
C	-2.4994	1.0272	0.2666	C	-2.4721	0.9983	0.3782
C	-0.0825	2.4137	0.1151	C	-0.0830	2.4040	0.0342
C	-3.9377	-0.8970	-0.1205	C	-3.9460	-0.8717	-0.1395
C	-3.7616	0.4513	0.2609	C	-3.7418	0.4350	0.3677
O	-2.4596	2.3495	0.5889	O	-2.3841	2.2762	0.8183
C	-1.3143	3.1374	0.4190	C	-1.2734	3.1092	0.4303
C	1.1224	3.2149	-0.0830	C	1.1046	3.1944	-0.2317
C	3.6608	0.6202	-0.3168	C	3.6733	0.5912	-0.2936
C	3.8887	-0.7615	-0.1377	C	3.9067	-0.7978	-0.1360

O	-1.4617	4.3236	0.5968	O	-1.4829	4.2967	0.5462
O	1.2292	4.4187	-0.0888	O	1.2149	4.3988	-0.3169
H	4.4636	1.3231	-0.4845	H	4.4774	1.2979	-0.4426
N	-5.1749	-1.4890	-0.1208	N	-5.1967	-1.4509	-0.1167
H	2.8655	-2.5982	0.4614	H	2.8764	-2.6684	0.2923
C	6.2788	-0.4046	-0.5341	C	6.3081	-0.4250	-0.4888
C	5.6086	-2.7570	1.7331	C	5.5908	-2.5688	1.9121
H	-2.8441	-2.6225	-0.8929	H	-2.9230	-2.5522	-1.0926
H	4.7199	-2.4093	2.2677	H	4.6639	-2.2021	2.3622
H	6.4578	-2.1484	2.0596	H	6.4030	-1.9019	2.2190
H	5.8022	-3.7933	2.0288	H	5.7997	-3.5641	2.3184
H	8.3047	-0.3127	-1.2082	H	8.3218	-0.3185	-1.2120
H	7.4439	-1.7298	-1.8190	H	7.4565	-1.7528	-1.7816
H	8.0225	-1.6834	-0.1385	H	8.0789	-1.6773	-0.1188

compound 5

S0				S1			
	x	y	z		x	y	z
H	-7.3900	-0.7236	-0.6343	H	-7.4144	-0.6323	-0.7865
H	-6.3976	0.7106	-0.5377	H	-6.3849	0.7732	-0.6470
C	-6.7785	-0.2314	1.3799	C	-6.8591	-0.1645	1.2494
H	-6.6310	-2.5513	-1.2832	H	-6.6756	-2.4837	-1.4249
C	-5.3933	-3.5354	0.1897	C	-5.5403	-3.4956	0.1103
C	-5.6007	-2.4983	-0.9214	C	-5.6629	-2.4538	-1.0117
H	-0.8486	-1.4966	-1.0294	H	-0.8668	-1.5972	-0.9065
N	-5.3588	-1.1095	-0.5186	N	-5.4016	-1.0745	-0.6076
H	2.3316	-3.7689	-0.4273	H	2.4125	-3.7947	-0.1349
H	1.7873	-3.5099	1.2218	H	1.9152	-3.4237	1.5072
H	4.0301	-4.7405	1.0223	H	4.1861	-4.6089	1.3430
H	4.1164	-3.2773	2.0095	H	4.2659	-3.0629	2.1935
H	4.9160	-3.5251	-0.9391	H	4.9801	-3.5728	-0.7457
H	6.0124	-3.3301	0.4313	H	6.1131	-3.2035	0.5576
H	6.1834	-0.8968	-1.5428	H	6.1365	-0.9738	-1.6635
H	6.9871	-1.4509	-0.0703	H	7.0112	-1.4000	-0.1880
H	6.1416	0.4929	1.1875	H	6.1755	0.6442	0.9410
H	7.0113	1.0478	-0.2452	H	6.9864	1.0785	-0.5656
H	4.9567	1.5276	-1.4391	H	4.8761	1.4020	-1.7299
C	6.1110	0.5251	0.0923	C	6.1061	0.5795	-0.1508
C	5.0121	-3.0785	0.0648	C	5.0922	-3.0288	0.2069
C	3.9445	-3.6498	0.9931	C	4.0687	-3.5283	1.2212
H	0.4033	-1.6609	0.4866	H	0.4565	-1.6246	0.7166
C	-0.0939	1.0456	-0.0641	C	-0.1252	0.9973	-0.0604
O	-2.4349	2.5318	0.3847	O	-2.4107	2.4805	0.5107
C	-1.3975	0.4303	-0.2131	C	-1.3940	0.3871	-0.1980
C	-1.6654	-0.8772	-0.6851	C	-1.6812	-0.9421	-0.6232
C	1.2819	-1.0805	0.2368	C	1.3263	-1.0697	0.3856
C	1.1479	0.3035	-0.0218	C	1.1666	0.2652	-0.0287

C	-2.5408	1.2165	0.0492	C	-2.5432	1.1873	0.0747		
C	-2.9429	-1.3858	-0.7850	C	-2.9750	-1.4203	-0.7494		
C	2.4983	-1.7323	0.2431	C	2.5557	-1.7169	0.3820		
C	2.3577	1.0170	-0.1662	C	2.3390	0.9903	-0.3276		
C	-0.0369	2.4447	0.0445	C	-0.0202	2.4068	0.1547		
C	3.6959	-0.9897	-0.0026	C	3.7180	-0.9764	-0.0003		
C	3.6143	0.4132	-0.1858	C	3.6090	0.4073	-0.3233		
O	2.3572	2.3749	-0.2978	O	2.2888	2.3218	-0.5958		
C	1.2281	3.1647	-0.0541	C	1.2025	3.1235	-0.0931		
C	-1.2368	3.2470	0.2639	C	-1.1930	3.1824	0.5135		
C	-3.8357	0.7205	-0.0138	C	-3.8374	0.7196	-0.0379		
C	-4.0824	-0.6075	-0.4238	C	-4.1027	-0.6086	-0.4617		
C	4.8551	1.2602	-0.3781	C	4.8220	1.2452	-0.6428		
O	1.4205	4.3573	-0.0083	O	1.4538	4.3062	-0.0088		
O	-1.3235	4.4440	0.4078	O	-1.2660	4.3524	0.8239		
H	-4.6330	1.4054	0.2379	H	-4.6293	1.4107	0.2159		
C	2.5609	-3.2252	0.5004	C	2.6595	-3.1787	0.7427		
N	4.9210	-1.6221	-0.0043	N	4.9518	-1.5939	-0.0418		
C	6.1156	-0.9035	-0.4423	C	6.1217	-0.8887	-0.5645		
H	4.7395	2.2073	0.1556	H	4.6976	2.2401	-0.2080		
H	-3.0632	-2.3913	-1.1644	H	-3.1066	-2.4412	-1.0842		
C	-6.5176	-0.2987	-0.1303	C	-6.5455	-0.2362	-0.2523		
H	-4.9691	-2.7375	-1.7834	H	-4.9937	-2.7168	-1.8388		
H	-5.5687	-4.5430	-0.2016	H	-5.7135	-4.5011	-0.2889		
H	-6.0854	-3.3688	1.0198	H	-6.2746	-3.3135	0.9002		
H	-4.3750	-3.4967	0.5864	H	-4.5455	-3.4748	0.5637		
H	-6.9981	-1.2221	1.7876	H	-7.1235	-1.1492	1.6456		
H	-7.6378	0.4166	1.5812	H	-7.7042	0.5101	1.4241		
H	-5.9163	0.1727	1.9172	H	-6.0030	0.2102	1.8169		

compound 7

	S0			S1-bright			S1-dark		
	x	y	z	x	y	z	x	y	z
C	-5.8303	0.3356	1.2766	-5.9652	0.1700	1.2557	-5.3103	0.2420	1.7292
H	-5.2457	1.2669	-0.5927	-5.2079	1.2794	-0.4518	-5.3633	1.3399	-0.1497
H	-6.3985	-0.0311	-0.7741	-6.3559	0.0233	-0.8667	-6.4006	-0.0728	-0.1083
C	-4.5847	-2.9765	0.3876	-4.4510	-3.0019	0.3380	-5.0187	-2.8766	-0.0846
H	-4.2909	-2.4161	-1.6862	-4.3566	-2.3682	-1.7376	-4.2113	-2.0152	-1.9106
O	2.1230	-3.9767	0.6441	4.3377	-4.0972	0.4015	1.8258	-4.0377	0.5773
N	3.2192	-3.4881	0.4116	3.2767	-3.4880	0.3503	3.0066	-3.5810	0.4144
H	-0.0275	-1.5255	-1.0847	-0.0174	-1.3978	-1.2755	-0.0272	-1.4301	-1.1573
H	1.1951	-1.8308	0.4277	1.2039	-1.8881	0.3143	1.0828	-1.8695	0.4193
C	0.9731	0.9254	-0.1107	1.0048	0.9115	-0.0741	1.0334	0.9188	-0.1004
H	-2.3234	-2.1692	-1.2143	-2.3374	-2.0784	-1.4101	-2.3533	-2.0395	-1.2874
O	3.5512	1.9760	-0.3776	3.5244	2.0135	-0.3280	3.6252	1.8849	-0.3461
C	2.1416	0.0521	-0.1037	2.1317	0.0623	-0.0416	2.1406	-0.0100	-0.0626
C	2.1124	-1.3217	0.1597	2.1226	-1.3432	0.1387	2.0205	-1.3885	0.1769

C	-0.7710	-0.8220	-0.7259	-0.7760	-0.7487	-0.8506	-0.7701	-0.7271	-0.8035
C	-0.3692	0.4484	-0.2522	-0.3864	0.4526	-0.2323	-0.3539	0.5091	-0.2644
C	3.4047	0.6470	-0.2726	3.4088	0.6776	-0.1573	3.4218	0.5342	-0.2011
C	3.2840	-2.0538	0.1362	3.3194	-2.0411	0.1647	3.1501	-2.2023	0.1955
C	-2.0883	-1.1863	-0.8211	-2.0963	-1.1316	-0.9406	-2.0933	-1.0622	-0.9004
C	-1.4176	1.3508	0.0192	-1.4157	1.3238	0.1673	-1.3668	1.4460	0.0482
C	1.1941	2.2900	0.0425	1.1574	2.3163	-0.0326	1.2775	2.2741	-0.0240
C	-3.1333	-0.2919	-0.4518	-3.1251	-0.2819	-0.4515	-3.1061	-0.1579	-0.4495
C	-2.7517	0.9989	-0.0409	-2.7515	0.9720	0.0808	-2.7025	1.1218	0.0052
O	-1.1656	2.6313	0.3752	-1.1538	2.5630	0.6312	-1.0681	2.7087	0.3982
C	0.0909	3.2115	0.3058	0.0719	3.1993	0.3340	0.2075	3.2467	0.2328
C	2.5212	2.8781	-0.0818	2.4633	2.9179	-0.2321	2.6518	2.8105	-0.1773
C	4.5793	-0.1079	-0.3225	4.5909	-0.0446	-0.1338	4.5570	-0.2743	-0.1893
C	4.5234	-1.4704	-0.1270	4.5593	-1.4266	0.0230	4.4242	-1.6334	-0.0018
N	-4.4424	-0.6606	-0.5431	-4.4380	-0.6598	-0.5199	-4.4129	-0.5021	-0.4757
H	-3.4720	1.7622	0.2314	-3.4758	1.6940	0.4409	-3.4135	1.8930	0.2777
O	0.1495	4.3922	0.5165	0.0823	4.3930	0.4850	0.3082	4.4274	0.3888
O	2.8381	4.0324	-0.0255	2.7512	4.0808	-0.3361	2.9441	3.9762	-0.2203
H	5.5188	0.4104	-0.4857	5.5266	0.4949	-0.2425	5.5300	0.1922	-0.3126
H	5.4156	-2.0868	-0.1436	5.4647	-2.0216	0.0382	5.2844	-2.2937	0.0260
C	-5.5113	0.2754	-0.2082	-5.5219	0.2637	-0.1972	-5.4283	0.3080	0.2145
C	-4.8181	-2.0503	-0.7952	-4.8133	-2.0480	-0.7921	-4.8835	-1.7483	-1.0930
O	4.2664	-4.1164	0.3885	2.1772	-4.0217	0.4456	4.0527	-4.3051	0.4317
H	-5.8802	-2.0463	-1.0631	-5.8947	-2.0530	-0.9578	-5.8502	-1.5193	-1.5536
H	-5.1706	-2.6597	1.2568	-4.9377	-2.7075	1.2730	-5.7726	-2.6474	0.6752
H	-3.5305	-2.9922	0.6842	-3.3713	-3.0312	0.5131	-4.0667	-3.0716	0.4201
H	-4.8790	-4.0002	0.1342	-4.7815	-4.0131	0.0834	-5.3295	-3.7899	-0.5986
H	-6.5962	1.0923	1.4753	-6.7406	0.9153	1.4554	-6.1147	0.8307	2.1781
H	-4.9407	0.5889	1.8638	-5.1260	0.3529	1.9354	-4.3574	0.6433	2.0862
H	-6.2089	-0.6272	1.6372	-6.3825	-0.8168	1.4815	-5.4002	-0.7879	2.0873

compound 8

S0				S1			
	x	y	z		x	y	z
H	-6.2818	-2.1763	0.0599	H	-6.2570	-2.1975	0.1223
H	-6.1747	-0.8226	1.1964	H	-6.1483	-0.8358	1.2502
H	-5.8631	1.4929	1.8232	H	-5.8794	1.4876	1.8524
H	-5.5343	3.2102	1.5449	H	-5.5589	3.2074	1.5766
C	-5.6269	-1.3823	0.4333	C	-5.6093	-1.3876	0.4747
H	-6.0844	-0.0025	-1.1666	H	-6.1344	-0.0221	-1.1166
H	-4.7427	-1.0644	-1.5234	H	-4.7861	-1.0643	-1.5046
C	-5.1325	2.1993	1.4199	C	-5.1577	2.1974	1.4385
H	-5.7913	1.9976	-0.6284	H	-5.8542	1.9936	-0.5968
H	2.3797	4.3583	-1.3413	H	2.4183	4.3283	-1.3104
N	3.1555	4.1763	-0.7180	N	3.2977	4.0129	-0.9284
H	-0.0465	1.9870	0.6078	H	-0.0848	2.0253	0.6430
H	1.3098	2.2788	-0.8247	H	1.3951	2.2370	-0.9117

C	1.3147	-0.4008	-0.0182	C	1.2740	-0.3745	0.0355
H	-2.4031	2.3772	0.4767	H	-2.4456	2.4122	0.4523
O	3.9332	-1.1503	0.6660	O	3.8332	-1.1003	0.9343
C	2.3852	0.5965	0.0038	C	2.3765	0.5974	0.0602
C	2.2496	1.9395	-0.4098	C	2.3139	1.8802	-0.4613
C	-0.6735	1.1836	0.2460	C	-0.7032	1.2162	0.2721
C	-0.0893	-0.0672	-0.0736	C	-0.1048	-0.0370	-0.0271
C	3.6653	0.1579	0.3839	C	3.6478	0.1570	0.5120
C	3.3187	2.8366	-0.3588	C	3.4435	2.7574	-0.4434
C	-2.0317	1.4045	0.1851	C	-2.0687	1.4344	0.1822
C	-1.0076	-1.1020	-0.3621	C	-1.0218	-1.0689	-0.3722
C	1.6823	-1.7489	0.0101	C	1.6662	-1.7564	-0.0091
C	-2.9355	0.3753	-0.2193	C	-2.9599	0.4050	-0.2171
C	-2.3762	-0.8998	-0.4577	C	-2.3863	-0.8662	-0.4695
O	-0.5880	-2.3815	-0.5633	O	-0.5864	-2.3376	-0.6594
C	0.7138	-2.8075	-0.2860	C	0.7256	-2.7813	-0.4040
C	3.0412	-2.1743	0.3604	C	2.9599	-2.1832	0.4234
C	4.7387	1.0474	0.4675	C	4.7739	1.0141	0.5264
C	4.5650	2.3754	0.1170	C	4.6832	2.3133	0.0771
N	-4.2808	0.6094	-0.3532	N	-4.3213	0.6253	-0.3602
H	-2.9829	-1.7630	-0.6909	H	-2.9868	-1.7224	-0.7442
O	0.9117	-3.9951	-0.3742	O	0.9140	-3.9697	-0.5633
O	3.4568	-3.3028	0.4806	O	3.4767	-3.2690	0.4946
H	5.7043	0.6703	0.7855	H	5.7068	0.6131	0.9067
H	5.4041	3.0626	0.1763	H	5.5374	2.9811	0.1059
C	-5.2008	-0.4714	-0.7250	C	-5.2290	-0.4710	-0.6970
C	-4.8571	1.9269	-0.0643	C	-4.9070	1.9279	-0.0525
H	3.9981	4.6333	-1.0413	H	4.0672	4.6661	-0.9299
H	-4.1993	2.6994	-0.4751	H	-4.2661	2.7128	-0.4702
H	-4.2209	2.1191	2.0185	H	-4.2346	2.1145	2.0188
H	-4.7643	-1.8524	0.9131	H	-4.7241	-1.8366	0.9330

compound 10

S0				S1			
	x	y	z		x	y	z
H	5.7486	0.7061	-0.3637	H	5.7529	0.7040	-0.4053
H	2.3924	-2.8014	0.7181	H	2.4781	-2.8221	0.7843
H	4.7511	-3.3813	0.6650	H	4.8404	-3.3912	0.7067
H	7.7980	0.0719	0.5627	H	7.8275	0.1571	0.4450
H	8.2926	-3.1499	0.3279	H	8.3868	-3.0751	0.2998
H	6.7621	-3.8351	-0.2217	H	6.8683	-3.7670	-0.3012
H	6.9588	-3.3386	1.4740	H	7.0262	-3.3186	1.4113
C	7.7894	-0.7206	-0.1982	C	7.8269	-0.6665	-0.2776
C	5.4555	-1.3802	0.1238	C	5.5064	-1.3947	0.1338
C	4.4550	-2.3639	0.4439	C	4.5368	-2.3791	0.4775
C	3.1266	-2.0376	0.4755	C	3.1972	-2.0514	0.5206
H	-7.2091	-2.0278	-0.6180	H	-7.2635	-1.9723	-0.6785

C	-6.7292	-1.4397	1.4063	C	-6.8060	-1.3301	1.3340
H	-6.5648	-0.4088	-0.4968	H	-6.5901	-0.3621	-0.5937
H	-6.0589	-3.6450	-1.2457	H	-6.1197	-3.6131	-1.2954
C	-4.6037	-4.3160	0.2040	C	-4.7622	-4.2967	0.2404
H	-0.6725	-1.2853	-1.0570	H	-0.6804	-1.3841	-0.8887
H	0.6001	-1.1369	0.4776	H	0.6403	-1.1580	0.5811
C	-0.5188	1.3588	-0.0888	C	-0.5206	1.3209	-0.0752
H	-2.6251	-2.6633	-1.1781	H	-2.6553	-2.7275	-1.0248
O	1.5495	3.2326	-0.4289	O	1.5545	3.1711	-0.4790
C	0.8716	0.9230	-0.0841	C	0.8784	0.8899	-0.0396
C	1.3131	-0.3720	0.1981	C	1.3470	-0.3972	0.2758
C	-1.6055	-0.8743	-0.6959	C	-1.6179	-0.9288	-0.5970
C	-1.6412	0.4570	-0.2138	C	-1.6370	0.4303	-0.1895
C	1.8809	1.9147	-0.2824	C	1.8655	1.8806	-0.2877
C	2.6750	-0.7175	0.1906	C	2.7155	-0.7462	0.2357
C	-2.7328	-1.6610	-0.7868	C	-2.7638	-1.7000	-0.7028
C	-2.9306	0.9562	0.0766	C	-2.9344	0.9513	0.0583
C	-0.7760	2.7282	0.0400	C	-0.7727	2.7288	0.0502
C	-4.0168	-1.1678	-0.4048	C	-4.0463	-1.1681	-0.4118
C	-4.0771	0.1770	0.0220	C	-4.0900	0.1927	-0.0278
O	-3.1201	2.2545	0.4393	O	-3.1175	2.2580	0.4254
C	-2.1201	3.2275	0.3281	C	-2.1020	3.2260	0.3472
C	0.2824	3.7275	-0.1298	C	0.2511	3.7111	-0.1430
C	3.2207	1.6085	-0.3260	C	3.2287	1.5693	-0.3450
C	3.6646	0.2795	-0.1103	C	3.6741	0.2529	-0.0991
N	-5.1433	-1.9481	-0.4950	N	-5.2007	-1.9270	-0.5273
H	-5.0064	0.6553	0.2967	H	-5.0186	0.6954	0.2054
O	-2.4670	4.3655	0.5370	O	-2.4559	4.3666	0.5626
O	0.1724	4.9306	-0.1099	O	0.2225	4.9210	-0.1153
H	3.9331	2.4092	-0.4946	H	3.9298	2.3671	-0.5651
C	5.0310	-0.0727	-0.1387	C	5.0491	-0.0780	-0.1506
C	-6.4566	-1.4246	-0.1030	C	-6.5047	-1.3692	-0.1709
C	-5.0630	-3.3556	-0.9002	C	-5.1373	-3.3386	-0.8996
H	-4.4074	-3.4419	-1.7731	H	-4.4353	-3.4582	-1.7326
N	6.7843	-1.7342	0.0828	N	6.8426	-1.6863	0.0788
C	7.2105	-3.0813	0.4366	C	7.3031	-3.0348	0.3893
H	7.6184	-0.2503	-1.1744	H	7.6322	-0.2583	-1.2751
H	8.7747	-1.1854	-0.2167	H	8.8172	-1.1171	-0.2817
H	-4.5510	-5.3373	-0.1876	H	-4.7032	-5.3243	-0.1346
H	-5.3004	-4.3111	1.0467	H	-5.5081	-4.2701	1.0400
H	-3.6141	-4.0455	0.5825	H	-3.7932	-4.0359	0.6750
H	-6.7216	-2.4604	1.7989	H	-6.8268	-2.3377	1.7593
H	-7.7128	-1.0047	1.6115	H	-7.7828	-0.8668	1.5096
H	-5.9812	-0.8602	1.9540	H	-6.0535	-0.7500	1.8751

compound 11

S0				S1			

	x	y	z		x	y	z
H	-0.8209	-1.0594	-0.6960	H	-0.8558	-1.0737	-0.7774
C	-7.5011	-2.7526	-0.5870	C	-7.5766	-2.7165	-0.4933
H	-8.9655	-0.8655	0.2843	H	-8.9977	-0.7888	0.3306
H	-7.7454	-0.0727	1.2904	H	-7.7642	-0.0613	1.3695
H	-7.9576	0.4212	-0.4021	H	-7.9618	0.4950	-0.3100
H	-5.8597	0.8839	0.5096	H	-5.8556	0.8866	0.5403
H	-5.0651	-3.1208	-0.9357	H	-5.1432	-3.1328	-0.9356
C	-4.7205	-2.1408	-0.6314	C	-4.7918	-2.1556	-0.6347
C	-5.6686	-1.1553	-0.1836	C	-5.7101	-1.1663	-0.1801
C	-5.1825	0.1041	0.1849	C	-5.1919	0.1041	0.1952
H	1.9314	-3.8650	-0.0642	H	1.9788	-3.8631	-0.2216
H	1.5799	-3.3850	1.5880	H	1.5907	-3.4618	1.4424
H	3.5548	-5.0045	1.3518	H	3.5735	-5.0743	1.1515
H	3.9747	-3.5040	2.1854	H	3.9543	-3.6342	2.1030
H	4.4640	-4.1344	-0.7742	H	4.5429	-4.0270	-0.8786
H	5.6908	-4.0211	0.4907	H	5.7319	-4.0448	0.4292
H	6.1059	-1.8323	-1.7180	H	6.1480	-1.7493	-1.6403
H	6.9237	-2.3977	-0.2577	H	6.9933	-2.3392	-0.2032
H	6.5398	-0.2365	0.8615	H	6.5637	-0.2113	0.9761
H	7.3630	0.0331	-0.6768	H	7.3960	0.1047	-0.5490
H	5.3219	0.7693	-1.7616	H	5.3673	0.8554	-1.6310
C	6.4193	-0.2900	-0.2266	C	6.4525	-0.2407	-0.1141
C	4.7198	-3.6290	0.1720	C	4.7758	-3.6102	0.1182
C	3.6548	-3.9222	1.2241	C	3.6720	-3.9848	1.1035
H	0.4782	-1.3779	0.7968	H	0.4821	-1.4486	0.6501
C	0.4180	1.3259	0.0578	C	0.4230	1.2969	0.0364
O	-1.5612	3.2349	0.6521	O	-1.5618	3.1831	0.6690
C	-0.9895	0.9445	0.0679	C	-0.9920	0.9226	0.0176
C	-1.4932	-0.2994	-0.3193	C	-1.5221	-0.3159	-0.3862
C	1.4188	-0.9877	0.4300	C	1.4319	-1.0232	0.3496
C	1.5048	0.3747	0.0578	C	1.5058	0.3567	0.0368
C	-1.9504	1.9497	0.3963	C	-1.9310	1.9251	0.3809
C	-2.8672	-0.5935	-0.2922	C	-2.9008	-0.6160	-0.3255
C	2.4994	-1.8445	0.4033	C	2.5345	-1.8644	0.3521
C	2.8014	0.8455	-0.2466	C	2.8126	0.8441	-0.2248
C	0.7220	2.6924	0.0400	C	0.7252	2.7006	0.0174
C	3.7809	-1.3501	0.0005	C	3.8194	-1.3418	0.0429
C	3.9270	0.0263	-0.3070	C	3.9511	0.0437	-0.2356
O	3.0189	2.1662	-0.5103	O	3.0231	2.1695	-0.5101
C	2.0718	3.1642	-0.2590	C	2.0602	3.1693	-0.2925
C	-0.2876	3.7090	0.3447	C	-0.2514	3.6996	0.3325
C	-3.2994	1.6909	0.4615	C	-3.3018	1.6614	0.4595
C	-3.8044	0.4062	0.1385	C	-3.8080	0.3870	0.1229
C	5.2722	0.6168	-0.6746	C	5.2977	0.6627	-0.5511
O	2.4615	4.3022	-0.3749	O	2.4585	4.3090	-0.4196

O	-0.1308	4.9043	0.4298	O	-0.1737	4.9057	0.4066
H	-3.9724	2.4981	0.7307	H	-3.9634	2.4639	0.7678
C	2.3272	-3.2997	0.7918	C	2.3563	-3.3375	0.6678
N	4.8718	-2.1904	-0.0350	N	4.9450	-2.1642	0.0596
C	6.1317	-1.7353	-0.6202	C	6.1842	-1.6815	-0.5381
H	5.3706	1.6113	-0.2315	H	5.3722	1.6438	-0.0739
C	-3.3815	-1.8626	-0.6821	C	-3.4427	-1.8755	-0.6979
N	-7.0091	-1.4613	-0.1274	N	-7.0547	-1.4115	-0.1042
C	-7.9622	-0.4404	0.2797	C	-7.9887	-0.3818	0.3464
H	-2.6876	-2.6269	-1.0224	H	-2.7634	-2.6480	-1.0481
H	-7.2967	-2.9211	-1.6528	H	-7.3572	-2.9305	-1.5460
H	-8.5805	-2.7916	-0.4429	H	-8.6557	-2.7283	-0.3544
H	-7.0602	-3.5806	-0.0190	H	-7.1374	-3.5125	0.1187

compound 12

S0				S1			
	x	y	z		x	y	z
H	2.9461	-4.1353	0.2418	H	3.0541	-4.1499	0.0204
H	1.6627	-3.5919	1.3264	H	1.6640	-3.6975	1.0169
H	-5.4648	0.8697	0.4616	H	-5.4573	0.8692	0.5286
H	-2.3345	-2.7566	-0.8759	H	-2.4243	-2.7591	-0.9572
H	-4.7193	-3.2118	-0.7810	H	-4.8103	-3.2046	-0.8212
H	-6.7291	-3.5960	0.1574	H	-6.8059	-3.5237	0.2451
H	-8.5935	-0.8452	0.3036	H	-8.6222	-0.7703	0.4061
H	-7.5624	0.3955	-0.4305	H	-7.5847	0.4757	-0.3034
H	-7.3674	-0.0280	1.2829	H	-7.3651	-0.0139	1.3939
C	-7.1509	-2.7946	-0.4613	C	-7.2366	-2.7375	-0.3855
C	-5.2989	-1.2032	-0.1309	C	-5.3455	-1.2084	-0.1244
C	-4.3624	-2.2230	-0.5232	C	-4.4438	-2.2237	-0.5518
C	-3.0194	-1.9667	-0.5783	C	-3.0901	-1.9657	-0.6286
H	6.9281	-1.2468	0.4880	H	6.8904	-1.2087	0.7218
C	7.4906	-3.2132	-0.2229	C	7.6074	-3.0962	-0.0638
H	6.6022	-1.5195	-1.2247	H	6.7331	-1.3771	-1.0263
H	0.8451	-1.4703	0.8921	H	0.8462	-1.5613	0.7088
H	-0.4479	-1.2015	-0.6146	H	-0.4903	-1.2075	-0.7387
C	0.8198	1.2006	0.0297	C	0.8272	1.1650	0.0059
C	2.6944	-3.4041	1.0211	C	2.7140	-3.4728	0.8144
O	-1.1337	3.1640	0.5168	O	-1.1284	3.1022	0.5652
C	-0.5923	0.8404	0.0484	C	-0.5946	0.8134	-0.0056
C	-1.1112	-0.4144	-0.2798	C	-1.1440	-0.4275	-0.3703
C	1.7923	-1.1134	0.5097	C	1.8036	-1.1638	0.3952
C	1.8940	0.2344	0.0761	C	1.8954	0.2093	0.0387
C	-1.5404	1.8738	0.3214	C	-1.5164	1.8418	0.3246
C	-2.4892	-0.6872	-0.2477	C	-2.5279	-0.7049	-0.2974
C	2.8606	-1.9866	0.5333	C	2.8903	-2.0249	0.4264
C	3.1934	0.6787	-0.2459	C	3.2061	0.6708	-0.2377
C	1.1471	2.5592	-0.0463	C	1.1545	2.5613	-0.0595

C	4.1444	-1.5207	0.0976	C	4.1800	-1.5275	0.0854
C	4.2907	-0.1725	-0.2671	C	4.3140	-0.1643	-0.2277
O	3.4449	1.9800	-0.5518	O	3.4542	1.9827	-0.5423
C	2.5075	3.0002	-0.3515	C	2.5017	3.0001	-0.3710
C	0.1493	3.6042	0.1999	C	0.1925	3.5872	0.2097
C	-2.8932	1.6375	0.3906	C	-2.8929	1.6015	0.4155
C	-3.4145	0.3457	0.1273	C	-3.4179	0.3252	0.1217
N	5.2012	-2.3853	0.0608	N	5.2619	-2.3938	0.0438
H	5.2521	0.2430	-0.5390	H	5.2768	0.2740	-0.4577
O	2.9134	4.1272	-0.5083	O	2.9148	4.1303	-0.5285
O	0.3234	4.7996	0.2292	O	0.2852	4.7939	0.2333
H	-3.5563	2.4661	0.6159	H	-3.5412	2.4238	0.6987
C	-4.7969	0.0658	0.1795	C	-4.8068	0.0657	0.2077
C	6.5730	-1.9950	-0.2365	C	6.6342	-1.9212	-0.0796
H	5.0785	-3.2830	0.5034	H	5.1625	-3.2226	0.6135
N	-6.6438	-1.4866	-0.0703	N	-6.6930	-1.4308	-0.0337
C	-7.5839	-0.4356	0.2875	C	-7.6092	-0.3734	0.3886
H	-6.9378	-3.0256	-1.5134	H	-7.0262	-2.9826	-1.4333
H	-8.2323	-2.8090	-0.3282	H	-8.3150	-2.7292	-0.2407
H	3.3346	-3.6185	1.8875	H	3.2798	-3.7319	1.7205
H	7.4966	-3.6930	0.7620	H	7.5500	-3.6455	0.8830
H	8.5161	-2.9146	-0.4540	H	8.6348	-2.7405	-0.1787
H	7.1742	-3.9519	-0.9657	H	7.3907	-3.7938	-0.8785

compound 13

S0				S1			
	x	y	z		x	y	z
H	-5.6582	-3.8249	-1.3626	H	5.5851	-3.6639	1.5264
H	-1.9024	-3.7635	-0.6697	H	1.9663	-3.8230	0.3587
H	-3.0848	-3.4607	-1.9321	H	2.9359	-3.5340	1.7919
H	-3.8981	-5.3414	-0.5796	H	4.0266	-5.3333	0.5715
H	-6.0679	-4.3651	0.2703	H	6.2375	-4.2920	0.0050
C	-2.9247	-3.4128	-0.8455	C	2.9408	-3.4511	0.6953
C	-3.9371	-4.3349	-0.1522	C	4.0684	-4.3298	0.1359
H	5.2005	1.0325	-0.5507	H	-5.1998	1.0115	0.5858
H	2.1743	-2.6106	0.9678	H	-2.2396	-2.6241	-1.0243
H	4.5657	-3.0198	0.8401	H	-4.6300	-3.0380	-0.8682
H	6.5610	-3.3946	-0.1103	H	-6.6116	-3.3523	0.2199
H	8.3687	-0.6085	-0.3885	H	-8.3917	-0.5739	0.4501
H	7.3252	0.6281	0.3358	H	-7.3400	0.6688	-0.2441
H	7.1095	0.1611	-1.3638	H	-7.1159	0.1446	1.4423
C	6.9823	-2.5613	0.4648	C	-7.0431	-2.5477	-0.3863
C	5.0901	-1.0227	0.1122	C	-5.1269	-1.0510	-0.1185
C	4.1830	-2.0478	0.5565	C	-4.2455	-2.0694	-0.5802
C	2.8363	-1.8173	0.6303	C	-2.8896	-1.8293	-0.6685
H	-1.0693	-1.4471	-0.7724	H	1.0715	-1.5171	0.6121
H	0.2503	-1.1024	0.6965	H	-0.2812	-1.1061	-0.7953

C	-1.0796	1.2506	0.0031	C	1.0817	1.2291	-0.0171
O	0.8216	3.2341	-0.5979	O	-0.8379	3.1790	0.6231
C	0.3389	0.9178	-0.0377	C	-0.3457	0.8981	-0.0142
C	0.8904	-0.3145	0.3209	C	-0.9183	-0.3256	-0.4001
C	-2.0161	-1.0933	-0.3844	C	2.0292	-1.1224	0.2941
C	-2.1348	0.2636	0.0110	C	2.1350	0.2575	-0.0248
C	1.2593	1.9602	-0.3657	C	-1.2477	1.9310	0.3548
C	2.2728	-0.5607	0.2686	C	-2.3048	-0.5845	-0.3155
C	-3.0723	-1.9819	-0.3685	C	3.1088	-1.9965	0.2948
C	-3.4381	0.6953	0.3502	C	3.4515	0.7115	-0.3102
C	-1.4313	2.6050	0.0452	C	1.4267	2.6226	-0.0526
C	-4.3489	-1.5294	0.0848	C	4.3917	-1.5085	-0.0572
C	-4.5139	-0.1756	0.4214	C	4.5416	-0.1398	-0.3443
O	-3.7038	1.9998	0.6304	O	3.7097	2.0267	-0.5886
C	-2.7896	3.0299	0.3797	C	2.7741	3.0518	-0.3743
C	-0.4633	3.6595	-0.2680	C	0.4841	3.6542	0.2607
C	2.6151	1.7490	-0.4549	C	-2.6262	1.7088	0.4575
C	3.1686	0.4777	-0.1591	C	-3.1741	0.4481	0.1392
H	-5.4831	0.2142	0.7141	H	5.5141	0.2731	-0.5940
O	-3.2092	4.1535	0.5238	O	3.2005	4.1801	-0.5081
O	-0.6653	4.8485	-0.3430	O	0.5956	4.8582	0.3162
H	3.2562	2.5825	-0.7219	H	-3.2585	2.5333	0.7689
C	4.5554	0.2247	-0.2293	C	-4.5655	0.2069	0.2366
N	6.4394	-1.2805	0.0336	N	-6.4764	-1.2559	-0.0164
C	7.3514	-0.2188	-0.3635	C	-7.3729	-0.1925	0.4324
H	6.8004	-2.7537	1.5306	H	-6.8548	-2.7733	-1.4427
H	8.0600	-2.5607	0.3036	H	-8.1186	-2.5289	-0.2220
N	-5.3981	-2.3996	0.1818	N	5.4788	-2.3639	-0.1572
C	-5.3507	-3.7730	-0.3065	C	5.4273	-3.6943	0.4346
H	-6.3055	-2.0076	0.3848	H	6.3835	-1.9138	-0.1498
H	-3.6970	-4.4189	0.9139	H	3.9589	-4.4356	-0.9498

compound 15

S0				S1			
	x	y	z		x	y	z
H	-2.1061	-3.4434	-0.1562	H	-2.0800	-3.3873	-0.6645
H	-1.5260	-3.0471	-1.7646	H	-1.5927	-2.7578	-2.2282
H	-3.9535	-3.8689	-1.6935	H	-4.0175	-3.5975	-2.1481
H	-3.7771	-2.2779	-2.4415	H	-3.8799	-1.9132	-2.6608
H	-4.6096	-2.8447	0.4535	H	-4.5547	-2.9154	0.1588
H	-5.6479	-2.2341	-0.8372	H	-5.6607	-2.1143	-0.9610
H	-5.3791	-0.1676	1.5070	H	-5.2714	-0.4034	1.6367
H	-6.2672	-0.3278	-0.0116	H	-6.2297	-0.3791	0.1522
H	-5.0964	1.6181	-0.9669	H	-5.0975	1.6959	-0.5924
H	-5.8489	2.0747	0.5634	H	-5.7953	1.9362	1.0109
H	-3.7350	1.9772	1.7524	H	-3.6365	1.6805	2.1019
C	-5.0568	1.4654	0.1177	C	-5.0172	1.4011	0.4598

C	-4.6193	-2.2321	-0.4631	C	-4.6130	-2.1749	-0.6550
C	-3.6730	-2.8291	-1.4998	C	-3.7231	-2.5970	-1.8190
C	3.8621	-3.3612	0.4257	C	3.8702	-3.3706	0.5099
C	1.8458	-3.2200	1.7314	C	1.7263	-3.3096	1.6058
C	2.9596	-3.9579	1.2772	C	2.8835	-4.0156	1.2281
H	0.1747	-1.5971	-0.8058	H	0.1754	-1.4276	-1.1506
C	1.6224	-1.9315	1.2903	C	1.5600	-1.9873	1.2208
C	1.1305	0.8880	0.0075	C	1.1945	0.8612	0.0398
O	3.4958	2.0138	-0.9052	O	3.5198	2.0457	-0.8519
C	2.3322	0.0591	-0.1030	C	2.3635	0.0778	0.0039
C	2.5014	-1.3022	0.3686	C	2.5188	-1.3145	0.4322
C	-0.5940	-0.9131	-0.4689	C	-0.5930	-0.8167	-0.6905
C	-0.2173	0.3733	-0.0158	C	-0.2122	0.3545	-0.0134
C	3.4440	0.6770	-0.6775	C	3.5290	0.7181	-0.5063
C	3.6777	-2.0259	-0.0169	C	3.7367	-2.0109	0.1225
C	-1.9042	-1.3351	-0.5216	C	-1.9069	-1.2525	-0.7377
C	-1.2781	1.2530	0.2942	C	-1.2331	1.1799	0.4844
C	1.3039	2.2774	0.0546	C	1.2493	2.2746	-0.0962
C	-2.9498	-0.4419	-0.1171	C	-2.9217	-0.4425	-0.1278
C	-2.6196	0.8793	0.2805	C	-2.5806	0.8125	0.4573
O	-1.0279	2.5504	0.6391	O	-0.9412	2.4246	0.9540
C	0.2056	3.1679	0.4241	C	0.1811	3.1191	0.3969
C	2.5667	2.8975	-0.3282	C	2.4603	2.9148	-0.5891
C	4.5978	-0.0407	-1.0712	C	4.7129	0.0346	-0.7748
C	4.6862	-1.3752	-0.7838	C	4.8096	-1.3225	-0.5051
C	-3.6888	1.8826	0.6586	C	-3.6319	1.7453	1.0042
O	0.2466	4.3609	0.6107	O	0.1302	4.3247	0.4793
O	2.8805	4.0624	-0.3077	O	2.6501	4.0860	-0.8309
H	5.3891	0.4979	-1.5800	H	5.5361	0.5882	-1.2134
H	5.5623	-1.9420	-1.0854	H	5.7245	-1.8612	-0.7295
H	4.7513	-3.8946	0.1010	H	4.7872	-3.8921	0.2480
H	3.1169	-4.9753	1.6212	H	3.0111	-5.0531	1.5229
H	1.1604	-3.6646	2.4468	H	0.9678	-3.7925	2.2149
H	0.7722	-1.3829	1.6738	H	0.6794	-1.4541	1.5578
C	-2.2351	-2.7382	-0.9895	C	-2.2593	-2.5718	-1.3805
N	-4.2639	-0.8466	-0.1592	N	-4.2318	-0.8606	-0.1311
C	-5.3134	-0.0076	0.4185	C	-5.2632	-0.0995	0.5780
H	-3.4118	2.8725	0.2867	H	-3.3565	2.7763	0.7694

compound 16

S0				S1			
	x	y	z		x	y	z
C	3.4901	-3.4240	0.2575	C	3.5440	-3.4131	0.2800
C	1.4472	-3.3451	1.5265	C	1.3895	-3.4423	1.3569
H	-3.2259	-2.5617	-2.1673	H	-3.2824	-2.2104	-2.5128
H	-3.1443	-3.2794	-0.5533	H	-3.0702	-3.1910	-1.0536
C	2.5654	-4.0633	1.0525	C	2.5542	-4.1131	0.9398

H	-5.7654	0.8907	-0.5984	H	-5.7482	0.8941	-0.1514
C	-7.0313	-0.7876	-0.0814	C	-6.9988	-0.8734	-0.0613
H	-5.6201	0.3641	1.0797	H	-5.5566	-0.0452	1.3301
H	-0.1665	-1.5664	-0.9375	H	-0.1487	-1.3489	-1.2775
C	1.2419	-2.0306	1.1587	C	1.2194	-2.0965	1.0685
C	0.7960	0.8623	0.0334	C	0.8530	0.8278	0.1081
C	-2.5983	-2.6532	-1.2708	C	-2.5944	-2.4299	-1.6861
O	3.1846	2.0220	-0.7654	O	3.1675	2.0823	-0.7133
C	1.9941	0.0330	-0.0996	C	2.0244	0.0549	0.0150
C	2.1443	-1.3550	0.2946	C	2.1830	-1.3633	0.3413
C	-0.9383	-0.8992	-0.5759	C	-0.9248	-0.7720	-0.7886
C	-0.5559	0.3594	-0.0423	C	-0.5489	0.3346	-0.0080
C	3.1214	0.6751	-0.6145	C	3.1858	0.7355	-0.4497
C	3.3239	-2.0636	-0.1085	C	3.4065	-2.0296	-0.0086
C	-2.2498	-1.3076	-0.6858	C	-2.2422	-1.1926	-0.9069
C	-1.6104	1.2318	0.2993	C	-1.5854	1.1314	0.5408
C	0.9733	2.2457	0.1604	C	0.8983	2.2524	0.0609
C	-3.2893	-0.4258	-0.2360	C	-3.2656	-0.4166	-0.2474
C	-2.9438	0.8507	0.2392	C	-2.9159	0.7594	0.4506
O	-1.3719	2.5085	0.7079	O	-1.2971	2.3212	1.1232
C	-0.1264	3.1238	0.5601	C	-0.1665	3.0557	0.6227
C	2.2467	2.8791	-0.1645	C	2.1038	2.9279	-0.3954
C	4.2792	-0.0266	-1.0246	C	4.3755	0.0783	-0.7573
C	4.3531	-1.3761	-0.8137	C	4.4794	-1.2926	-0.5786
N	-4.5893	-0.8330	-0.2897	N	-4.5586	-0.8172	-0.3377
H	-3.6950	1.5722	0.5323	H	-3.6659	1.4081	0.8835
O	-0.0777	4.3023	0.8200	O	-0.2197	4.2498	0.7970
O	2.5628	4.0403	-0.0806	O	2.2879	4.1134	-0.5609
H	5.0845	0.5349	-1.4842	H	5.1966	0.6644	-1.1557
C	-5.7334	0.0026	0.0498	C	-5.7050	-0.1284	0.2460
H	-4.7877	-1.6970	-0.7698	H	-4.7533	-1.6533	-0.8711
H	-1.6957	-3.1961	-1.5587	H	-1.6985	-2.8790	-2.1174
H	5.2322	-1.9307	-1.1286	H	5.3988	-1.8094	-0.8341
H	4.3824	-3.9442	-0.0799	H	4.4657	-3.9100	-0.0116
H	2.7083	-5.1007	1.3381	H	2.6846	-5.1687	1.1595
H	0.7434	-3.8259	2.1991	H	0.6280	-3.9722	1.9213
H	0.3880	-1.4992	1.5578	H	0.3331	-1.5933	1.4357
H	-7.1801	-1.1356	-1.1094	H	-7.1704	-0.9406	-1.1402
H	-7.8836	-0.1574	0.1840	H	-7.8439	-0.3408	0.3804
H	-7.0308	-1.6578	0.5825	H	-6.9827	-1.8850	0.3559

compound 17

S0				S1			
	x	y	z		x	y	z
H	5.3326	-1.2611	0.4372	H	5.3216	-0.9127	0.7491
H	3.4885	2.5482	-0.0358	H	3.3395	2.6358	-0.4999
H	2.8049	2.3200	-1.6370	H	2.7601	2.0952	-2.0655

H	5.3506	2.4148	-1.6289	H	5.2994	2.3253	-1.9525
H	4.7305	0.9043	-2.3041	H	4.7837	0.6930	-2.3853
H	5.7990	1.3492	0.5320	H	5.6846	1.6709	0.3779
H	6.5452	0.3706	-0.7363	H	6.5260	0.5116	-0.6575
C	5.5842	0.7065	-0.3354	C	5.5369	0.8824	-0.3756
C	4.8045	1.4985	-1.3861	C	4.7799	1.4378	-1.5818
C	-2.4781	3.9345	0.2970	C	-2.6815	3.8546	0.2905
C	-0.6019	3.3558	1.6881	C	-0.6568	3.4370	1.5267
C	-1.4927	4.3232	1.1765	C	-1.6346	4.3315	1.0534
H	0.7411	1.3217	-0.7461	H	0.6710	1.1605	-1.0833
C	-0.6722	2.0404	1.2764	C	-0.7224	2.0924	1.1924
C	-0.8128	-0.8462	0.0610	C	-0.8402	-0.8192	0.1309
O	-3.3360	-1.4066	-0.9427	O	-3.2768	-1.5797	-0.9092
C	-1.7789	0.2388	-0.1154	C	-1.8314	0.1676	-0.0133
C	-1.6398	1.6129	0.3282	C	-1.7439	1.5802	0.3628
C	1.3128	0.4840	-0.3664	C	1.2781	0.4377	-0.5497
C	0.6189	-0.6651	0.0917	C	0.6362	-0.5981	0.1497
C	-2.9816	-0.1145	-0.7291	C	-3.0600	-0.2623	-0.5918
C	-2.5971	2.5829	-0.1174	C	-2.7857	2.4796	-0.0482
C	2.6881	0.5809	-0.3773	C	2.6556	0.6074	-0.5277
C	1.4220	-1.7697	0.4591	C	1.4501	-1.5914	0.7508
C	-1.3097	-2.1546	0.1216	C	-1.1496	-2.2050	0.0196
C	3.4631	-0.5319	0.0774	C	3.4470	-0.3600	0.1790
C	2.8053	-1.7064	0.4830	C	2.8275	-1.4727	0.7903
O	0.8680	-2.9639	0.8086	O	0.8933	-2.7243	1.2477
C	-0.4728	-3.2698	0.5643	C	-0.3035	-3.2143	0.6229
C	-2.6635	-2.4715	-0.3182	C	-2.4226	-2.6229	-0.5503
C	-3.9184	0.8442	-1.1813	C	-4.0734	0.6203	-0.9589
C	-3.7012	2.1685	-0.9164	C	-3.9287	1.9803	-0.7292
O	-0.8109	-4.4080	0.7862	O	-0.4904	-4.4018	0.7423
O	-3.2376	-3.5326	-0.3098	O	-2.8157	-3.7454	-0.7778
H	-4.7942	0.4943	-1.7157	H	-4.9557	0.2151	-1.4423
H	-4.4077	2.9174	-1.2621	H	-4.7090	2.6719	-1.0297
H	-3.2034	4.6540	-0.0728	H	-3.4652	4.5280	-0.0468
H	-1.4184	5.3572	1.4983	H	-1.5803	5.3855	1.3095
H	0.1418	3.6442	2.4249	H	0.1419	3.7934	2.1703
H	0.0091	1.3171	1.7047	H	0.0185	1.4174	1.6037
C	3.4007	1.8277	-0.8615	C	3.3359	1.7791	-1.1904
N	4.8248	-0.4702	0.0715	N	4.7982	-0.2251	0.2233
H	3.3642	-2.5862	0.7837	H	3.4154	-2.2578	1.2530

compound 18

S0				S1			
	x	y	z		x	y	z
H	7.2784	1.5382	-0.7001	H	7.1929	0.6615	-1.8028
H	7.3851	-1.9027	-1.4074	H	7.6517	-2.1710	0.1249
H	7.3236	-2.4418	0.2854	H	7.6971	-1.3696	1.7106

H	8.6149	-1.3687	-0.2501	H	8.8510	-0.9100	0.4618
H	5.1162	1.6799	0.2983	H	5.0529	1.4041	-0.9775
H	2.8401	-2.6671	-0.5027	H	3.2463	-2.3031	1.7929
H	5.2673	-2.5806	-0.4572	H	5.6501	-2.0653	1.5383
C	4.7066	-1.6694	-0.2926	C	4.9990	-1.3533	1.0472
C	5.4019	-0.4342	-0.0497	C	5.5613	-0.2916	0.2627
O	0.3882	2.9676	0.2809	O	0.2195	2.1559	-1.3390
O	-1.3878	4.2061	-0.1370	O	-1.6102	3.4329	-1.3644
O	-3.8290	2.9729	-0.3011	C	3.2782	0.4695	-0.2232
N	-3.8616	0.6907	-0.2621	C	4.6793	0.5932	-0.3647
N	-4.0422	-1.6559	-0.1204	C	2.3765	1.3814	-0.8367
C	3.2253	0.6877	0.1112	H	2.7506	2.2099	-1.4294
C	4.6384	0.7219	0.1361	C	1.0204	1.2326	-0.6829
C	2.4352	1.8543	0.2557	C	-1.0871	2.4226	-0.9334
H	2.9011	2.8283	0.3639	C	-1.6867	1.4320	-0.0709
C	1.0606	1.7810	0.2178	C	-1.0071	0.1104	0.0943
C	-0.9723	3.0763	-0.0151	C	0.4535	0.1593	0.0751
C	-1.7297	1.8258	-0.0663	C	1.3295	-0.7160	0.7001
C	-3.1723	1.9403	-0.1957	H	0.9392	-1.5068	1.3329
C	-5.3135	0.7506	-0.4877	C	2.7314	-0.5938	0.5674
H	-5.5311	0.5202	-1.5384	C	3.6413	-1.4883	1.1912
H	-5.6073	1.7808	-0.2974	N	6.9346	-0.1570	0.1513
C	-5.9932	-0.2484	0.4378	C	7.8167	-1.2095	0.6332
H	-5.7542	-0.0031	1.4784	C	7.4774	0.8416	-0.7550
H	-7.0804	-0.2026	0.3279	H	8.5657	0.8331	-0.6893
C	-1.1097	0.5690	0.0981	H	7.1350	1.8462	-0.4814
C	0.3587	0.5456	0.0916	O	-2.9682	2.8900	1.3810
C	1.1379	-0.5907	-0.1192	N	-3.5813	0.6837	1.0864
H	0.6613	-1.5394	-0.3267	N	-4.3662	-0.9158	-0.4880
C	2.5450	-0.5558	-0.1116	C	-2.7967	1.7974	0.8646
C	3.3383	-1.7183	-0.3204	C	-4.7864	0.7133	1.9152
C	-1.8958	-0.6104	0.2686	H	-5.1250	1.7530	1.9368
C	-1.4137	-1.8078	1.0864	H	-4.5629	0.4198	2.9487
H	-0.4587	-1.5447	1.5421	C	-5.8589	-0.2108	1.3101
H	-2.1183	-1.9170	1.9236	H	-5.7849	-1.2144	1.7420
C	-1.2991	-3.1902	0.4191	H	-6.8562	0.1732	1.5444
H	-0.7116	-3.1236	-0.5062	C	-1.9075	-0.8989	0.1493
H	-0.7530	-3.8619	1.0919	C	-1.6766	-2.3823	0.2365
C	-2.6662	-3.7907	0.0975	H	-0.6214	-2.5844	0.4281
H	-2.5449	-4.7434	-0.4322	H	-2.2398	-2.7892	1.0889
H	-3.2131	-4.0080	1.0240	C	-2.0813	-3.1426	-1.0480
C	-3.5090	-2.8623	-0.7788	H	-1.5074	-2.7339	-1.8894
H	-2.9177	-2.5488	-1.6485	H	-1.7654	-4.1864	-0.9354
H	-4.3758	-3.4090	-1.1615	C	-3.5751	-3.1243	-1.3979
C	-5.5001	-1.6397	0.0724	H	-3.7242	-3.7388	-2.2944
H	-5.7676	-2.3743	0.8427	H	-4.1576	-3.5940	-0.5949

H	-5.9823	-1.9592	-0.8615	C	-4.1732	-1.7402	-1.6878
C	-3.2632	-0.5238	-0.0344	H	-3.5518	-1.1983	-2.4150
N	6.7814	-0.4131	-0.0012	H	-5.1585	-1.8663	-2.1426
C	7.5542	-1.5916	-0.3667	C	-5.6820	-0.3338	-0.2065
C	7.4701	0.8594	0.1438	H	-6.4406	-0.9985	-0.6254
H	8.5440	0.6826	0.2036	H	-5.7926	0.6488	-0.6924
H	7.1626	1.3702	1.0638	C	-3.2930	-0.3058	0.1019

compound 19

S0				S1			
	x	y	z		x	y	z
C	7.2718	0.9538	-0.0278	C	7.2669	1.0022	-0.1475
H	4.8802	1.6700	-0.1165	H	4.8655	1.6503	-0.1774
H	5.2408	-2.6360	0.1579	H	5.3005	-2.6463	0.2679
C	7.4763	-1.5347	0.1818	C	7.5238	-1.4883	0.0702
N	6.6464	-0.3589	-0.0369	N	6.6584	-0.3196	-0.0178
C	4.6360	-1.7403	0.0961	C	4.6941	-1.7554	0.1805
C	5.2696	-0.4529	-0.0002	C	5.2971	-0.4796	0.0319
C	4.4502	0.6778	-0.0595	C	4.4429	0.6594	-0.0680
C	-3.0350	-3.1238	-0.3470	C	-3.0649	-3.0929	-0.4109
C	-1.6667	-2.4264	-0.1159	C	-1.6982	-2.4353	-0.0958
C	-3.4452	1.5498	0.0379	C	-3.4554	1.5508	0.0780
C	-1.9890	1.4865	0.0093	C	-2.0001	1.4978	0.0332
C	-3.4150	-0.8759	-0.0054	C	-3.4189	-0.8782	0.0383
C	-1.2837	0.2463	-0.0246	C	-1.2743	0.2374	0.0057
C	-2.0232	-0.9471	-0.0641	C	-2.0436	-0.9533	-0.0376
C	-1.2934	2.7655	-0.0228	C	-1.3280	2.7577	-0.0256
C	0.8227	1.5638	-0.0777	C	0.8072	1.5354	-0.0700
C	0.1819	0.2892	-0.0223	C	0.1721	0.2658	0.0285
C	-5.5828	0.2701	0.0187	C	-5.5896	0.2913	0.0038
C	2.1931	1.7047	-0.0962	C	2.1966	1.6710	-0.1081
C	1.0182	-0.8240	0.0492	C	1.0340	-0.8544	0.1544
C	2.4221	-0.7195	0.0391	C	2.4417	-0.7518	0.1154
C	3.0403	0.5718	-0.0451	C	3.0382	0.5362	-0.0265
C	-6.1348	-1.0587	0.5342	C	-6.1837	-1.0578	0.4094
C	-5.4541	-2.2478	-0.1504	C	-5.4482	-2.2116	-0.2767
O	0.1024	2.7211	-0.1099	O	0.1147	2.6822	-0.1387
O	-4.1612	2.5463	0.0419	O	-4.1675	2.5554	0.0689
O	-1.7679	3.8796	0.0026	O	-1.7395	3.8988	-0.0102
N	-4.1092	0.2777	0.0533	N	-4.1245	0.2874	0.1220
N	-4.0183	-2.0932	0.0009	N	-4.0428	-2.1150	0.0779
H	-5.9202	1.1136	0.6205	H	-5.9663	1.1042	0.6251
H	-5.9089	0.4594	-1.0110	H	-5.8628	0.5328	-1.0327
H	-5.9786	-1.1308	1.6157	H	-6.1124	-1.1883	1.4946
H	-7.2125	-1.0913	0.3527	H	-7.2441	-1.0735	0.1408
H	-5.7558	-3.1908	0.3165	H	-5.8314	-3.1784	0.0656
H	-5.7342	-2.3011	-1.2147	H	-5.5945	-2.1663	-1.3715

H	-1.2176	-2.7718	0.8241	H	-1.3121	-2.8045	0.8651
H	-0.9718	-2.6771	-0.9217	H	-0.9631	-2.6900	-0.8637
H	-3.1607	-3.4235	-1.3973	H	-3.1831	-3.2376	-1.4975
H	2.6097	2.7056	-0.1426	H	2.6123	2.6689	-0.1994
H	0.6010	-1.8169	0.1281	H	0.6120	-1.8390	0.2939
C	3.2717	-1.8584	0.1128	C	3.3141	-1.8686	0.2187
H	-3.1732	-4.0111	0.2776	H	-3.2034	-4.0603	0.0804
H	2.8211	-2.8451	0.1854	H	2.8758	-2.8565	0.3332
H	8.3540	0.8376	-0.0877	H	8.3498	0.8936	-0.1492
H	6.9501	1.5499	-0.8899	H	6.9694	1.4895	-1.0827
H	7.0358	1.5228	0.8833	H	6.9897	1.6528	0.6892
H	8.5245	-1.2489	0.0939	H	8.5627	-1.1769	-0.0178
H	7.2828	-2.3088	-0.5697	H	7.3020	-2.1996	-0.7340
H	7.3277	-1.9790	1.1763	H	7.3918	-2.0044	1.0290

compound 20

S0				S1			
	x	y	z		x	y	z
H	-5.7857	-1.1454	0.2675	H	-5.7588	-1.2221	0.2637
H	-5.1814	-3.1946	-0.9897	H	-5.0871	-3.3506	-0.8079
H	-2.8752	-3.4326	-1.9110	H	-2.7647	-3.6145	-1.6855
C	-3.1223	-2.5789	-1.2867	C	-3.0512	-2.7133	-1.1518
H	-5.2126	1.0817	1.0971	H	-5.2643	1.1155	0.8626
C	1.6610	-2.7848	1.1618	C	1.5971	-2.8356	1.0591
C	0.2994	-2.1782	0.7302	C	0.2986	-1.9903	1.0335
C	2.1776	1.5677	-0.4695	C	2.2077	1.5733	-0.3598
C	0.7396	1.5899	-0.2514	C	0.7648	1.6250	-0.1150
C	2.0781	-0.7891	0.0995	C	2.0825	-0.7579	0.1518
C	-0.0110	0.3978	-0.0196	C	-0.0594	0.4563	-0.0010
C	0.6841	-0.8003	0.2068	C	0.6866	-0.7313	0.3074
C	0.1265	2.8955	-0.0971	C	0.1447	2.9523	-0.0048
C	-1.9484	1.7814	0.4632	C	-2.0091	1.8524	0.2850
C	-1.4687	0.5356	0.0674	C	-1.4735	0.5648	-0.0118
C	4.2706	0.2316	-0.3364	C	4.2825	0.1616	-0.4003
C	-3.2921	1.9815	0.8647	C	-3.3377	2.0555	0.6090
C	-2.4435	-0.4885	-0.2234	C	-2.4343	-0.5093	-0.2649
C	-3.8041	-0.2930	0.1860	C	-3.8104	-0.3013	0.1166
C	-4.1837	0.9464	0.7762	C	-4.2239	0.9694	0.5903
C	4.7634	-1.2112	-0.4440	C	4.6651	-1.3010	-0.6309
C	4.0867	-2.1103	0.5945	C	4.0483	-2.2167	0.4341
O	-1.1599	2.8837	0.4978	O	-1.1919	2.9663	0.3239
O	2.9131	2.5072	-0.7537	O	2.9404	2.5182	-0.6240
O	0.5851	3.9935	-0.3107	O	0.6938	4.0276	-0.1178
N	2.7971	0.2854	-0.2835	N	2.8193	0.2801	-0.2922
N	2.6489	-1.9757	0.4338	N	2.6257	-1.9264	0.5399
H	4.5856	0.8333	-1.1892	H	4.6044	0.7996	-1.2233
H	4.6662	0.7195	0.5616	H	4.7446	0.5495	0.5156

H	4.5553	-1.6033	-1.4451	H	4.3278	-1.6194	-1.6230
H	5.8473	-1.2272	-0.3022	H	5.7533	-1.3963	-0.6050
H	4.3598	-3.1587	0.4402	H	4.1678	-3.2707	0.1650
H	4.3956	-1.8334	1.6152	H	4.5306	-2.0668	1.4102
H	-0.1605	-2.8117	-0.0332	H	-0.5324	-2.5207	0.5621
H	-0.4041	-2.1312	1.5654	H	-0.0428	-1.7447	2.0487
H	1.8257	-2.6850	2.2446	H	1.8703	-3.1696	2.0650
H	-3.5772	2.9613	1.2313	H	-3.6661	3.0546	0.8729
C	-2.1561	-1.6307	-1.0152	C	-2.1126	-1.6937	-0.9521
C	-4.7662	-1.3018	-0.0747	C	-4.7322	-1.3692	-0.0619
H	1.7609	-3.8408	0.8967	H	1.5302	-3.7212	0.4159
C	-4.4331	-2.4357	-0.7833	C	-4.3578	-2.5563	-0.6737
H	-1.1740	-1.7241	-1.4614	H	-1.1209	-1.8041	-1.3798
